# Risk factor monitoring, management and use of prevention medicines in those with a history of premature coronary heart disease

**DOI:** 10.1136/openhrt-2024-003092

**Published:** 2025-09-11

**Authors:** Samia Kazi, Desi Quintans, Simone Marschner, Haeri Min, James Chong, Clara K Chow

**Affiliations:** 1 The University of Sydney, Sydney, New South Wales, Australia; 2 Westmead Hospital, Westmead, New South Wales, Australia; 3 Westmead Applied Research Centre, The University of Sydney, Westmead, New South Wales, Australia; 4 Westmead Applied Research Centre, The University of Sydney Faculty of Medicine and Health, Sydney, New South Wales, Australia; 5 Cardiology, Westmead Hospital, Westmead, New South Wales, Australia

**Keywords:** EPIDEMIOLOGY, CORONARY ARTERY DISEASE, Delivery of Health Care, Quality of Health Care, Risk Factors

## Abstract

**Background:**

Those with premature coronary heart disease (pCHD) have a lower 5-year risk of repeat events; however, their lifetime risk is high. The aim of this study was to assess secondary prevention (SP) medical therapy and risk factor (RF) monitoring in patients with pCHD compared with those without pCHD.

**Methods:**

Analysis of a national primary care database including patients attending the same practice between January 2015 and March 2021 with at least 3 follow-up appointments, a history of CHD and a follow-up duration of at least 2 years. pCHD was defined as males under 55 and females under 65 at age of diagnosis of their CHD.

**Results:**

Among the 64 704 with CHD, 21 035 (32.5%) had pCHD (10 339 women <65 years of age and 10 696 men <55 years of age). Patients with pCHD compared with non-pCHD were more likely to be smokers (59.4% vs 52.6%, p<0.001), less likely to have hypertension (61.9% vs 73.2%, p<0.001) and similar rates of dyslipidaemia (57.8% and 57.5%, p=0.806) and diabetes (30.8% vs 30%, p=0.696). After adjusting for RF, pCHD was not associated with odds of blood pressure (BP) assessment (OR 0.63, 95% CI 0.40 to 1.00), measurement of glycated haemoglobin (OR 0.99, 95% CI 0.94 to 1.04) and any lipid monitoring (OR 1.05, 95% CI 0.96 to 1.14). Patients with PCHD had lower odds of prescription of statins (OR 0.81, 95% CI 0.76 to 0.87), any antiplatelets (OR 0.81, 95% CI 0.77 to 0.86), antihypertensive medication (OR 0.73, 95% CI 0.67 to 0.79) and beta blockers (OR 0.94, 95% CI 0.90 to 0.98) after adjustment for baseline RF. Women with pCHD were even less likely to be prescribed SP medicines of BP lowering and antiplatelets, both p value for interaction <0.000.1

**Conclusions:**

Patients with a history of pCHD had similar rates of RF monitoring to patients without pCHD, but patients with pCHD were less likely to be prescribed SP medication of statins, antihypertensives and antiplatelets.

**WHAT IS ALREADY KNOWN ON THIS TOPIC**

- Premature coronary artery disease has a significant long-term disease burden on the individual and society as a whole. Secondary prevention is key in this high-risk population given their high risk of repeat events. The risk factor profile of this demographic is also less well defined. A greater understanding of current screening of risk factors and secondary prevention is required to address deficiencies in our system.

**WHAT THIS STUDY ADDS**

- The study illustrates that patients with premature coronary heart disease (pCHD) were more likely to be smokers, had similar rates of diabetes and hypercholesterolaemia but were less likely to have hypertension.
- This study identifies a significant under prescription of secondary prevention therapy in patients with a history of pCHD. However, no differences in risk factor surveillance were found in those with pCHD in primary care. These findings remained significant after accounting for cardiovascular risk factor levels.

**HOW THIS STUDY MIGHT AFFECT RESEARCH, PRACTICE OR POLICY**

- This study identifies significant gaps in management of patients with pCHD and underscores the importance of looking for these gaps to achieving high quality secondary prevention.
- As risk factor monitoring was occurring at a similar rate in people with and without pCHD, it suggests that such visits could be used to enhance the quality of secondary prevention therapy.
- Differences in risk factor profile and greater prevalence of obesity and substance use of those with and without pCHD suggest that such risk factors should be screened for and managed in patients with pCHD.

## Introduction

 Premature coronary heart disease (pCHD), defined as CHD in females <65 and males <55, is poorly described in current literature.^1 2^ The incidence of pCHD has remained unchanged or even increased over time^3 4^ despite devastating consequences to individuals and society from a longer duration of disease burden. The Australian Bureau of Statistics estimates 11.2 life years lost in each individual with pCHD,^5^ with a significant effect on the economy as this age group provides the bulk of the workforce and is the predominant age of caregivers.^6^ Furthermore, pCHD is the largest cause of sudden cardiac death in those aged over 35 in Australia.^7^

Although overall CHD-related mortality has reduced in the general population, this has not been reflected in the pCHD population.^8^ In an analysis of US trends in myocardial infarction (MI) from 1979 to 2011, a reduction in mortality in those aged over 65 was observed, but mortality rates were stagnant among those aged <65 years, with a particularly slower decline observed in women.^8^ This is notable as the burden among patients with pCHD is large. One study reported the all-cause mortality rate was 20.9% at 10 years for those <50, and the risk of one recurrent ischaemic event was 52.9%, 18.6% had a second recurrent event and 7.9% had a third event.^9^ Other studies demonstrated that new coronary lesions were responsible for recurrent events in two of three patients with pCHD, demonstrating the aggressive nature of the disease.^10^

This highlights the critical need for secondary prevention (SP) therapy in this high-risk population. Screening for risk factors^11^ (RFs) and ensuring adequate targets of blood pressure (BP), body mass index (BMI), cholesterol and glycated haemoglobin (HbA1c) targets are key for thorough SP.^12^ Medical SP universally requires lipid-lowering therapy, antiplatelets and antihypertensive medications for those with established CHD.^13^

The aim of this study was to (1) describe patients with pCHD compared with those without pCHD in a primary care community cohort and (2) examine the relationship of a history of pCHD with the likelihood of RF monitoring and SP medical management.

## Methods

### Population

We analysed the MedicineInsight Database, a large Australian general practitioner (GP) longitudinal deidentified electronic health record database. The National Prescribing Service (NPS) extracted patients from the MedicineInsight database with documented cardiovascular disease (CVD), encompassing 141 929 electronic health records from 406 GP sites. It was established in 2011 with the aim to improve quality outcomes in primary care and has been validated across multiple other Australian datasets as a true representation of the Australian demographics.^14^

### Inclusion

Patients included ≥18 and had a diagnosis of CHD with at least 2 years follow-up data from the time of CHD diagnosis (online supplemental figure 1). Only active patients, classified by the Royal Australian College of General Practitioners as patients who had attended the practice ≥3 times in the past 2 years and had at least one visit between 1 January 2015 and 1 March 2021 were included. CHD was defined according to the prespecified conditions codes, defined by the NPS (online supplemental file). Patients with no documented sex, age or date of CHD diagnosis were excluded.

Patients were stratified as having pCHD by age at diagnosis (<65 in women or <55 men). We defined traditional RFs as diabetes, hypercholesterolaemia, current smoker (not an ex-smoker or never smoked) and hypertension. Data cover encounters from the time the patient first attended the GP practice to 1 March 2021. Analysis of biochemical RFs, BMI and BP was assessed using data after the diagnosis of CHD and after January 2011. Medication prescription was determined based as ever prescribed.

We defined other RFs including cancer, substance abuse and chronic kidney disease (stages 3–5) using the coded terms defined by the NPS. Chronic inflammatory conditions were defined from a string search of arthritis codes from the dataset and included: “ANKYLOSING SPONDYLITIS”, “LUPUS”, “PSORIATIC”, “RHEUMATOID”, “SERONEGATIVE”, “INFLAMMATORY POLYARTHRITIS”, “RA” and “SERONEGATIVE”.

Outcomes of interest were RF assessment and use of SP medicines. RFs included BP, lipid profile, HbA1c and BMI. Lipid profile included low-density lipoprotein (LDL) monitoring, high-density lipoprotein (HDL), triglycerides and total cholesterol. SP medicines included use of antiplatelets, cholesterol-lowering statins and BP-lowering therapy (online supplemental table 1). Furthermore, assessment of those not on statins but on alternative lipid-lowering therapy was incorporated (online supplemental table 1).

### Statistical analysis

Statistical analyses were performed using IBM SPSS Statistics (V.26), Microsoft Excel V.2311 and R V.4.3.2. using the packages gtsummary V.1.7.2, lme4 V.1.1–35.1 and forestplot V.3.1.3. P values of less than 0.05 are considered statistically significant. Given the large sample size, statistical significance must be interpreted carefully in the context of clinically meaningful differences. Characteristics are presented as means and CIs for normally distributed data, medians and IQRs for skewed data or percentages. A mixed effects logistic regression model with a random effect for GP clinic and fixed effects for other variables was used to analyse the determinants of RF assessment and SP. Differences in duration of follow-up were adjusted for by including an offset of the log of the follow-up time (CHD diagnosis to last clinical visit). Missing dates or dates outside of this period (after their CHD diagnosis and/or within the last 10 years) of RF assessment or prescription were excluded from this analysis. The models were adjusted for sex, presence or absence of hypertension, diabetes, current smoking, First Nations status, substance abuse, inflammatory diseases, stage 3 CKD or higher, dyslipidaemia and a history of heart failure or cancer or peripheral vascular disease.

## Results

Of the 64 704 patients with CHD, 21 035 (32.5%) had pCHD (10 339 women <65 years of age and 10 696 men <55 years of age) and 43 669 did not have pCHD (13 133 women and 30 536 men; table 1).

**Table 1:** Demographics, modifiable RFs and medical history

Characteristic	Overall				
	CHD, N=43 669*	95% CI	pCHD, N=21 035*	95% CI	P value†
Aboriginal and/or Torres Strait Islander	679 (1.6%)	1.4% to 1.7%	1150 (5.5%)	5.2% to 5.8%	<0.001
Age	78 (72, 84)		63 (56, 70)		
Number of clinical encounters	117 (61, 203)		87 (43, 160)		<0.001
Remoteness Area (2016)					0.002
Major Cities of Australia	24 840 (57.1%)	56.6% to 57.5%	11 504 (54.9%)	54.2% to 55.6%	
Inner Regional Australia	12 399 (28.5%)	28.1% to 28.9%	5957 (28.4%)	27.8% to 29.0%	
Outer Regional Australia	5648 (13.0%)	12.7% to 13.3%	3118 (14.9%)	14.4% to 15.4%	
Remote and Very Remote Australia	641 (1.5%)	1.4% to 1.6%	384 (1.8%)	1.7% to 2.0%	
Unknown	141		72		
IRSAD (2016) quintile					<0.001
1	9573 (22.0%)	21.6% to 22.4%	4893 (23.3%)	22.8% to 23.9%	
2	9059 (20.8%)	20.4% to 21.2%	4397 (21.0%)	20.4% to 21.5%	
3	9418 (21.6%)	21.3% to 22.0%	4715 (22.5%)	21.9% to 23.1%	
4	6880 (15.8%)	15.5% to 16.2%	3414 (16.3%)	15.8% to 16.8%	
5	8598 (19.8%)	19.4% to 20.1%	3544 (16.9%)	16.4% to 17.4%	
Unknown	141		72		
Traditional modifiable risk factors					
Diabetes	13 110 (30.0%)	29.6% to 30.5%	6487 (30.8%)	30.2% to 31.5%	0.696
Dyslipidaemia	25 095 (57.5%)	57.0% to 57.9%	12 163 (57.8%)	57.2% to 58.5%	0.806
Hypertension	31 986 (73.2%)	72.8% to 73.7%	13 011 (61.9%)	61.2% to 62.5%	<0.001
Smoking history					<0.001
Never	19 974 (47.4%)	46.9% to 47.9%	8276 (40.6%)	39.9% to 41.3%	
Current or past	22 180 (52.6%)	52.1% to 53.1%	12 112 (59.4%)	58.7% to 60.1%	
Unknown	1515		647		
Risk enhancing factors					
BMI category (highest)					<0.001
Healthy weight range	5279 (14.8%)	14.4% to 15.2%	1945 (11.3%)	10.9% to 11.8%	
Obese	16 824 (47.2%)	46.7% to 47.7%	10 239 (59.7%)	58.9% to 60.4%	
Overweight	13 385 (37.6%)	37.1% to 38.1%	4891 (28.5%)	27.8% to 29.2%	
Underweight	142 (0.4%)	0.3%to 0.5%	81 (0.5%)	0.4% to 0.6%	
Unknown	8039		3879		
Any substance abuse	1651 (3.8%)	3.6% to 4.0%	1535 (7.3%)	7.0% to 7.7%	<0.001
Cancer	19 783 (45.3%)	44.8% to 45.8%	6196 (29.5%)	28.8% to 30.1%	<0.001
Chronic inflammatory arthritic disease	5652 (12.9%)	12.6% to 13.3%	2270 (10.8%)	10.4% to 11.2%	<0.001
Chronic kidney disease (stages 3–5)	3030 (6.9%)	6.7% to 7.2%	745 (3.5%)	3.3% to 3.8%	<0.001
Heart failure	7120 (16.3%)	16.0% to 16.7%	2026 (9.6%)	9.2% to 10.0%	<0.001
Peripheral vascular disease	3132 (7.2%)	6.9% to 7.4%	882 (4.2%)	3.9% to 4.5%	<0.001

* n (%); Median (IQR).

† Random intercept logistic regression.

CHD, coronary heart disease; pCHD, premature coronary heart disease; RF, risk factor.

Patients with pCHD compared with those without pCHD were more likely to be smokers (59.4%, 95% CI: 58.7% to 60.1%) vs 52.6%, 95% CI 52.1% to 53.1%), p<0.001; less likely to have a history of hypertension (61.9%, 95% CI 61.2% to 62.5% vs 73.2%, 95% CI 72.8% to 73.7%, p<0.001) and similar rates of dyslipidaemia (57.8%, 95% CI 57.2% to 58.5% vs 57.5%, 95% CI 57% to 57.9%, p=0.806) and diabetes (30.8%, 95% CI 30.2% to 31.5% vs 30%, 95% CI 29.6% to 30.5%, p=0.696) (table 1).

RF differences (pCHD vs no pCHD) were accentuated among females as compared with males. The magnitude of the difference in diabetes was greater between pCHD females compared with females with no pCHD (30.0%, 95% CI 29.1% to 30.9% vs 26.4%, 95% CI 25.7% to 27.2%), p<0.001), in contrast to males who had higher overall rates of diabetes but less difference by pCHD (31.7%, 95% CI 30.8% to 32.5% vs 31.6%, 95% CI 31% to 32.1%) (interaction p<0.001). Males had a significant difference in proportion of smokers between pCHD and no pCHD (66.7%, 95% CI 65.7% to 67.6% vs 59.8%, 95% CI 59.3% to 60.4%, p<0.001) but females with pCHD had nearly one and a half times the rate of smoking (51.9%, 95% CI 50.9% to 52.9% vs 35.7%, 95% CI 34.9% to 36.5%, p<0.001) as compared with those without a history of pCHD (interaction p<0.001) (online supplemental table 2). PCHD males had a greater proportion of dyslipidaemia as compared with men without pCHD (58.0%, 95% CI 57.1% to 60.4% vs 56.6%, 95% CI 56% to 57.2%), which was the opposite to the pattern in women where women without pCHD had a greater proportion of dyslipidaemia compared with those with PCHD (57.6%, 95% CI 56.6% to 58.6% vs 59.5%, 95% CI 58.6% to 60.3%) (interaction p<0.001). Hypertension was represented in the older population more than the PCHD group in both sexes, and this difference was accentuated among women as compared with men (interaction p<0.001) (online supplemental table 2).

Of the additional RFs that were explored in both sexes, the most common factors were obesity and substance abuse in the pCHD group. The most prevalent additional RFs in people without pCHD were overweight or obesity, chronic kidney disease, cancer and chronic inflammatory disease (table 1).

Patients with CHD had high levels of BP assessment during GP visits subsequent to their diagnosis, whether they had pCHD or not (98.4%, 95% CI 98.2% to 98.5% vs 98.3%, 95% CI 98.2% to 98.4%), p=0.885) and there was also no difference in recording of BMI (81.6%, 95% CI 81.0% to 82.1% in patients with pCHD vs 81.6%, 95% CI 81.2% to 82.0%) in non-pCHD patients. Both groups also had similar levels of HbA1c being assessed (66.5%, 95% CI 65.9% to 67.1% vs 66.5%, 95% CI 65.9% to 66.8%), p=0.485 and similar rates of measurement of any lipids (91.1%, 95% CI 90.7% to 91.5% vs 91.1%, 95% CI 90.8% to 91.3%), p=625. Those with pCHD were less likely to have had each of their lipid parameters, including LDL, HDL, triglycerides and total cholesterol assessed (94.6%, 95% CI 94.3% to 94.9% vs 95.1%, 95% CI 94.9% to 95.3%), p=0.017 (table 2).

**Table 2:** Clinical and biochemical RF assessment

Characteristic	Male					Female					Overall				
	CHD, N=30 536*	95% CI	pCHD, N=10 696*	95% CI	P value†	CHD, N=13 133*	95% CI	pCHD, N=10 339*	95% CI	P value†	CHD, N=43 669*	95% CI	pCHD n=21 035*	95% CI	P value†
Measured HDL	28 383 (92.9%)	92.7% to 93.2%	9894 (92.5%)	92.0% to 93.0%	0.169	11 642 (88.6%)	88.1% to 89.2%	9477 (91.7%)	91.1% to 92.2%	<0.001	40 025 (91.7%)	91.4% to 91.9%	19 371 (92.1%)	91.7% to 92.4%	0.058
Measured LDL	28 210 (92.4%)	92.1% to 92.7%	9771 (91.4%)	90.8% to 91.9%	0.001	11 555 (88.0%)	87.4% to 88.5%	9390 (90.8%)	90.2% to 91.4%	<0.001	39 765 (91.1%)	90.8% to 91.3%	19 161 (91.1%)	90.7% to 91.5%	0.617
Measured triglycerides	29 057 (95.2%)	94.9% to 95.4%	10 058 (94.0%)	93.6% to 94.5%	<0.001	12 154 (92.5%)	92.1% to 93.0%	9728 (94.1%)	93.6% to 94.5%	<0.001	41 211 (94.4%)	94.2% to 94.6%	19 786 (94.1%)	93.7% to 94.4%	0.128
Measured total cholesterol	29 235 (95.7%)	95.5% to 96.0%	10 106 (94.5%)	94.0% to 94.9%	<0.001	12 292 (93.6%)	93.2% to 94.0%	9799 (94.8%)	94.3% to 95.2%	<0.001	41 527 (95.1%)	94.9% to 95.3%	19 905 (94.6%)	94.3% to 94.9%	0.017
Measured HbA1c	20 748 (67.9%)	67.4% to 68.5%	7161 (67.0%)	66.0% to 67.8%	0.020	8230 (62.7%)	61.8% to 63.5%	6827 (66.0%)	65.1% to 66.9%	<0.001	28 978 (66.4%)	65.9% to 66.8%	13 988 (66.5%)	65.9% to 67.1%	0.485
Measured any lipids	28 210 (92.4%)	92.1% to 92.7%	9771 (91.4%)	90.8% to 91.9%	0.001	11 554 (88.0%)	87.4% to 88.5%	9389 (90.8%)	90.2% to 91.4%	<0.001	39 764 (91.1%)	90.8% to 91.3%	19 160 (91.1%)	90.7% to 91.5%	0.625
Measured blood pressure	30 079 (98.5%)	98.4% to 98.6%	10 498 (98.1%)	97.9% to 98.4%	0.011	12 859 (97.9%)	97.7% to 98.1%	10 196 (98.6%)	98.4% to 98.8%	<0.001	42 938 (98.3%)	98.2% to 98.4%	20 694 (98.4%)	98.2% to 98.5%	0.885
Measured BMI	24 947 (81.7%)	81.3% to 82.1%	8661 (81.0%)	80.2% to 81.7%	0.007	10 683 (81.3%)	80.7% to 82.0%	8495 (82.2%)	81.4% to 82.9%	0.961	35 630 (81.6%)	81.2% to 82.0%	17 156 (81.6%)	81.0% to 82.1%	0.025

* n (%).

† Random intercept logistic regression.

CHD, coronary heart disease; HbA1c, glycated haemoglobin; HDL, high-density lipoprotein; LDL, low-density lipoprotein; pCHD, premature coronary heart disease; RF, risk factor.

**Figure 1:**
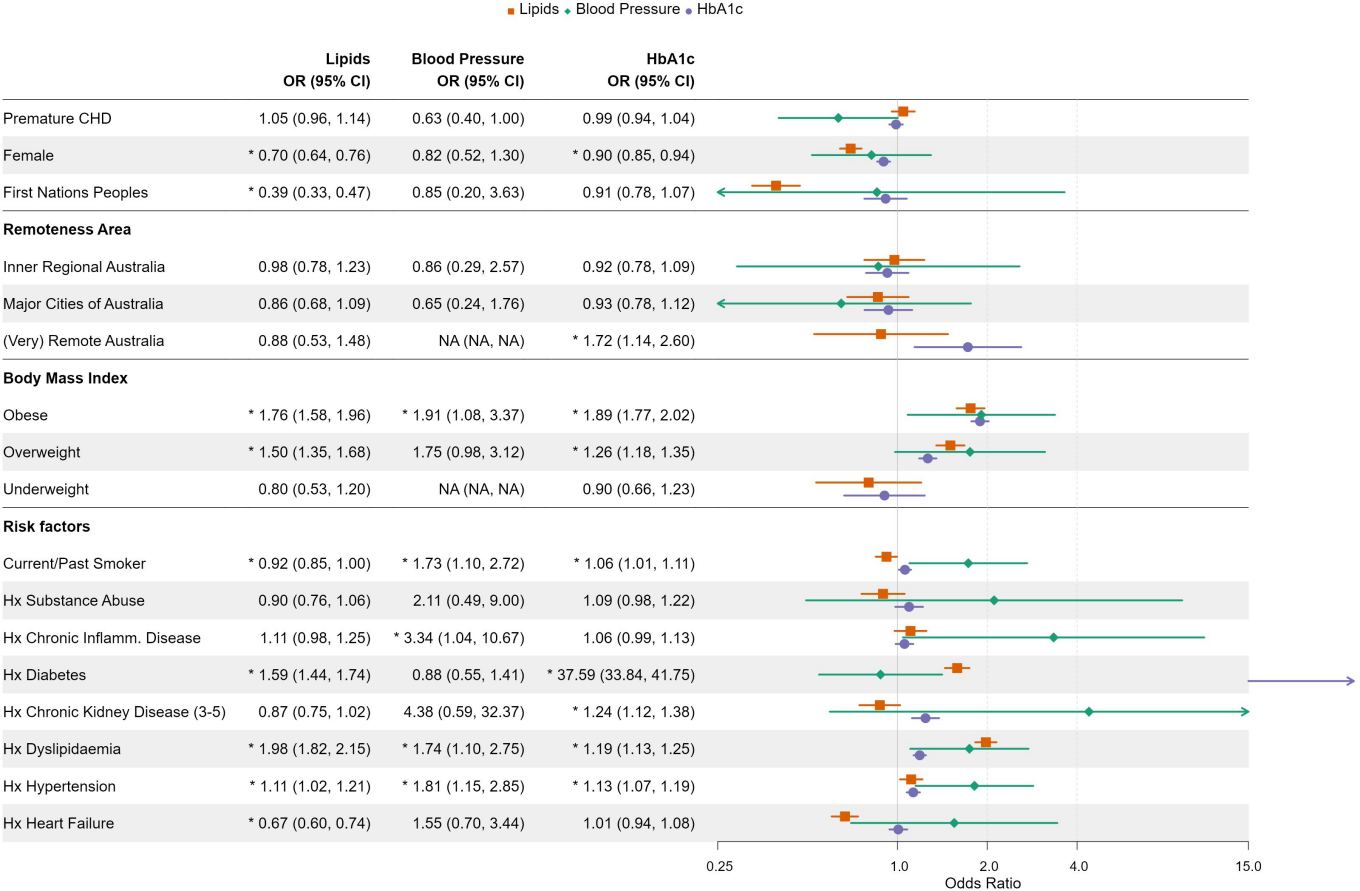
Forest plot of predictors of assessment of cardiovascular risk factors, screening of any lipids, blood pressure assessment and HbA1c assessment within 2 years of diagnosis of CHD. This has been adjusted for covariates and random effect applied for GP practice. The bars for HbA1c measurement in diabetics are outside the bands of this forest plot. CHD, coronary heart disease; GP, general practitioner; HbA1c, glycated haemoglobin.

In an adjusted multivariate model, pCHD was not a predictor of assessment of BP, lipids or HbA1c (figure 1). Females, First Nations people, those with a smoking history and those with a history of heart failure were less likely to have an assessment of their lipid profile. In the adjusted model, females, diabetics and those with a smoking history were less likely to have their HbA1c assessed. Acknowledging the high BP measurement proportion, we found no predictor of BP assessment.

There was less prescription of statins in the pCHD as compared with the no pCHD group (86.8%, 95% CI 86.3% to 87.3%) vs 90.2%, 95% CI 89.9% to 90.5%, p≤0.001) and any antiplatelet medications (81.4%, 95% CI 80.9% to 81.9% vs 85.8%, 95% CI 85.5% to 86.2%, p<0.001) (table 3). Of the patients who were on statins, 91.9% of patients with pCHD and 89.4% of patients with CHD were on the higher potency statins (atorvastatin and rosuvastatin). Similarly, pCHD had fewer prescriptions of ACE inhibitor (ACEi)/ARB, beta blockers and any BP-lowering medications (table 3). Of those who were not on statins but were on other non-statin lipid-lowering therapy, the pCHD group had a lower rate of prescription as compared with the non-pCHD group (6.7%, 95% CI 5.8% to 7.7% vs 10.4%, 95% CI 9.6% to 11.4%, p<0.001) (table 4).

**Table 3:** Prescriptions to secondary preventive medications

Characteristic	Overall				
	CHD N=43 669*	95% CI	pCHD N=21 035*	95% CI	P value†
Prescribed to ACEi	36 048 (82.5%)	82.2% to 82.9%	15 863 (75.4%)	74.8% to 76.0%	<0.001
Prescribed to aspirin	35 182 (80.6%)	80.2% to 80.9%	16 157 (76.8%)	76.2% to 77.4%	<0.001
Prescribed to beta blockers	28 641 (65.6%)	65.1% to 66.0%	12 805 (60.9%)	60.2% to 61.5%	<0.001
Prescribed to statins	39 390 (90.2%)	89.9% to 90.5%	18 259 (86.8%)	86.3% to 87.3%	<0.001
Prescribed to P2Y12 inhibitors	19 819 (45.4%)	44.9% to 45.9%	8824 (41.9%)	41.3% to 42.6%	<0.001
Prescribed to SA nitrates	21 211 (48.6%)	48.1% to 49.0%	10 032 (47.7%)	47.0% to 48.4%	0.004
Prescribed to aspirin and/or P2Y12 inhibitors	37 488 (85.8%)	85.5% to 86.2%	17 126 (81.4%)	80.9% to 81.9%	<0.001
Prescribed to any antihypertensive	40 673 (93.1%)	92.9% to 93.4%	18 321 (87.1%)	86.6% to 87.5%	<0.001

* n (%).

† Random intercept logistic regression.

ACEi, ACE inhibitor; CHD, coronary heart disease; pCHD, premature coronary heart disease.

**Table 4:** Prescriptions to non-statin lipid-lowering therapy among participants not on statins

Characteristic	Male					Female					Overall				
	CHD N=2351*	95% CI	pCHD N=1014*	95% CI	P value†	CHD N=1928*	95% CI	pCHD N=1762*	95% CI	P value†	CHD N=4279*	95% CI	pCHD N=2776*	95% CI	P value†
Prescribed to any non-statin lipid-lowering therapy	246 (10.5%)	9.3% to 11.8%	76 (7.5%)	6.0% to 9.3%	0.008	201 (10.4%)	9.1% to 11.9%	111 (6.3%)	5.2% to 7.6%	<0.001	447 (10.4%)	9.6% to 11.4%	187 (6.7%)	5.8% to 7.7%	<0.001
Prescribed to fibrates	109 (4.6%)	3.8% to 5.6%	41 (4.0%)	3.0% to 5.5%	0.382	78 (4.0%)	3.2% to 5.0%	38 (2.2%)	1.6% to 3.0%	0.001	187 (4.4%)	3.8% to 5.0%	79 (2.8%)	2.3% to 3.6%	<0.001
Prescribed to PCSK9 inhibitor	8 (0.3%)	0.2% to 0.7%	5 (0.5%)	0.2% to 1.2%	0.523	5 (0.3%)	0.1% to 0.6%	5 (0.3%)	0.1% to 0.7%	0.892	13 (0.3%)	0.2% to 0.5%	10 (0.4%)	0.2% to 0.7%	0.632
Prescribed to ezetimibe	173 (7.4%)	6.4% to 8.5%	41 (4.0%)	3.0% to 5.5%	<0.001	139 (7.2%)	6.1% to 8.5%	74 (4.2%)	3.3% to 5.3%	<0.001	312 (7.3%)	6.5% to 8.1%	115 (4.1%)	3.4% to 5.0%	<0.001
Prescribed to cholestyramine or nicotinic acid	15 (0.6%)	0.4% to 1.1%	0 (0.0%)	0.0% to 0.5%	0.001	15 (0.8%)	0.5% to 1.3%	9 (0.5%)	0.2% to 1.0%	0.350	30 (0.7%)	0.5% to 1.0%	9 (0.3%)	0.2% to 0.6%	0.031

* n (%).

† Random intercept logistic regression.

CHD, coronary heart disease; pCHD, premature coronary heart disease.

**Figure 2:**
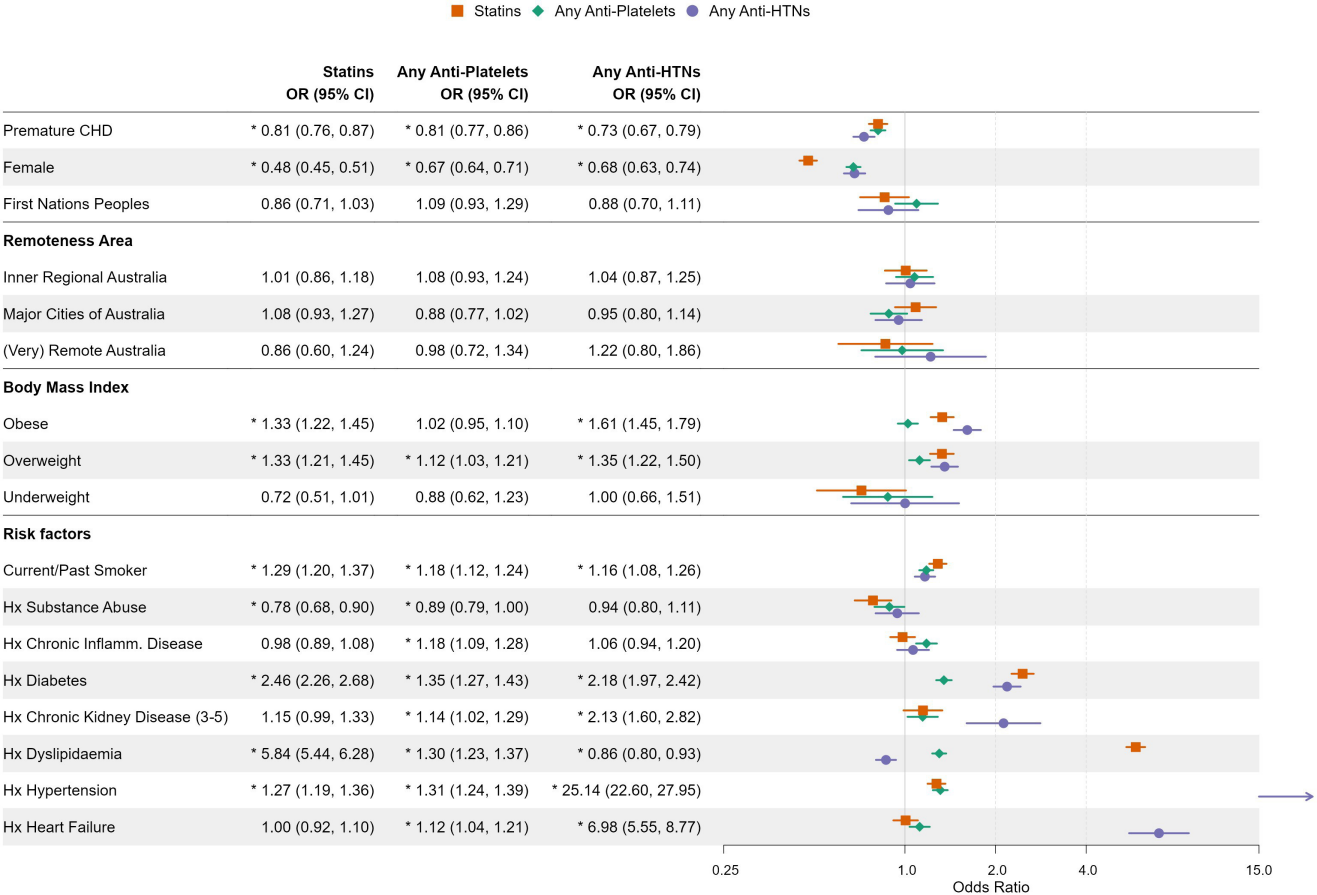
Forest plot of significant predictors of secondary prevention medical therapy. This has been adjusted for covariates and random effect applied for GP practice. The bars for statin therapy in dyslipidaemia, anti-hypertensive therapy in those with hypertension and heart failure are outside the bands of this forest plot. CHD, coronary heart disease; GP, general practitioner.

When adjusted in a multivariate model, the lower rates of prescription in the pCHD group of statins (OR 0.81, 95% CI 0.76 to 0.87) and antiplatelets (OR 0.81, 95% CI 0.77 to 0.86) remained significant (figure 2). Also in multivariable models, people with a history of pCHD were less likely to be prescribed any BP-lowering medications (OR 0.73, 95% CI 0.67 to 0.79), beta blockers (OR 0.94, 95% CI 0.90 to 0.98) but not ACEi/ARB (OR 0.96, 95% CI 0.90 to 1.02) (figure 2, online supplemental tables 3–5). In the multivariate model, the higher rates of prescription of non-statin lipid-lowering medication in the pCHD group remained statistically significant (OR 0.66, 95% CI 0.51 to 0.84, online supplemental table 5).

In all groups except for non-statin lipid-lowering therapy, females were less likely to be prescribed SP as compared with men. Females with a history of pCHD were less likely to be prescribed some SP medication as compared with females without pCHD, and this difference was more pronounced in ACEi prescription, men (pCHD (78.5%) vs CHD (82.8%)) compared with women (pCHD (72.2%) vs CHD (81.9%)), (interaction <0.001), BB men (pCHD (65%) vs CHD (66%)) vs women (pCHD (56.6%) vs CHD (64.7%)), (interaction <0.001), any antiplatelets men (pCHD (84.3%) vs CHD (87.2%)) vs women (pCHD (78.5%) vs CHD (82.7%)), (interaction p<0.001) and antihypertensive men (pCHD (88.5%) vs CHD (93.0%)) and women (pCHD (85.6%) vs CHD (93.4%), p<0.001) (online supplemental table 6). There is no difference in prescription of statins between the pCHD groups across both sexes, men (pCHD (90.5%) vs CHD (92.3%)) and women (pCHD (83.0%) vs CHD (85.3%), interaction p=0.432) (online supplemental table 6).

## Discussion

Almost one-third of patients with CHD in this primary care population had a history of pCHD. PCHD was associated with a lower odds of SP medical therapy prescription after accounting for differences in baseline RFs, though there was no significant difference in the odds of having RF monitoring after CHD diagnosis. The prescription of BP-lowering and antiplatelet SP medicines was lowest among women with pCHD. A potential explanation for this may be the perception by health practitioners that patients with pCHD have a lower overall risk of recurrent CHD events leading to less prescription of SP medicines. Findings like this might explain the relative lack of improvement in mortality outcomes in people with pCHD, despite improvements in mortality in those over the age of 65 years over the years.

Patients with pCHD had a different pattern of RFs, compared with non-pCHD patients. They were more likely to be smokers, but less likely to have hypertension and had similar rates of dyslipidaemia and diabetes. Although it has been well described that patients with pCHD have at least one RF, the pattern of risk enhancing factors is less well defined.^1 15^ In addition to differences in the traditional RFs described above, those with pCHD were more likely to have obesity and substance abuse. Smoking is often the most common RF in pCHD as demonstrated in several international studies.^4 9 10^ A higher proportion of substance abuse was also reported in the Young MI registry across two centres.^4 16^ A study of the Veterans Affairs dataset demonstrated those with substance abuse had 1.5–3 times greater odds of CHD aged <40.^17^ This suggests the overall RF profile of those with pCHD is different to the traditional profile public health campaigns currently target, and further research into the mechanisms of pCHD is required to identify those most vulnerable to allow targeted primary and secondary health campaigns.

Although studies in pCHD have demonstrated variability in the proportion of those with metabolic RFs, our study demonstrated a greater proportion of those with diabetes and obesity in pCHD, particularly in females.^3 4 18^ In addition, the rates of diabetes and obesity are increasing worldwide and will likely contribute to further pCHD.^19 20^ Metabolic RFs are especially important in the young, those under 50 with MI, as they have been found to have a three times greater likelihood of 6-year MACE and seven times greater likelihood of recurrent MI than those over the age of 50 with MI.^21^ The introduction of newer therapies to address obesity, diabetes and their contribution to CVD, including GLP1-receptor-agonists, may change this trend.^22^

Monitoring cardiometabolic RFs and hypertension is key in SP. Our study found no difference in screening of clinical RFs of BP and BMI between those with pCHD and older patients. Similarly, there is no significant difference in screening of biochemical RFs. Females, however, were less likely to have their lipid profile or HbA1c assessed in the adjusted model. The cause is unclear as females had a similar proportion of dyslipidaemia as compared with males and older females had similar rates of diabetes.

Some studies have specifically examined SP therapies in patients with pCHD and found differences in prescription. The PARTNERS Young MI study demonstrated that of 2097 MI patients, those <40 were less likely to be prescribed aspirin and statins at discharge than those aged 40–50.^4^ This study also demonstrated that these very young patients <40 had a similar incidence of mortality at 11 years.^4^ Another prospective multicentre study of 880 patients with CAD, aged ≤45, demonstrated that there was no difference in those prescribed aspirin or statins with recurrent events.^10^ Our study demonstrates underprescription of not only statins, which have the highest evidence in SP, but all other forms of lipid-lowering therapy in the pCHD group. This may change with rising use of PCSK9 inhibitors and other lipid-lowering therapy.

In addition to statins, antiplatelet therapy has also demonstrated risk reduction in those with established and pCHD.^23^ A study of 101 061 patients <50 with obstructive CAD demonstrated the high risk of disease recurrence with 10 events per 100 patient years and 1 in 5 patients dying within 10 years of follow-up.^9^ The authors of this study suggested a longer duration of dual antiplatelet therapy as an option for these patients given the high risk of disease recurrence in this age group with lower risks of bleeding.^9^ Given that current guidelines do not account for the significant recurrence of disease in this population, the lower risk of bleeding overall in this age group and limitations of current SP medication, the longer duration of dual antiplatelets is a reasonable consideration, though further studies are required to validate the utility of this.

It is unclear why patients with pCHD should have lower rates of SP prescription. Adherence may be a factor with a multicentre study finding of those with CVD, younger patients were less adherent to medication at 30 days post discharge.^24^ A larger study of 1 248 158 US Veterans with CVD, of whom 135 703 had premature CVD, demonstrated lower odds of aspirin (OR 0.69; 95% CI 0.68 to 0.70), statin (OR 0.70; 95% CI 0.69 to 0.71) and statin adherence (OR 0.56; 95% CI 0.55 to 0.57).^18^ Similar to our findings, there was a significantly lower proportion of patients with premature CVD prescribed aspirin therapy (96 468 (71.1%) vs 860 726 (77.4%), p<0.001) and any statin therapy (98 908 (72.9%) vs 894 931(80.5%); p<0.001).^18^ Those with premature CVD were, however, more likely to be prescribed high-intensity statins, and those on high-intensity statins were more likely to be adherent to therapy.^18^

We also acknowledge that, in patients with pCHD, there may be alternative pathophysiology of CHD such as spontaneous coronary artery dissection (SCAD) or minimal obstructive coronary artery disease, where the data for SP medication are less well established, though the mainstays of SCAD therapy are beta blockers and antiplatelet therapy, which remain underprescribed in this group. This study also found that females with pCHD were even less likely to be prescribed BP lowering and antiplatelets, which is concerning.

Mahtta *et al* postulate differences in traditional RFs, alternative pathophysiology of disease and ‘therapeutic inertia’ as potential reasons for less prescription of SP therapy.^18^ Therapeutic inertia is defined as not commencing or up-titrating medication.^18^ This may contribute to explaining the reasons for our findings. There is some evidence that use of fixed dose combinations that reduce pill burden can help with therapeutic inertia^25^ and could be a suggested focus of future research. Family history of CHD is associated with an elevated risk of MI and cascade screening in relatives of patients with premature CHD has been described as a potentially effective population prevention strategy.^26 27^

### Limitations

While having strengths in being a large and relatively unselected cohort of patients with CHD from primary care, this study has limitations. The data were collected for the purpose of health service delivery and not specifically for the specific aims of the study. CHD diagnosis is based on medical histories entered by GPs and expanded aetiology or severity of the CHD is not routinely captured, though NPS does have direct processes to validate the dataset and comparisons to national datasets demonstrate the prevalence of chronic conditions, for example, MI, is aligned.^28^

In addition, in active patients, our study population, the level of prescriptions matches dispensing records.^28^ The dataset does not contain data directly from specialist care or hospitals. PCSK9 inhibitors were not placed on the Pharmaceutical Benefits Scheme (government subsidy) until 2022 which would have influenced prescribing practices. NPS does record all biochemical tests that have been incorporated into the GP databases and, therefore, does not require the GP to have ordered those tests. Therefore, there is less likely to be an underestimation of biochemical RF assessment.

## Conclusions

pCHD is an accelerated atherosclerotic process which requires intensive SP with RF modification including medical therapy. Our study demonstrates that despite no difference in clinical and biochemical RF assessment of RFs, there is an underprescription in SP medical therapy. This suggests further education, messaging and/or awareness raising is required to primary care physicians to emphasise the importance of prescribing SP medical therapy in those with pCHD. This population is at a particular high risk of recurrent events, which may have greater longer-term consequences.

## Supplementary material

10.1136/openhrt-2024-003092 online supplemental file 1

10.1136/openhrt-2024-003092 online supplemental file 2

## Data Availability

Data may be obtained from a third party and are not publicly available.

## References

[1] Shah N, Kelly A-M, Cox N (2016). Myocardial Infarction in the “Young”: Risk Factors, Presentation, Management and Prognosis. Heart Lung Circ.

[2] Bossard M, Latifi Y, Fabbri M (2020). Increasing Mortality From Premature Coronary Artery Disease in Women in the Rural United States. J Am Heart Assoc.

[3] Vikulova DN, Grubisic M, Zhao Y (2019). Premature Atherosclerotic Cardiovascular Disease: Trends in Incidence, Risk Factors, and Sex-Related Differences, 2000 to 2016. J Am Heart Assoc.

[4] Yang J, Biery DW, Singh A (2020). Risk Factors and Outcomes of Very Young Adults Who Experience Myocardial Infarction: The Partners YOUNG-MI Registry. Am J Med.

[5] Statistics ABo (2018). Changing patterns of mortality in Australia, 1968-2017. https://www.abs.gov.au/ausstats/abs@.nsf/Lookup/by%20Subject/3303.0.55.003~1968-2017~Main%20Features~Fifty%20Years%20of%20Cardiovascular%20Mortality~2.

[6] Ritchey MD, Wall HK, George MG (2020). US trends in premature heart disease mortality over the past 50 years: Where do we go from here?. Trends Cardiovasc Med.

[7] Institute. BHaD https://www.baker.edu.au/health-hub/sudden-cardiac-death.

[8] Wilmot KA, O’Flaherty M, Capewell S (2015). Coronary Heart Disease Mortality Declines in the United States From 1979 Through 2011: Evidence for Stagnation in Young Adults, Especially Women. Circulation.

[9] Zeitouni M, Clare RM, Chiswell K (2020). Risk Factor Burden and Long-Term Prognosis of Patients With Premature Coronary Artery Disease. J Am Heart Assoc.

[10] Collet J-P, Zeitouni M, Procopi N (2019). Long-Term Evolution of Premature Coronary Artery Disease. J Am Coll Cardiol.

[11] Yusuf S, Hawken S, Ôunpuu S (2004). Effect of potentially modifiable risk factors associated with myocardial infarction in 52 countries (the INTERHEART study): case-control study. *The Lancet*.

[12] Jortveit J, Halvorsen S, Kaldal A (2019). Unsatisfactory risk factor control and high rate of new cardiovascular events in patients with myocardial infarction and prior coronary artery disease. BMC Cardiovasc Disord.

[13] Visseren FLJ, Mach F, Smulders YM (2021). 2021 ESC Guidelines on cardiovascular disease prevention in clinical practice: Developed by the Task Force for cardiovascular disease prevention in clinical practice with representatives of the European Society of Cardiology and 12 medical societies With the special contribution of the European Association of Preventive Cardiology (EAPC). Eur Heart J.

[14] Busingye D, Gianacas C, Pollack A (2019). Data Resource Profile: MedicineInsight, an Australian national primary health care database. Int J Epidemiol.

[15] Arnett DK, Blumenthal RS, Albert MA (2019). 2019 ACC/AHA Guideline on the Primary Prevention of Cardiovascular Disease: A Report of the American College of Cardiology/American Heart Association Task Force on Clinical Practice Guidelines. J Am Coll Cardiol.

[16] DeFilippis EM, Singh A, Divakaran S (2018). Cocaine and Marijuana Use Among Young Adults With Myocardial Infarction. J Am Coll Cardiol.

[17] Mahtta D, Ramsey D, Krittanawong C (2021). Recreational substance use among patients with premature atherosclerotic cardiovascular disease. Heart.

[18] Mahtta D, Ramsey DJ, Al Rifai M (2020). Evaluation of Aspirin and Statin Therapy Use and Adherence in Patients With Premature Atherosclerotic Cardiovascular Disease. JAMA Netw Open.

[19] Paavola T, Bergmann U, Kuusisto S (2021). Distinct Fatty Acid Compositions of HDL Phospholipids Are Characteristic of Metabolic Syndrome and Premature Coronary Heart Disease-Family Study. Int J Mol Sci.

[20] Kazi SN, Von Huben A, Marschner S (2023). Trends in Modifiable Risk Factors Amongst First Presentation ST Elevation Myocardial Infarction Patients in a Large Longitudinal Registry. *Heart, Lung and Circulation*.

[21] Kim I, Kim MC, Sim DS (2018). Effect of the Metabolic Syndrome on Outcomes in Patients Aged <50 Years Versus >50 Years With Acute Myocardial Infarction. Am J Cardiol.

[22] Marx N, Husain M, Lehrke M (2022). GLP-1 Receptor Agonists for the Reduction of Atherosclerotic Cardiovascular Risk in Patients With Type 2 Diabetes. Circulation.

[23] Thalmann I, Preiss D, Schlackow I (2024). Quality of care for secondary cardiovascular disease prevention in 2009–2017: population-wide cohort study of antiplatelet therapy use in Scotland. *BMJ Qual Saf*.

[24] Cohen MJ, Shaykevich S, Cawthon C (2012). Predictors of medication adherence postdischarge: the impact of patient age, insurance status, and prior adherence. J Hosp Med.

[25] Wang N, Von Huben A, Marschner S (2024). Therapeutic Inertia With Initial Low-Dose Quadruple Combination Therapy for Hypertension: Results From the QUARTET Trial. Hypertension.

[26] Chow CK, Pell ACH, Walker A (2007). Families of patients with premature coronary heart disease: an obvious but neglected target for primary prevention. BMJ.

[27] Chow CK, Islam S, Bautista L (2011). Parental history and myocardial infarction risk across the world: the INTERHEART Study. J Am Coll Cardiol.

[28] Mina R, Thistlethwaite J, Belcher J (2020). MedicineInsight report: validation of the medicineInsight database: completeness, generalisability and plausibility.

