# Supplementary material for: Risk factor monitoring, management and use of prevention medicines in those with a history of premature coronary heart disease
Source: Open Heart. 2025 Sep 11;12(2):e003092. doi: 10.1136/openhrt-2024-003092 (PMC12519390; doi:10.1136/openhrt-2024-003092)
Supplement: online supplemental file 1 [file openhrt-12-2-s001.pdf]

MedicineInsight

NPS MedicineInsight

Condition Flags Dictionary

Updated: 12 September 2025

NPS MEDICINEWISE

Contents

1 Introduction ... 3

2 Condition flags in MedicineInsight ... 4

2.1 Anxiety (f_ANX) ... 4
2.2 Arthritis (f_ARTH) ... 6
2.3 Asthma (f_ASTH) ... 10
2.4 Atrial Fibrillation (f_AF) ... 12
2.5 Atrial Flutter (f_AFL) ... 13
2.6 Bipolar Disorder (f_BIPOL) ... 14
2.7 Cancer (f_CANC) ... 15
2.8 Carotid Artery Stenosis (f_CASTEN) ... 21
2.9 Chronic Kidney Disease - Stage 1 (f_CKD_1) ... 22
2.10 Chronic Kidney Disease - Stage 2 (f_CKD_2) ... 23
2.11 Chronic Kidney Disease - Stage 3 (f_CKD_3) ... 24
2.12 Chronic Kidney Disease - Stage 4 (f_CKD_4) ... 25
2.13 Chronic Kidney Disease - Stage 5 (f_CKD_5) ... 26
2.14 Chronic Kidney Disease - Unspecified (f_CKD_UNSP) ... 27
2.15 Chronic Liver Disease (f_CLD) ... 29
2.16 Chronic Obstructive Pulmonary Disease (f_COPD) ... 31
2.17 Chronic Pain (f_PAIN_CHR) ... 32
2.18 Coronary Heart Disease and Atherosclerosis (f_CHD_ATH) ... 33
2.19 Cough (f_COUGH) ... 37
2.20 Crohn's Disease (f_CROHNS) ... 39
2.21 Deep venous thrombosis (f_DVT) ... 40
2.22 Dementia (f_DEMEN) ... 41
2.23 Depression (f_DEPR) ... 43
2.24 Diabetes Mellitus Gestational (f_DM_GEST) ... 45
2.25 Diabetes Mellitus Type 1 (f_DM_T1) ... 46
2.26 Diabetes Mellitus Type 2 (f_DM_T2) ... 47
2.27 Diabetes Mellitus Type 3 (f_DM_T3) ... 48
2.28 Diabetes Mellitus Unspecified (f_DM_US) ... 49
2.29 Dyslipidaemia (f_DYS) ... 50
2.30 Epilepsy (f_EPIL) ... 51
2.31 Gastro-oesophageal Reflux Disease (f_GORD) ... 54
2.32 Heart Failure (f_HF) ... 55
2.33 Hypercholesterolaemia (f_HYPERC) ... 57
2.34 Hyperlipidaemia (f_HYPERLI) ... 58
2.35 Hypertension (f_HYPT) ... 59
2.36 Hypertriglyceridemia (f_HYPERTRIG) ... 61
2.37 Influenza (f_FLU) ... 62
2.38 Influenza-like illness (f_ILI) ... 63
2.39 Juvenile Rheumatoid Arthritis (f_ARTH_JRA) ... 64
2.40 Lower Back pain (f_PAIN_BACK_L) ... 65
2.41 Lower Respiratory Tract Infection (f_LRTI) ... 69
2.42 Osteoporosis (f_OP) ... 73
2.43 Otitis media (f_OTITIS_M) ... 74
2.44 Peripheral vascular disease (f_PVD) ... 75
2.45 Pertussis (f_PERTUSS) ... 76
2.46 Polycystic ovarian syndrome (f_PCOS) ... 77
2.47 Prostatism (f_PROSTATISM) ... 78
2.48 Pulmonary Embolism (f_PE)) ... 79
2.49 Renal Artery Stenosis (f_RASTEN) ... 80
2.50 Rheumatic Heart (f_RHEUHEAR) ... 81
2.51 Rheumatoid Arthritis (f_ARTH_RH) ... 82
2.52 Schizophrenia (f_SCHIZ) ... 83

2.53 Stroke (f_STROKE_ALL) ... 85
2.54 Stroke Haemorrhagic (f_STR_H) ... 87
2.55 Stroke Ischaemic (f_STR_I) ... 88
2.56 Stroke Lacunar (f_STR_L) ... 88
2.57 Stroke Migrainous (f_STR_M) ... 90
2.58 Stroke Thrombotic (f_STR_T) ... 91
2.59 Stroke Unspecified (f_STR_US) ... 92
2.60 Substance Abuse (f_ABU_SUB) ... 93
2.61 Transient Ischaemic Attack (f_TIA) ... 99
2.62 Ulcerative Colitis (f_COLI_ULC) ... 100

1 Introduction

Condition flags are derived fields in the MedicineInsight database. They have been developed to indicate whether a specific clinical condition is reported for a particular patient in any of the data provided to MedicineInsight.

To create a condition flag, three fields are examined - 'Diagnosis', 'Reason for visit' and 'Reason for prescription'. If the condition (or a relevant synonym) is documented in any of these fields, a condition flag is generated.

Condition flags are stored in MedicineInsight in two ways.

- The *Conditions Detail* table contains a record for every instance where a condition flag is generated. Therefore this table will contain multiple records for a patient if a condition is identified in more than one of the three examined fields, or has been recorded on more than one occasion
- The *Conditions Summary* table contains only one record for each patient. In this table, each condition flag is set to ‘1’ if the condition has ever been identified for the patient.

Example of *Conditions Detail* table:

Patient _ID	Condition Date	Text (from 'Diagnosis', 'Reason for visit' or 'Reason for prescription')	f_ANXIETY	f_ARTHRITIS	f_ASTHMA
**00001**	21/05/2015	ACUTE SEVERE ASTHMA			1
**00001**	04/02/2016	ANXIETY ATTACKS AND ASTHMA	1		1
**00001**	09/05/2016	ANXIETY – GENERALISED	1		
**00001**	10/10/2016	ASTHMA			1
**00001**	10/10/2016	REVIEW ASTHMA ACTION PLAN			1
**00002**	06/01/2015	KNEE OSTEOARTHRITIS		1	
**00002**	09/05/2015	ASTHMA			1
**00002**	11/04/2016	ARTHRITIS – OSTEO		1	
**00002**	01/11/2016	ASTHMA - EXACERBATION			1
**00002**	19/12/2016	ARTHRITIS		1	
**00003**	05/05/2015	HIP OSTEOARTHRITIS		1	

Example of *Conditions Summary* table:

Patient _ID			f_ANXIETY	f_ARTHRITIS	f_ASTHMA
**00001**			1		1
**00002**				1	1
**00003**				1	

2 Condition flags in MedicineInsight

2.1 Anxiety (f_ANX)

This flag indicates those records where Anxiety (or a relevant synonym) is reported in MedicineInsight either as a coded condition (using a drop down list in the CIS) or as a non-coded condition (free text) in one or more of the 'Diagnosis', 'Reason for visit' or 'Reason for prescription' fields.

Note: This flag reflects Anxiety broadly understood and includes:

- Anxiety
- Generalised Anxiety Disorder
- Social Anxiety Disorder

It does not include (when documented in isolation):

- Anxiety attacks
- Anxiety feeling
- Adjustment disorder
- Performance anxiety
- Anxiety-related insomnia
- Separation anxiety
- Parental anxiety
- Neurosis
- OCD (separate flag)
- PTSD (separate flag)
- Phobias (separate flag)
- Panic disorders (separate flag)

The following coded terms (from Docle or Pyefinch) have been used to identify records for inclusion:

- ADJUSTMENT DISORDER WITH ANXIETY
- ADJUSTMENT DISORDER WITH MIXED ANXIETY AND DEPRESSED MOOD
- ANXIETY
- ANXIETY - GENERALISED
- ANXIETY - PTSD
- ANXIETY - SOCIAL
- ANXIETY DISORDER
- ANXIETY DISORDER, SUBSTANCE INDUCED
- ANXIETY NEUROSIS
- ANXIETY PHOBIA
- ANXIETY WITH PANIC ATTACKS
- ANXIETY/DEPRESSION
- DEPRESSION/ANXIETY
- DEPRESSIVE ANXIETY DISORDER
- GAD
- GAD (GENERALISED ANXIETY DISORDER)
- GENERALISED ANXIETY DISORDER
- GENERALISED ANXIETY DISORDER (GAD)
- MIXED ANXIETY DEPRESSION
- MIXED ANXIETY/DEPRESSIVE DISORDER
- MIXED DEPRESSION ANXIETY

- NERVOUS ANXIETY
- NEUROTIC ANXIETY
- PHOBIC ANXIETY DISORDER
- SOCIAL ANXIETY DISORDER
- SOCIAL PHOBIA
- SUBSTANCE INDUCED ANXIETY DISORDER

The following free text strings have been used to search for terms which may indicate records for inclusion:
- ANX
- AX
- GAD

Records identified by a free text string alone are not automatically flagged, but are individually reviewed by a clinical coder to determine whether the text string actually refers to the condition indicated or is present in another context (eg, a search for 'cancer' may identify 'partner died from cancer'). Each record is flagged accordingly.

This condition flag was last updated in October 2019.

2.2 Arthritis (f_ARTH)

This flag indicates those records where ARTHRITIS (or a relevant synonym) is reported in MedicineInsight either as a coded condition (using a drop down list in the CIS) or as a non-coded condition (free text) in one or more of the 'Diagnosis', 'Reason for visit' or 'Reason for prescription' fields.

The following coded terms (from Docle or Pyefinch) have been used to identify records for inclusion:

- AC JOINT ARTHRITIS
- ACROMIOCLAVICULAR JOINT ARTHRITIS
- ANEURYSM-OSTEOARTHRITIS SYNDROME
- ANKLE OSTEOARTHRITIS
- ANKYLOSING SPONDYLITIS
- ARTHRITIS
- ARTHRITIS - GOUTY
- ARTHRITIS - JUVENILE RHEUMATOID
- ARTHRITIS - LISFRANC
- ARTHRITIS - LUPUS
- ARTHRITIS - OSTEO
- ARTHRITIS - PSORIATIC
- ARTHRITIS - RHEUMATOID
- ARTHRITIS - SEPTIC
- ARTHRITIS - SERONEGATIVE
- ARTHRITIS - VIRAL
- ARTHRITIS OF SPINE
- ARTHRITIS OF THE ACROMIOCLAVICULAR JOINT
- ARTHRITIS, INFLAMMATORY
- ARTHRITIS, JUVENILE RHEUMATOID
- ARTHRITIS, PSORIATIC
- ARTHRITIS, RHEUMATOID
- ARTHRITIS, SEPTIC
- ARTHRITIS, SERONEGATIVE
- ARTHRITIS, VIRAL
- CAPLAN SYNDROME
- CERVICAL - OSTEO ARTHRITIS
- CERVICAL SPINE OSTEOARTHRITIS
- ELBOW OSTEOARTHRITIS
- FACET JOINT ARTHRITIS
- GENERALISED OSTEOARTHRITIS
- GIANT CELL RETICULOHISTIOCYTOSIS
- GOUT
- GOUTY ARTHRITIS
- HALLUX RIGIDUS
- HIP OSTEOARTHRITIS
- HIP OSTEOARTHROSIS
- HYPERURICAEMIA
- HYPERURICEMIA
- INFLAMMATORY POLYARTHRITIS
- JOINT INFECTION
- JRA
- JRA (JUVENILE RHEUMATOID ARTHRITIS)

- JUVENILE IDIOPATHIC ARTHRITIS
- JUVENILE RHEUMATOID ARTHRITIS
- KNEE OSTEOARTHRITIS
- KNEE OSTEOARTHROSIS
- LIPOID DERMATOARTHRITIS
- LIPOID RHEUMATISM
- LISFRANC ARTHRITIS
- LOEYS-DIETZ SYNDROME TYPE 3
- LUMBAR - OSTEO ARTHRITIS
- LUMBAR SPINE OSTEOARTHRITIS
- LUPUS ARTHRITIS
- LYME ARTHRITIS
- MIDFOOT OSTEOARTHRITIS
- MONOARTHRITIS
- MULTICENTRIC RETICULOHISTIOCYTOSIS
- OA
- OA (OSTEOARTHRITIS)
- OLIGOARTHRITIS, INFLAMMATORY
- OSTEOARTHRITIS
- OSTEOARTHRITIS - ANKLE
- OSTEOARTHRITIS - ELBOW
- OSTEOARTHRITIS - FINGERS
- OSTEOARTHRITIS - GLENOHUMERAL JOINT
- OSTEOARTHRITIS - HANDS
- OSTEOARTHRITIS - HIP
- OSTEOARTHRITIS - KNEE
- OSTEOARTHRITIS - NECK
- OSTEOARTHRITIS - SHOULDER
- OSTEOARTHRITIS - SPINE
- OSTEOARTHRITIS OF 1ST CARPOMETACARPAL JOINT
- OSTEOARTHRITIS OF 1ST CARPO-METACARPAL JOINT
- OSTEOARTHRITIS OF 1ST METATARSOPHALANGEAL JOINT
- OSTEOARTHRITIS OF ANKLE
- OSTEOARTHRITIS OF CERVICAL SPINE
- OSTEOARTHRITIS OF ELBOW
- OSTEOARTHRITIS OF FINGERS
- OSTEOARTHRITIS OF FOOT
- OSTEOARTHRITIS OF HAND
- OSTEOARTHRITIS OF HIP
- OSTEOARTHRITIS OF KNEE
- OSTEOARTHRITIS OF LUMBAR SPINE
- OSTEOARTHRITIS OF NECK
- OSTEOARTHRITIS OF SACROILIAC JOINTS
- OSTEOARTHRITIS OF SHOULDER
- OSTEOARTHRITIS OF THE PATELLOFEMORAL JOINT
- OSTEOARTHRITIS OF THORACIC SPINE
- OSTEOARTHRITIS OF TMJ
- OSTEOARTHRITIS OF WRIST
- OSTEOARTHRITIS, GENERALISED
- OSTEOARTHROSIS
- PATELLOFEMORAL OSTEOARTHRITIS
- PODAGRA

- POLYARTHRITIS
- POLYARTHRITIS, INFLAMMATORY
- PSORIATIC ARTHRITIS
- PSORIATIC ARTHROPATHY
- RA
- RA (RHEUMATOID ARTHRITIS)
- REACTIVE ARTHRITIS
- REITER'S DISEASE
- REITER'S SYNDROME
- RHEUMATOID ARTHRITIS
- RHEUMATOID ARTHRITIS - JUVENILE
- RHEUMATOID ARTHRITIS - PNEUMOCONIOSIS
- RHEUMATOID ARTHRITIS, JUVENILE
- SACROILIAC JOINT ARTHRITIS
- SEPTIC ARTHRITIS
- SERONEGATIVE ARTHRITIS
- SERONEGATIVE RHEUMATOID ARTHRITIS
- SHOULDER OSTEOARTHRITIS
- SPONDYLOARTHRITIS
- SPONDYLOSIS
- STILLS DISEASE
- THORACIC - OSTEO ARTHRITIS
- URATE CRYSTAL DEPOSITION
- VENEREAL ARTHRITIS
- VIRAL ARTHRITIS
- WAELSCH'S SYNDROME
- WEAR AND TEAR ARTHRITIS
- WRIST OSTEOARTHRITIS

The following free text strings have been used to search for terms which may indicate records for inclusion:

- ANKYLOSING
- ARTHRI
- CAPLAN SYNDROME
- GIANT CELL RETICULOHISTIOCYTOSIS
- GOUT
- HALLUX RIGIDUS
- HYPERURICAEMIA
- HYPERURICEMIA
- JOINT INFECTION
- LIPOID RHEUMATISM
- LOEYS-DIETZ SYNDROME TYPE 3
- LYME DISEASE
- MULTICENTRIC RETICULOHISTIOCYTOSIS
- O.A
- O/A
- OA
- PSORIATIC ARTHROPATHY
- RA
- REITER'S DISEASE
- REITER'S SYNDROME
- SPONDYLOSIS

- STILLS DISEASE
- WAELSCH'S SYNDROME

Records identified by a free text string alone are not automatically flagged, but are individually reviewed by a clinical coder to determine whether the text string actually refers to the condition indicated or is present in another context (eg, a search for 'cancer' may identify ‘partner died from cancer’). Each record is flagged accordingly.

This condition flag was last updated in January 2019.

2.3 Asthma (f_ASTH)

This flag indicates those records where Asthma (or a relevant synonym) is reported in MedicineInsight either as a coded condition (using a drop down list in the CIS) or as a non-coded condition (free text) in one or more of the 'Diagnosis', 'Reason for visit' or 'Reason for prescription' fields.

The following coded terms (from Docle or Pyefinch) have been used to identify records for inclusion:
- ACUTE SEVERE ASTHMA
- ALLERGIC ASTHMA
- ALLERGY INDUCED ASTHMA
- ASPIRIN SENSITIVE ASTHMA
- ASTHMA
- ASTHMA - ALLERGY INDUCED
- ASTHMA - CHRONIC PERSISTENT
- ASTHMA - EXERCISE INDUCED
- ASTHMA - FREQUENT EPISODIC
- ASTHMA - INFECTIVE EXACERBATION
- ASTHMA - INFREQUENT EPISODIC
- ASTHMA - PRECIPITATED BY BACTERIAL INFECTION
- ASTHMA - PRECIPITATED BY VIRAL INFECTION
- ASTHMA ACTION PLAN
- ASTHMA ACTION PLAN PERFORMED
- ASTHMA ACTION PLAN PRINTED
- ASTHMA CARE PLAN
- ASTHMA CARE PLAN REVIEW
- ASTHMA CYCLE OF CARE
- ASTHMA EXACERBATION
- ASTHMA REVIEW
- ASTHMA, ALLERGIC
- ASTHMA, ALLERGY INDUCED
- ASTHMA, CHILDHOOD
- ASTHMA, EXERCISE INDUCED
- ASTHMA, FREQUENT EPISODIC
- ASTHMA, INFECTIVE EXACERBATION
- ASTHMA, INFREQUENT EPISODIC
- ASTHMA, OCCUPATIONAL
- ASTHMA, THUNDERSTORM
- BRONCHIAL ASTHMA
- CARE PLAN, ASTHMA
- CHECK UP, ASTHMA
- EXERCISE INDUCED ASTHMA
- EXERTIONAL ASTHMA
- FREQUENT EPISODIC ASTHMA
- INFECTIVE EXACERBATION OF ASTHMA
- INFREQUENT EPISODIC ASTHMA
- OCCUPATIONAL ASTHMA
- REVIEW - ASTHMA
- SAMTER'S TRIAD
- STATUS ASTHMATICUS
- THUNDERSTORM ASTHMA

- WHEEZY BRONCHITIS

The following text strings have been used to search for terms which may indicate records for inclusion:
- ASTH
- ASTMA
- SAMTER'S TRIAD
- SAMTER

Records identified by a free text string alone are not automatically flagged, but are individually reviewed by a clinical coder to determine whether the text string actually refers to the condition indicated or is present in another context (eg, a search for 'cancer' may identify ‘partner died from cancer’). Each record is flagged accordingly.

Note: Although ‘Wheezy bronchitis’ is not the most correct term at this time, it shares a code with Asthma and Bronchial Asthma and so has been included. It will however not be searched for as a free text term.

The conditions in this list were approved by NPS MedicineWise Medical Advisors in January 2019.

This condition flag was last updated on 01 January 2019.

2.4 Atrial Fibrillation (f_AF)

This flag indicates those records where Atrial Fibrillation (or a relevant synonym) is reported in MedicineInsight either as a coded condition (using a drop down list in the CIS) or as a non-coded condition (free text) in one or more of the 'Diagnosis', 'Reason for visit' or 'Reason for prescription' fields.

The following coded terms (from Docle or Pyefinch) have been used to identify records for inclusion:
- AF
- AF (ATRIAL FIBRILLATION)
- ARRHYTHMIA, ATRIAL FIBRILLATION
- ATRIAL FIBRILLATION
- ATRIAL FIBRILLATION - ISOLATED EPISODE
- ATRIAL FIBRILLATION - PAROXYSMAL
- ATRIAL FIBRILLATION ABLATION
- ATRIAL FIBRILLATION, NON-VALVULAR
- ATRIAL FIBRILLATION, VALVULAR
- FIBRILLATION - ATRIAL
- FIBRILLATION ATRIUM - PAROXYSMAL
- FIBRILLATION, ATRIAL
- NON-VALVULAR ATRIAL FIBRILLATION
- PAROXYSMAL ATRIAL FIBRILLATION
- RAPID AF
- RAPID ATRIAL FIBRILLATION
- VALVULAR ATRIAL FIBRILLATION

The following free text strings have been used to search for terms which may indicate records for inclusion:
- A FIB
- A.F
- A/F
- AF
- ATRIAL F
- FIBRILLATION

Records identified by a free text string alone are not automatically flagged, but are individually reviewed by a clinical coder to determine whether the text string actually refers to the condition indicated or is present in another context (eg, a search for 'cancer' may identify ‘partner died from cancer’). Each record is flagged accordingly.

This condition flag was last updated in August 2018.

2.5 Atrial Flutter (f_AFL)

This flag indicates those records where Atrial Flutter (or a relevant synonym) is reported in MedicineInsight either as a coded condition (using a drop down list in the CIS) or as a non-coded condition (free text) in one or more of the 'Diagnosis', 'Reason for visit' or 'Reason for prescription' fields.

The following coded terms (from Docle or Pyefinch) have been used to identify records for inclusion:
- ARRHYTHMIA, ATRIAL FLUTTER
- ATRIAL FLUTTER
- ATRIAL FLUTTER ABLATION
- FLUTTER - ATRIAL
- UNCONTROLLED ATRIAL FLUTTER

The following free text strings have been used to search for terms which may indicate records for inclusion:
- FLUTTER

Records identified by a free text string alone are not automatically flagged, but are individually reviewed by a clinical coder to determine whether the text string actually refers to the condition indicated or is present in another context (eg, a search for 'cancer' may identify 'partner died from cancer'). Each record is flagged accordingly.

This condition flag was last updated in August 2018.

2.6 Bipolar Disorder (f_BIPOL)

This flag indicates those records where BIPOLAR DISORDER (or a relevant synonym) is reported in MedicineInsight either as a coded condition (selected from a list in the CIS) or as a free text entry in one or more of the 'Diagnosis', 'Reason for visit' or 'Reason for prescription' fields.

The following coded terms (from Docle or Pyefinch) have been used to identify records for inclusion:
- BIPOLAR 1 DISORDER
- BIPOLAR 2 DISORDER
- BIPOLAR AFFECTIVE DISORDER
- BIPOLAR SPECTRUM DISORDER
- MANIC DEPRESSIVE ILLNESS
- MANIC DEPRESSIVE PSYCHOSIS

The following free text strings have been used to search for terms which may indicate records for inclusion:
- BIPOLAR
- MANIC DEPRESSIVE

Records identified by a free text string alone are not automatically flagged, but are individually reviewed by a clinical coder to determine whether the text string actually refers to the condition indicated or is present in another context (eg, a search for 'cancer' may identify 'partner died from cancer'). Each record is flagged accordingly.

This condition flag was last updated in November 2018.

2.7 Cancer (f_CANC)

This flag indicates those records where Cancer* (or a relevant synonym) is reported in MedicineInsight either as a coded condition (using a drop down list in the CIS) or as a non-coded condition (free text) in one or more of the 'Diagnosis', 'Reason for visit' or 'Reason for prescription' fields.

*Note: Excludes Skin cancer and Melanoma. These have been flagged separately.

The following coded terms (from Docle or Pyefinch) have been used to identify records for inclusion:

- ACUTE GRANULOCYTIC LEUKAEMIA
- ACUTE LYMPHOBLASTIC LEUKAEMIA
- ACUTE LYMPHOCYTIC LEUKAEMIA
- ACUTE MYELOCYTIC LEUKAEMIA
- ACUTE MYELOID LEUKAEMIA
- ADAMANTINOMA
- ADENOCARCINOMA - AMPULLA OF VATER
- ADENOCARCINOMA - BREAST
- ADENOCARCINOMA - COLON
- ADENOCARCINOMA - ENDOMETRIUM
- ADENOCARCINOMA - GALLBLADDER
- ADENOCARCINOMA - LUNG
- ADENOCARCINOMA - PANCREAS
- ADENOCARCINOMA - PROSTATE
- ADENOCARCINOMA - SMALL BOWEL
- ADENOCARCINOMA - STOMACH
- ADENOCARCINOMA - UNKNOWN PRIMARY
- ADENOCARCINOMA - UTERINE
- ADENOCARCINOMA OR CARCINOMA - NONSPECIFIC
- ADENOID CYSTIC CARCINOMA
- ADRENAL CARCINOMA
- ALL (ACUTE LYMPHOCYTIC LEUKAEMIA)
- AMELOBLASTOMA
- AML (ACUTE MYELOCYTIC LEUKAEMIA)
- AMPULLARY ADENOCARCINOMA
- AMPULLARY CARCINOMA
- ASTROCYTOMA
- BILIARY CARCINOMA
- BLADDER CANCER
- BOWEL CANCER
- BRAIN CARCINOMA
- BREAST CANCER
- BREAST CANCER - MALE
- BREAST CARCINOMA
- BRONCHOGENIC CARCINOMA
- CAECAL CARCINOMA
- CANCER - UNKNOWN PRIMARY
- CANCER OF OVARY
- CANCER OF PANCREAS
- CARCINOMA - AMPULLA OF VATER
- CARCINOMA - BILE DUCT

- CARCINOMA - BLADDER
- CARCINOMA - BRAIN
- CARCINOMA - BREAST
- CARCINOMA - BREAST - MALE
- CARCINOMA - CAECAL
- CARCINOMA - CERVIX
- CARCINOMA - CHORION
- CARCINOMA - COLON
- CARCINOMA - GALLBLADDER
- CARCINOMA - HYPOTHALAMUS
- CARCINOMA - KIDNEY
- CARCINOMA - LARYNX
- CARCINOMA - LIVER
- CARCINOMA - LIVER - FIBROLAMELLAR
- CARCINOMA - LUNG
- CARCINOMA - LUNG - ADENOCARCINOMA
- CARCINOMA - LUNG - ALVEOLAR CELL
- CARCINOMA - LUNG - LARGE CELL
- CARCINOMA - LUNG - SMALL CELL
- CARCINOMA - LYMPH VESSEL
- CARCINOMA - MOUTH
- CARCINOMA - NASOPHARYNX
- CARCINOMA - OESOPHAGUS
- CARCINOMA - OROPHARYNX
- CARCINOMA - OVARY
- CARCINOMA - PANCREAS
- CARCINOMA - PAROTID
- CARCINOMA - PAROTID - MIXED
- CARCINOMA - PITUITARY
- CARCINOMA - PROSTATE
- CARCINOMA - RATHKE'S POUCH
- CARCINOMA - RECTUM
- CARCINOMA - RETINA
- CARCINOMA - SALIVARY GLAND
- CARCINOMA - STOMACH
- CARCINOMA - TESTIS
- CARCINOMA - THYROID
- CARCINOMA - TONGUE
- CARCINOMA - TONSIL
- CARCINOMA - UTERUS
- CARCINOMA - VAGINA
- CARCINOMA - VULVA
- CARCINOMA OR ADENOCARCINOMA - NONSPECIFIC
- CERVICAL CANCER
- CERVICAL CARCINOMA
- CHOLANGIOCARCINOMA
- CHORIOCARCINOMA
- CHRONIC LYMPHATIC LEUKAEMIA
- CHRONIC LYMPHOCYTIC LEUKAEMIA
- CLL (CHRONIC LYMPHOCYTIC LEUKAEMIA)
- COLON - ADENOCARCINOMA
- COLONIC CANCER

- COLONIC CARCINOMA
- COMMON BILE DUCT CANCER
- CRANIOPHARYNGIOMA
- ENDOMETRIAL ADENOCARCINOMA
- GALLBLADDER - CANCER
- GALLBLADDER - CARCINOMA
- GASTRIC ADENOCARCINOMA
- GASTRIC CANCER
- GASTRIC CARCINOMA
- GESTATIONAL CHORIOCARCINOMA
- GLIOBLASTOMA MULTIFORME
- GLIOMA
- HEPATOCELLULAR CARCINOMA
- HEPATOMA
- HEREDITARY NON-POLYPOSIS COLON CANCER
- HISTIOCYTOMA
- HNPCC (HEREDITARY NON-POLYPOSIS COLON CANCER)
- HYPERNEPHROMA
- HYPOTHALAMIC CARCINOMA
- KIDNEY CANCER
- LARYNGEAL CARCINOMA
- LEUKAEMIA
- LEUKAEMIA - ACUTE LYMPHOCYTIC
- LEUKAEMIA - ACUTE MYELOID
- LEUKAEMIA - CHRONIC LYMPHOCYTIC
- LINITUS PLASTICA
- LOBULAR CARCINOMA IN-SITU
- LUNG ADENOCARCINOMA
- LUNG CANCER
- LYMPHANGITIS CARCINOMATOSA
- LYMPHANGITIS CARCINOMATOSIS
- MALIGNANT GLIOMA
- MAMMARY CARCINOMA IN THE MALE
- MEDULLARY THYROID CARCINOMA
- MIXED PAROTID TUMOR
- NASOPHARYNGEAL CARCINOMA
- NASOPHARYNGEAL LYMPHOEPITHELIOMA
- NEPHRECTOMY - CANCER
- NEPHRECTOMY - TUMOR
- NODULAR THYROID CARCINOMA
- OESOPHAGEAL ADENOCARCINOMA
- OESOPHAGEAL CARCINOMA
- OLIGODENDROGLIOMA
- OROPHARYNGEAL CARCINOMA
- OVARIAN ADENOCARCINOMA
- OVARIAN CANCER
- OVARIAN CARCINOMA
- PANCREATIC ADENOCARCINOMA
- PANCREATIC CANCER
- PANCREATIC CARCINOMA
- PAPILLARY THYROID CARCINOMA
- PAROTID ADENOCARCINOMA

- PAROTID ADENOLYMPHOMA
- PAROTID CARCINOMA
- PERI-AMPULLARY CARCINOMA
- PHOBIA - BREAST CANCER
- PHOBIA - CANCER
- POST NASAL CANCER
- PRIMARY UNKNOWN ADINOCARCINOMA
- PROSTATE CANCER
- PROSTATIC ADENOCARCINOMA
- RECTAL ADENOCARCINOMA
- RECTAL CANCER
- RECTAL CARCINOMA
- RENAL CARCINOMA
- RETINAL CANCER
- SMALL BOWEL ADENOCARCINOMA
- SPINDLE CELL CANCER
- STOMACH CANCER
- TESTICULAR CARCINOMA
- THYROID CANCER
- THYROID CARCINOMA
- TONGUE CARCINOMA
- TONSILLAR CARCINOMA
- TONSILLAR LYMPHOMA
- UNDIFFERENTIATED THYROID CANCER
- UNDIFFERENTIATED THYROID CARCINOMA
- UNKNOWN PRIMARY ADINOCARCINOMA
- URINARY BLADDER CARCINOMA
- UTERINE ADENOCARCINOMA
- UTERINE CANCER
- VAGINAL CARCINOMA
- VAGINAL MALIGNANCY
- VESICAL ADENOCARCINOMA
- VULVAL CARCINOMA

The following free text strings have been used to search for terms which may indicate records for inclusion:

- ADAMANTINOMA
- ADULT WILMS TUMOUR
- ALL
- AML
- ASTROCYTOMA
- BENIGN MESENCHYMAL MELANOMA
- BLASTOMA
- CANCER
- CARCINOMA
- CHROMATOPHOROMA
- CLL
- CML
- COLONOSCOPY
- CRANIOPHARYNGIOMA
- CUTANEOUS MASTOCYTOSIS
- CYLINDROMA

- CYSTIC MESENCHYMAL HAMARTOMA
- DEGOS DISEASE
- DERMAL MELANOCYTOMA
- DURAL ENDOTHELIOMA
- DYSMYELOPOIETIC SYNDROME
- GLIOMA
- GLOMANGIOMA
- GLOMOVENOUS MALFORMATION
- GLOMUS TUMOUR
- HEPATOMA
- HIPPEL DISEASE
- HISTIOCYTOMA
- HODGKIN'S DISEASE
- HURTHLE CELL TUMOUR
- HYPER-EOSINOPHILIC SYNDROME
- JADASSOHN-TIÈCHE NAEVUS
- JADASSOHN-TIÈCHE SYNDROME
- KLATSKIN TUMOR
- LEUKAEMIA
- LINITIS PLASTICA
- LINITUS PLASTICA
- LYMPHOMA
- LYNCH SYNDROME
- MALIGNANT ATROPHIC PAPULOSIS
- MASTOCYTOMA
- MASTOCYTOSIS, CUTANEOUS
- MELANOFIBROMA
- MENINGIOMA
- MESENCHYMAL CYSTIC HAMARTOMA
- MESOTHELIOMA
- METASTASES
- METASTASIS
- METASTATIC LESION
- METASTATIC SPREAD
- MIXED MULLERIAN TUMOUR
- MIXED PAROTID TUMOR
- MYELODYSPLASIA
- MYELO-DYSPLASTIC SYNDROME
- NASOPHARYNGEAL LYMPHOEPITHELIOMA
- NEPHRECTOMY - TUMOR
- NEPHROMA
- ODONTOGENIC TUMOUR
- ONCOCYTOMA
- OPHTHALMIC NEOPLASM
- PHAEOCHROMOCYTOMA
- PHEOCHROMOCYTOMA
- PSEUDOMYXOMA PERITONEI
- RETINAL MELANOMA
- RETINOCEREBELLAR ANGIOMATOSIS
- RODENT ULCER
- SARCOMA
- SECONDARY NEOPLASM

- SOLITARY RETICULOHISTIOCYTOSIS
- STEWART–TREVES SYNDROME
- TUMOUR CELL INFILTRATION OF BONE MARROW
- URTICARIA PIGMENTOSA
- VAGINAL MALIGNANCY
- VON HIPPEL LINDAU DISEASE
- WILMS' TUMOUR
- WILMS TUMOUR, ADULT

Records identified by a free text string alone are not automatically flagged, but are individually reviewed by a clinical coder to determine whether the text string actually refers to the condition indicated or is present in another context (eg, a search for 'cancer' may identify 'partner died from cancer'). Each record is flagged accordingly.

This condition flag was last updated in December 2018.

2.8 Carotid Artery Stenosis (f_CASTEN)

This flag indicates those records where Coronary Heart Disease (or a relevant synonym) is reported in MedicineInsight either as a coded condition (using a drop down list in the CIS) or as a non-coded condition (free text) in one or more of the 'Diagnosis', 'Reason for visit' or 'Reason for prescription' fields.

The following coded terms (from Docle or Pyefinch) have been used to identify records for inclusion:
- BLOCKED CAROTID ARTERY STENT
- CAROTID ARTERY DISSECTION
- CAROTID ARTERY OCCLUSION
- CAROTID ARTERY STENOSIS
- CAROTID ARTERY STENT
- CAROTID ARTERY STENT BLOCKED
- CAROTID ARTERY STENT OCCLUSION
- CAROTID STENOSIS
- DISSECTION OF CAROTID ARTERY
- OBSTRUCTED CAROTID ARTERY STENT
- OCCLUDED CAROTID ARTERY STENT
- STENT, CAROTID ARTERY

The following free text strings have been used to search for terms which may indicate records for inclusion:
- CAROTID ARTERY DISSECTION
- CAROTID ARTERY OCCLUSION
- CAROTID ARTERY STENOSIS
- CAROTID ARTERY STENT
- CAROTID STENOSIS
- DISSECTION OF CAROTID ARTERY
- STENT, CAROTID ARTERY

Records identified by a free text string alone are not automatically flagged, but are individually reviewed by a clinical coder to determine whether the text string actually refers to the condition indicated or is present in another context (eg, a search for 'cancer' may identify 'partner died from cancer'). Each record is flagged accordingly.

Note: The conditions in this list were approved by NPS MedicineWise Medical Advisors in January 2019.

This condition flag was last updated in January 2019.

2.9 Chronic Kidney Disease - Stage 1 (f_CKD_1)

This flag indicates those records where Chronic Kidney Disease - Stage 1 (or a relevant synonym) is reported in MedicineInsight either as a coded condition (using a drop down list in the CIS) or as a non-coded condition (free text) in one or more of the 'Diagnosis', 'Reason for visit' or 'Reason for prescription' fields.

The following coded terms (from Docle or Pyefinch) have been used to identify records for inclusion:
- CHRONIC KIDNEY DISEASE - STAGE 1
- CHRONIC KIDNEY DISEASE, STAGE 1
- CKD (CHRONIC KIDNEY DISEASE) STAGE 1
- KIDNEY DISEASE - CHRONIC - STAGE 1
- RENAL DISEASE - CHRONIC - STAGE 1

The following free text strings have been used to search for terms which may indicate records for inclusion:
- KIDNEY DISEASE - CHRONIC - STAGE 1
- RENAL DISEASE - CHRONIC - STAGE 1

Records identified by a free text string alone are not automatically flagged, but are individually reviewed by a clinical coder to determine whether the text string actually refers to the condition indicated or is present in another context (eg, a search for 'cancer' may identify 'partner died from cancer'). Each record is flagged accordingly.

Note: The conditions in this list were approved by NPS MedicineWise Medical Advisors in January 2019.

This condition flag was last updated in January 2019.

2.10 Chronic Kidney Disease - Stage 2 (f_CKD_2)

This flag indicates those records where Chronic Kidney Disease - Stage 2 (or a relevant synonym) is reported in MedicineInsight either as a coded condition (using a drop down list in the CIS) or as a non-coded condition (free text) in one or more of the 'Diagnosis', 'Reason for visit' or 'Reason for prescription' fields.

The following coded terms (from Docle or Pyefinch) have been used to identify records for inclusion:
- CHRONIC KIDNEY DISEASE - STAGE 2
- CHRONIC KIDNEY DISEASE, STAGE 2
- CKD (CHRONIC KIDNEY DISEASE) STAGE 2
- KIDNEY DISEASE - CHRONIC - STAGE 2
- RENAL DISEASE - CHRONIC - STAGE 2

The following free text strings have been used to search for terms which may indicate records for inclusion:
- KIDNEY DISEASE - CHRONIC - STAGE 2
- RENAL DISEASE - CHRONIC - STAGE 2

Records identified by a free text string alone are not automatically flagged, but are individually reviewed by a clinical coder to determine whether the text string actually refers to the condition indicated or is present in another context (eg, a search for 'cancer' may identify 'partner died from cancer'). Each record is flagged accordingly.

Note: The conditions in this list were approved by NPS MedicineWise Medical Advisors in January 2019.

This condition flag last updated in January 2019.

2.11 Chronic Kidney Disease - Stage 3 (f_CKD_3)

This flag indicates those records where Chronic Kidney Disease - Stage 3 (or a relevant synonym) is reported in MedicineInsight either as a coded condition (using a drop down list in the CIS) or as a non-coded condition (free text) in one or more of the 'Diagnosis', 'Reason for visit' or 'Reason for prescription' fields.

The following coded terms (from Docle or Pyefinch) have been used to identify records for inclusion:
- CHRONIC KIDNEY DISEASE - STAGE 3
- CHRONIC KIDNEY DISEASE, STAGE 3A
- CHRONIC KIDNEY DISEASE, STAGE 3B
- CKD (CHRONIC KIDNEY DISEASE) STAGE 3
- KIDNEY DISEASE - CHRONIC - STAGE 3
- RENAL DISEASE - CHRONIC - STAGE 3

The following free text strings have been used to search for terms which may indicate records for inclusion:
- KIDNEY DISEASE - CHRONIC - STAGE 3
- RENAL DISEASE - CHRONIC - STAGE 3

Records identified by a free text string alone are not automatically flagged, but are individually reviewed by a clinical coder to determine whether the text string actually refers to the condition indicated or is present in another context (eg, a search for 'cancer' may identify 'partner died from cancer'). Each record is flagged accordingly.

Note: The conditions in this list were approved by NPS MedicineWise Medical Advisors in January 2019.

This condition flag was last updated in January 2019.

2.12 Chronic Kidney Disease - Stage 4 (f_CKD_4)

This flag indicates those records where Chronic Kidney Disease - Stage 4 (or a relevant synonym) is reported in MedicineInsight either as a coded condition (using a drop down list in the CIS) or as a non-coded condition (free text) in one or more of the 'Diagnosis', 'Reason for visit' or 'Reason for prescription' fields.

The following coded terms (from Docle or Pyefinch) have been used to identify records for inclusion:
- CHRONIC KIDNEY DISEASE - STAGE 4
- CHRONIC KIDNEY DISEASE, STAGE 4
- CKD (CHRONIC KIDNEY DISEASE) STAGE 4
- KIDNEY DISEASE - CHRONIC - STAGE 4
- RENAL DISEASE - CHRONIC - STAGE 4

The following free text strings have been used to search for terms which may indicate records for inclusion:
- KIDNEY DISEASE - CHRONIC - STAGE 4
- RENAL DISEASE - CHRONIC - STAGE 4

Records identified by a free text string alone are not automatically flagged, but are individually reviewed by a clinical coder to determine whether the text string actually refers to the condition indicated or is present in another context (eg, a search for 'cancer' may identify 'partner died from cancer'). Each record is flagged accordingly.

Note: The conditions in this list were approved by NPS MedicineWise Medical Advisors in January 2019.

This condition flag was last updated in January 2019.

2.13 Chronic Kidney Disease - Stage 5 (f_CKD_5)

This flag indicates those records where Chronic Kidney Disease - Stage 5 (or a relevant synonym) is reported in MedicineInsight either as a coded condition (using a drop down list in the CIS) or as a non-coded condition (free text) in one or more of the 'Diagnosis', 'Reason for visit' or 'Reason for prescription' fields.

The following coded terms (from Docle or Pyefinch) have been used to identify records for inclusion:

- CHRONIC KIDNEY DISEASE - STAGE 5
- CHRONIC KIDNEY DISEASE, STAGE 5
- CKD (CHRONIC KIDNEY DISEASE) STAGE 5
- KIDNEY DISEASE - CHRONIC - STAGE 5
- RENAL DISEASE - CHRONIC - STAGE 5

The following free text strings have been used to search for terms which may indicate records for inclusion:

- KIDNEY DISEASE - CHRONIC - STAGE 5
- RENAL DISEASE - CHRONIC - STAGE 5

Records identified by a free text string alone are not automatically flagged, but are individually reviewed by a clinical coder to determine whether the text string actually refers to the condition indicated or is present in another context (eg, a search for 'cancer' may identify 'partner died from cancer'). Each record is flagged accordingly.

Note: The conditions in this list were approved by NPS MedicineWise Medical Advisors in January 2019.

This condition flag was last updated in January 2019.

2.14 Chronic Kidney Disease - Unspecified (f_CKD_UNSP)

This flag indicates those records where Chronic Kidney Disease – Stage unspecified (or a relevant synonym) is reported in MedicineInsight either as a coded condition (using a drop down list in the CIS) or as a non-coded condition (free text) in one or more of the 'Diagnosis', 'Reason for visit' or 'Reason for prescription' fields.

The following coded terms (from Docle or Pyefinch) have been used to identify records for inclusion:
- ANAEMIA - CHRONIC RENAL FAILURE
- CAPD
- CAPD (CONTINUOUS AMBULATORY PERITONEAL DIALYSIS)
- CATHETERISATION - PERITONEUM
- CATHETERISATION OF PERITONEUM
- CHRONIC KIDNEY DISEASE
- CHRONIC RENAL FAILURE
- CHRONIC RENAL FAILURE - HYPERPARATHYROIDISM
- CONTINUOUS AMBULATORY PERITONEAL DIALYSIS
- DIALYSIS
- DIALYSIS - HAEMODIALYSIS
- DIALYSIS - PERITONEAL
- DIALYSIS, PERITONEAL
- HAEMODIALYSIS
- HEMODIALYSIS
- KIDNEY FAILURE - CHRONIC
- KIDNEY FAILURE, CHRONIC
- PERITONEAL CATHERISATION FOR DIALYSIS
- PERITONEAL CATHETERISATION FOR DIALYSIS
- PERITONEAL DIALYSIS
- RENAL DIALYSIS
- RENAL FAILURE, CHRONIC
- RENAL INSUFFICIENCY - CHRONIC
- SURGERY - ABDOMEN - DIALYSIS - CATHETERISATION

The following free text strings have been used to search for terms which may indicate records for inclusion:
- CAPD
- CHRONIC KIDNEY DISEASE
- CKD
- CRF
- DIALYSIS
- KIDNEY FAILURE
- RENAL FAILURE
- RENAL IMPAIRMENT
- RENAL INSUFFICIENCY - CHRONIC

Records identified by a free text string alone are not automatically flagged, but are individually reviewed by a clinical coder to determine whether the text string actually refers to the condition indicated or is present in another context (eg, a search for 'cancer' may identify 'partner died from cancer'). Each record is flagged accordingly.

Note: The conditions in this list were approved by NPS MedicineWise Medical Advisors in January 2019.

This condition flag was last updated in January 2019.

2.15 Chronic Liver Disease (f_CLD)

This flag indicates those records where Chronic Liver Disease (or a relevant synonym) is reported in MedicineInsight either as a coded condition (using a drop down list in the CIS) or as a non-coded condition (free text) in one or more of the 'Diagnosis', 'Reason for visit' or 'Reason for prescription' fields.

The following coded terms (from Docle or Pyefinch) have been used to identify records for inclusion:
- CIRRHOSIS
- CIRRHOSIS - ALPHA1 ANTITRYPSIN DEFICIENCY
- CIRRHOSIS OF THE LIVER
- CIRRHOSIS WITH ACUTE RENAL FAILURE
- COMA - HEPATIC
- COPPER STORAGE DISEASE
- ENCEPHALOPATHY - HEPATIC
- ENCEPHALOPATHY LIVER FAILURE
- FAILURE - LIVER
- FIBROSIS OF LIVER
- HEPATIC CIRRHOSIS
- HEPATIC COMA
- HEPATIC FAILURE
- HEPATIC FIBROSIS
- HEPATIC PRE-COMA
- HEPATOLENTICULAR DEGENERATION
- HEPATORENAL SYNDROME
- LIVER CIRRHOSIS
- LIVER FAILURE
- LIVER FAILURE - ENCEPHALOPATHY
- LIVER FIBROSIS
- RENAL FAILURE DUE TO CIRRHOSIS
- WILSON'S DEGENERATION
- WILSON'S DISEASE
- WILSON'S SYNDROME

The following free text strings have been used to search for terms which may indicate records for inclusion:
- CIRRHOSIS
- COMA - HEPATIC
- COPPER STORAGE DISEASE
- ENCEPHALOPATHY - HEPATIC
- FAILURE - LIVER
- FIBROSIS OF LIVER
- HEPATIC COMA
- HEPATIC FAILURE
- HEPATIC FIBROSIS
- HEPATIC PRE-COMA
- HEPATOLENTICULAR DEGENERATION
- HEPATORENAL SYNDROME
- LIVER FAILURE
- LIVER FIBROSIS
- WILSON'S DEGENERATION

- WILSON'S DISEASE
- WILSON'S SYNDROME

Records identified by a free text string alone are not automatically flagged, but are individually reviewed by a clinical coder to determine whether the text string actually refers to the condition indicated or is present in another context (eg, a search for 'cancer' may identify 'partner died from cancer'). Each record is flagged accordingly.

This condition flag was last updated in August 2018.

2.16 Chronic Obstructive Pulmonary Disease (f_COPD)

This flag indicates those records where Chronic Obstructive Pulmonary Disease (or a relevant synonym) is reported in MedicineInsight either as a coded condition (using a drop down list in the CIS) or as a non-coded condition (free text) in one or more of the 'Diagnosis', 'Reason for visit' or 'Reason for prescription' fields.

The following coded terms (from Docle or Pyefinch) have been used to identify records for inclusion:
- ACUTE EXACERBATION OF COPD
- BRONCHITIS - CHRONIC
- BRONCHITIS, CHRONIC
- CAL (CHRONIC AIRWAYS LIMITATION)
- CHRONIC AIRWAYS LIMITATION
- CHRONIC BRONCHITIS
- CHRONIC BRONCHITIS - INFECTIVE EXACERBATION
- CHRONIC BRONCHITIS, INFECTIVE EXACERBATION
- CHRONIC OBSTRUCTIVE AIRWAYS DISEASE
- CHRONIC OBSTRUCTIVE PULMONARY DISEASE
- COAD
- COAD - INFECTIVE EXACERBATION
- COAD (CHRONIC OBSTRUCTIVE AIRWAYS DISEASE)
- COAD, INFECTIVE EXACERBATION
- COPD
- COPD - INFECTIVE EXACERBATION
- COPD (CHRONIC OBSTRUCTIVE PULMONARY DISEASE)
- COPD, INFECTIVE EXACERBATION
- EMPHYSEMA
- EMPHYSEMA - INFECTIVE EXACERBATION
- INFECTIVE EXACERBATION OF CHRONIC BRONCHITIS
- INFECTIVE EXACERBATION OF COAD
- INFECTIVE EXACERBATION OF COPD

The following free text strings have been used to search for terms which may indicate records for inclusion:
- BRONCHITIS - CHRONIC
- BRONCHITIS, CHRONIC
- CHRONIC AIRWAYS LIMITATION
- CHRONIC BRONCHITIS
- CHRONIC OBSTRUCTIVE AIRWAYS DISEASE
- CHRONIC OBSTRUCTIVE PULMONARY DISEASE
- COAD
- COPD
- EMPHYSEMA

Records identified by a free text string alone are not automatically flagged, but are individually reviewed by a clinical coder to determine whether the text string actually refers to the condition indicated or is present in another context (eg, a search for 'cancer' may identify ‘partner died from cancer’). Each record is flagged accordingly.

Note: The conditions in this list were approved by NPS MedicineWise Medical Advisors in January 2019.

This condition flag was last updated on 01 January 2019.

2.17 Chronic Pain (f_PAIN_CHR)

This flag indicates those records where CHRONIC PAIN (or a relevant synonym) is reported in MedicineInsight either as a coded condition (selected from a list in the CIS) or as a free text entry in one or more of the 'Diagnosis', 'Reason for visit' or 'Reason for prescription' fields.

The following coded terms (from Docle or Pyefinch) have been used to identify records for inclusion:

- BACK PAIN SYNDROME
- CHRONIC PAIN
- CHRONIC PAIN - ASSOCIATED WITH SKIN GRAFT
- CHRONIC PAIN SYNDROME
- COMPLEX REGIONAL PAIN SYNDROME
- CRPS (COMPLEX REGIONAL PAIN SYNDROME)
- GRAFT - SKIN - CHRONIC PAIN
- PAIN - CHRONIC
- PAIN - CHRONIC - ASSOCIATED WITH SKIN GRAFT
- PAIN CLINIC REFERRAL
- PAIN SYNDROME - MYOFACIAL
- PAIN, CHRONIC
- REFERRAL TO PAIN CLINIC
- SKIN GRAFT - CHRONIC PAIN

The following free text strings have been used to search for terms which may indicate records for inclusion:

- CHRONIC PAIN
- CRONIC PAIN
- INTRACTABLE PAIN
- PAIN - CHRONIC
- PAIN CHRONIC
- PAIN INTRACTABLE
- PAIN REFERRAL
- PAIN SPECIALIST
- PAIN SYDNROME
- PAIN SYNDROME
- PAIN TEAM
- PAIN, CHRONIC

Records identified by a free text string alone are not automatically flagged, but are individually reviewed by a clinical coder to determine whether the text string actually refers to the condition indicated or is present in another context (eg, a search for 'cancer' may identify 'partner died from cancer'). Each record is flagged accordingly.

This condition flag was last updated in June 2019.

2.18 Coronary Heart Disease and Atherosclerosis (f_CHD_ATH)

This flag indicates those records where Coronary Heart Disease (or a relevant synonym) is reported in MedicineInsight either as a coded condition (using a drop down list in the CIS) or as a non-coded condition (free text) in one or more of the 'Diagnosis', 'Reason for visit' or 'Reason for prescription' fields.

The following coded terms (from Docle or Pyefinch) have been used to identify records for inclusion:
- AAA
- AAA RUPTURE
- ABDOMINAL AORTIC ANEURYSM
- ABDOMINAL AORTIC ANEURYSM RUPTURE
- ACS (ACUTE CORONARY SYNDROME)
- ACUTE CORONARY INSUFFICIENCY
- ACUTE CORONARY SYNDROME
- ACUTE MYOCARDIAL INFARCTION
- AMI
- AMI (ACUTE MYOCARDIAL INFARCTION)
- ANEURYSM OF ABDOMINAL AORTA
- ANEURYSM OF THORACIC AORTA
- ANGINA
- ANGINA PECTORIS
- ANGINA PECTORIS - UNSTABLE
- ANGINA, STABLE
- ANGINA, UNSTABLE
- ANTERIOR MYOCARDIAL INFARCT
- ANTEROLATERAL MYOCARDIAL INFARCT
- AORTOFEMORAL BYPASS OCCLUSION
- AORTOILIAC BYPASS OCCLUSION
- AORTOILIAC STENT BLOCKAGE
- AORTOILIAC STENT OCCLUSION
- ARTERIAL INSUFFICIENCY
- ARTERIOSCLEROTIC ARTERIAL INSUFFICIENCY
- ATHEROSCLEROTIC HEART DISEASE
- BLOCKAGE CORONARY ARTERY
- BLOCKED AORTOFEMORAL BYPASS
- BLOCKED AORTOILIAC BYPASS
- BLOCKED AORTOILIAC STENT
- BLOCKED CORONARY ARTERY BYPASS GRAFT
- BLOCKED FEMORO-POPLITEAL BYPASS
- BLOCKED POPLITEAL ARTERY STENT
- CHRONIC STABLE ANGINA
- CORONARY ARTERY BYPASS GRAFT BLOCKAGE
- CORONARY ARTERY BYPASS GRAFT OCCLUSION
- CORONARY ARTERY DISEASE
- CORONARY ARTERY STENT BLOCKED
- CORONARY HEART DISEASE
- CORONARY INSUFFICIENCY
- CORONARY OCCLUSION
- FEMORO-POPLITEAL BYPASS BLOCKAGE
- FEMORO-POPLITEAL BYPASS OCCLUSION

- HEART ATTACK
- HEART DISEASE, ATHEROSCLEROTIC
- HEART DISEASE, CORONARY
- HEART DISEASE, ISCHAEMIC
- IHD
- IHD (ISCHAEMIC HEART DISEASE)
- INFERIOR MYOCARDIAL INFARCTION
- ISCHAEMIC HEART DISEASE
- ISCHAEMIC VASCULAR DISEASE
- MI
- MYOCARDIAL DAMAGE
- MYOCARDIAL INFARCTION
- MYOCARDIAL INFARCTION - ANTERIOR
- MYOCARDIAL INFARCTION - ANTEROLATERAL
- MYOCARDIAL INFARCTION - INFERIOR
- MYOCARDIAL INFARCTION - POSTERIOR
- MYOCARDIAL INFARCTION - SILENT
- MYOCARDIAL INFARCTION - SUBENDOCARDIAL
- MYOCARDIAL INFARCTION - SUPERIOR
- MYOCARDIAL INFARCTION - WITH ST ELEVATION
- MYOCARDIAL INFARCTION - WITHOUT ST ELEVATION
- MYOCARDIAL INFARCTION, ANTERIOR
- MYOCARDIAL INFARCTION, ANTEROLATERAL
- MYOCARDIAL INFARCTION, INFERIOR
- MYOCARDIAL INFARCTION, NON STEMI
- MYOCARDIAL INFARCTION, POSTERIOR
- MYOCARDIAL INFARCTION, STEMI
- MYOCARDIAL INFARCTION, SUBENDOCARDIAL
- MYOCARDIAL INFARCTION, SUPERIOR
- MYOCARDIAL INSUFFICIENCY
- NON ST ELEVATION MYOCARDIAL INFARCTION
- NON-ST-ELEVATION MYOCARDIAL INFARCTION (NSTEMI)
- NSTEMI
- NSTEMI (NON-ST-ELEVATION MYOCARDIAL INFARCTION)
- OBSTRUCTED AORTOFEMORAL BYPASS
- OBSTRUCTED AORTOILIAC BYPASS
- OBSTRUCTED AORTOILIAC STENT
- OBSTRUCTED CORONARY ARTERY BYPASS GRAFT
- OBSTRUCTED FEMORO-POPLITEAL BYPASS
- OBSTRUCTED POPLITEAL ARTERY STENT
- OCCLUDED AORTOFEMORAL BYPASS
- OCCLUDED AORTOILIAC BYPASS
- OCCLUDED AORTOILIAC STENT
- OCCLUDED POPLITEAL ARTERY STENT
- OCCLUSION - CORONARY ARTERY
- OCCLUSION OF AORTIC BIFURCATION BYPASS GRAFT
- OCCLUSION OF FEMOROPOPLITEAL BYPASS GRAFT
- OCCLUSION, CORONARY ARTERY
- POPLITEAL ARTERY STENT BLOCKAGE
- POPLITEAL ARTERY STENT OCCLUSION
- POSTERIOR MYOCARDIAL INFARCT
- PREINFARCTION SYNDROME

- RUPTURE OF ABDOMINAL AORTIC ANEURYSM
- RUPTURED AAA
- SILENT MYOCARDIAL INFARCTION
- ST ELEVATION MYOCARDIAL INFARCTION
- STABLE ANGINA
- STEMI
- STEMI (ST-ELEVATION MYOCARDIAL INFARCTION)
- SUBENDOCARDIAL INFARCT
- SUBENDOCARDIAL MYOCARDIAL INFARCT
- SUPERIOR MYOCARDIAL INFARCT
- THORACIC AORTIC ANEURYSM
- UNSTABLE ANGINA
- UNSTABLE ANGINA - HIGH RISK
- UNSTABLE ANGINA - LOW RISK
- UNSTABLE ANGINA - MODERATE RISK

The following free text strings have been used to search for terms which may indicate records for inclusion:

- AAA
- ACUTE CORONARY SYNDROME
- ANEURYSM OF THORACIC AORTA
- ANGINA
- ANTERIOR MYOCARDIAL INFARCT
- ANTEROLATERAL MYOCARDIAL INFARCT
- AORTOFEMORAL BYPASS OCCLUSION
- AORTOILIAC BYPASS OCCLUSION
- AORTOILIAC STENT BLOCKAGE
- AORTOILIAC STENT OCCLUSION
- ARTERIAL INSUFFICIENCY
- ATHEROSCLEROTIC HEART DISEASE
- BLOCKAGE CORONARY ARTERY
- BLOCKED AORTOFEMORAL BYPASS
- BLOCKED AORTOILIAC BYPASS
- BLOCKED AORTOILIAC STENT
- BLOCKED CORONARY ARTERY BYPASS GRAFT
- BLOCKED FEMORO-POPLITEAL BYPASS
- BLOCKED POPLITEAL ARTERY STENT
- CORONARY ARTERY BYPASS GRAFT BLOCKAGE
- CORONARY ARTERY BYPASS GRAFT OCCLUSION
- CORONARY ARTERY DISEASE
- CORONARY ARTERY STENT BLOCKED
- CORONARY HEART DISEASE
- CORONARY INSUFFICIENCY
- CORONARY OCCLUSION
- FEMORO-POPLITEAL BYPASS BLOCKAGE
- FEMORO-POPLITEAL BYPASS OCCLUSION
- HEART ATTACK
- HEART DISEASE, ATHEROSCLEROTIC
- HEART DISEASE, CORONARY
- IHD
- MI
- MYOCARDIAL DAMAGE

- MYOCARDIAL INFARCTION
- MYOCARDIAL INSUFFICIENCY
- OBSTRUCTED AORTOFEMORAL BYPASS
- OBSTRUCTED AORTOILIAC BYPASS
- OBSTRUCTED AORTOILIAC STENT
- OBSTRUCTED CORONARY ARTERY BYPASS GRAFT
- OBSTRUCTED FEMORO-POPLITEAL BYPASS
- OBSTRUCTED POPLITEAL ARTERY STENT
- OCCLUDED AORTOFEMORAL BYPASS
- OCCLUDED AORTOILIAC BYPASS
- OCCLUDED AORTOILIAC STENT
- OCCLUDED POPLITEAL ARTERY STENT
- OCCLUSION - CORONARY ARTERY
- OCCLUSION OF AORTIC BIFURCATION BYPASS GRAFT
- OCCLUSION OF FEMOROPOPLITEAL BYPASS GRAFT
- OCCLUSION, CORONARY ARTERY
- POPLITEAL ARTERY STENT BLOCKAGE
- POPLITEAL ARTERY STENT OCCLUSION
- POSTERIOR MYOCARDIAL INFARCT
- POSTPERICARDIOTOMY SYNDROME
- PREINFARCTION SYNDROME
- SUBENDOCARDIAL INFARCT
- SUBENDOCARDIAL MYOCARDIAL INFARCT
- SUPERIOR MYOCARDIAL INFARCT
- THORACIC AORTIC ANEURYSM

Records identified by a free text string alone are not automatically flagged, but are individually reviewed by a clinical coder to determine whether the text string actually refers to the condition indicated or is present in another context (eg, a search for 'cancer' may identify ‘partner died from cancer’). Each record is flagged accordingly.

Note: The conditions in this list were approved by NPS MedicineWise Medical Advisors in January 2019.

This condition flag was last updated in January 2019.

2.19 Cough (f_COUGH)

This flag indicates those records where COUGH (or a relevant synonym) is reported in MedicineInsight either as a coded condition (selected from a list in the CIS) or as a free text entry in one or more of the 'Diagnosis', 'Reason for visit' or 'Reason for prescription' fields.

The following coded terms (from Docle or Pyefinch) have been used to identify records for inclusion:

- ACE INHIBITOR COUGH
- ALLERGIC COUGH
- BENIGN COUGH HEADACHE
- CHRONIC COUGH
- COUGH
- COUGH - BLOOD
- COUGH - CROUPY
- COUGH - NOCTURNAL
- COUGH - POST INFECTIVE
- COUGH - POST VIRAL
- COUGH - SWALLOWING
- COUGH DUE TO ACE INHIBITOR
- COUGH HEADACHE
- COUGH SYNCOPE
- COUGH, ALLERGIC
- COUGH, CHRONIC
- COUGH, HABIT
- COUGH, HABITUAL
- COUGH, NOCTURNAL
- COUGH, PAROXYSMAL
- COUGH, POST INFECTIVE
- COUGH, PSYCHOGENIC
- COUGH, SMOKERS’
- CROUPY COUGH
- FOOD INDUCED COUGHING
- HABIT COUGH
- HABITUAL COUGH
- HEADACHE, COUGH
- NOCTURNAL COUGH
- PAROXYSMAL COUGH
- POST BRONCHITIS COUGH
- POST INFECTIVE COUGH
- POST VIRAL COUGH
- POSTVIRAL COUGH
- POST-VIRAL COUGH
- PSYCHOGENIC COUGH
- SMOKERS’ COUGH
- SWALLOWING AND COUGH
- SYNCOPE - COUGH
- SYNCOPE, COUGH
- UPPER AIRWAY COUGH SYNDROME

- WHOOPING COUGH

The following free text strings have been used to search for terms which may indicate records for inclusion:
- COUGH

Records identified by a free text string alone are not automatically flagged, but are individually reviewed by a clinical coder to determine whether the text string actually refers to the condition indicated or is present in another context (eg, a search for 'cancer' may identify 'partner died from cancer'). Each record is flagged accordingly.

This condition flag was last updated in February 2020.

2.20 Crohn's Disease (f_CROHNS)

This flag indicates those records where Crohn's Disease (or a relevant synonym) is reported in MedicineInsight either as a coded condition (using a drop down list in the CIS) or as a non-coded condition (free text) in one or more of the 'Diagnosis', 'Reason for visit' or 'Reason for prescription' fields.

The following coded terms (from Docle or Pyefinch) have been used to identify records for inclusion:

- CROHN DISEASE
- CROHN'S DISEASE
- GRANULOMATOUS COLITIS
- REGIONAL ENTERITIS

The following text strings have been used to search for terms which may indicate records for inclusion:

- CHRON
- CHRONE
- CHRONS
- COLITIS
- CROHN DISEASE
- CROHN'S DISEASE
- CRONS
- ENTERITIS
- GRANULOMATOUS COLITIS
- REGIONAL ENTERITIS

Records identified by a free text string alone are not automatically flagged, but are individually reviewed by a clinical coder to determine whether the text string actually refers to the condition indicated or is present in another context (eg, a search for 'cancer' may identify 'partner died from cancer'). Each record is flagged accordingly.

This condition flag was last updated on 01 October 2018.

2.21 Deep venous thrombosis (f_DVT)

This flag indicates those records where Deep Vein Thrombosis (or a relevant synonym) is reported in MedicineInsight either as a coded condition (selected from a list in the CIS) or as a free text entry in one or more of the 'Diagnosis', 'Reason for visit' or 'Reason for prescription' fields.

The following coded terms (from Docle or Pyefinch) have been used to identify records for inclusion:
- DEEP VENOUS THROMBOSIS
- DVT
- THROMBOSIS - DEEP VEIN

The following free text strings have been used to search for terms which may indicate records for inclusion:
- D.V.T
- DEEP VENOUS THROMBOSIS
- DVT
- THROMBOSIS - DEEP VEIN
- VTE

Records identified by a free text string alone are not automatically flagged, but are individually reviewed by a clinical coder to determine whether the text string actually refers to the condition indicated or is present in another context (eg, a search for 'cancer' may identify 'partner died from cancer'). Each record is flagged accordingly.

This condition flag was last updated in October 2020.
This flag was copied into the RWR Condition Flag Dictionary on 22/20/2020.

2.22 Dementia (f_DEMEN)

This flag indicates those records where Dementia (or a relevant synonym) is reported in MedicineInsight either as a coded condition (using a drop down list in the CIS) or as a non-coded condition (free text) in one or more of the 'Diagnosis', 'Reason for visit' or 'Reason for prescription' fields.

The following coded terms (from Docle or Pyefinch) have been used to identify records for inclusion:

- ALZHEIMER'S DISEASE
- BINSWANGER DISEASE
- BINSWANGER'S ENCEPHALOPATHY
- DEMENTIA
- DEMENTIA - FRONTOTEMPORAL
- DEMENTIA - LEWY-BODY
- DEMENTIA - MULTI INFARCT
- DEMENTIA - PICK
- DEMENTIA - VASCULAR
- DEMENTIA RELATED PSYCHOSIS
- DEMENTIA WITH LEWY BODIES
- DEMENTIA, EARLY ONSET
- DEMENTIA, FRONTOTEMPORAL
- DEMENTIA, MULTI INFARCT
- DEMENTIA, PICK'S
- DEMENTIA, SEMANTIC
- DEMENTIA, SUBSTANCE INDUCED
- DEMENTIA, VASCULAR
- EARLY ONSET DEMENTIA
- FRONTOTEMPORAL DEMENTIA
- KORSAKOFF'S DEMENTIA
- KORSAKOFF'S PSYCHOSIS
- KORSAKOV'S PSYCHOSIS
- LEWY BODY DEMENTIA
- MAJOR NEUROCOGNITIVE DISORDER DUE TO ALZHEIMER'S DISEASE
- MULTI INFARCT DEMENTIA
- NEUROCOGNITIVE DISORDER, MAJOR, DUE TO ALZHEIMER'S DISEASE
- PARKINSON'S DISEASE - LEWY BODY DEMENTIA
- PICK'S DISEASE
- PSYCHOSIS - KORSAKOFF'S
- PSYCHOSIS, DEMENTIA RELATED
- SEMANTIC DEMENTIA
- SENILE DEMENTIA WITH PSYCHOSIS
- SUBCORTICAL ARTERIOSCLEROTIC ENCEPHALOPATHY
- SUBCORTICAL DEMENTIA
- SUBSTANCE INDUCED DEMENTIA
- VASCULAR DEMENTIA
- YOUNG ONSET DEMENTIA

The following free text strings have been used to search for terms which may indicate records for inclusion:

- ALZHEIMER
- BINSWANGER DISEASE

- BINSWANGER'S ENCEPHALOPATHY
- DEMEN
- DEMENTIA
- KORSAKOFF'S PSYCHOSIS
- KORSAKOV'S PSYCHOSIS
- MAJOR NEUROCOGNITIVE DISORDER DUE TO ALZHEIMER'S DISEASE
- NEUROCOGNITIVE DISORDER, MAJOR, DUE TO ALZHEIMER'S DISEASE
- PICK'S DISEASE
- PSYCHOSIS - KORSAKOFF'S
- SUBCORTICAL ARTERIOSCLEROTIC ENCEPHALOPATHY

Records identified by a free text string alone are not automatically flagged, but are individually reviewed by a clinical coder to determine whether the text string actually refers to the condition indicated or is present in another context (eg, a search for 'cancer' may identify 'partner died from cancer'). Each record is flagged accordingly.

Note: The conditions in this list were approved by NPS MedicineWise Medical Advisors in July 2019.

This condition flag was last updated in July 2019.

2.23 Depression (f_DEPR)

This flag indicates those records where Depression (or a relevant synonym) is reported in MedicineInsight either as a coded condition (using a drop down list in the CIS) or as a non-coded condition (free text) in one or more of the 'Diagnosis', 'Reason for visit' or 'Reason for prescription' fields.

The following coded terms (from Docle or Pyefinch) have been used to identify records for inclusion:
- ADJUSTMENT DISORDER (CHRONIC) WITH DEPRESSED AND ANXIOUS MOOD
- ADJUSTMENT DISORDER (CHRONIC) WITH DEPRESSED MOOD
- ADJUSTMENT DISORDER WITH DEPRESSED AND ANXIOUS MOOD
- ADJUSTMENT DISORDER WITH MIXED ANXIETY AND DEPRESSED MOOD
- ANXIETY/DEPRESSION
- CHRONIC ADJUSTMENT DISORDER WITH DEPRESSED AND ANXIOUS MOOD
- CHRONIC ADJUSTMENT DISORDER WITH DEPRESSED MOOD
- DEPRESSION
- DEPRESSION - ENDOGENOUS
- DEPRESSION - MINOR
- DEPRESSION - POST NATAL
- DEPRESSION - REACTIVE
- DEPRESSION - RECURRENT
- DEPRESSION - SUBSYNDROMAL
- DEPRESSION WITH MELANCHOLIC FEATURES
- DEPRESSION, ENDOGENOUS
- DEPRESSION, MELANCHOLIC
- DEPRESSION, NON MELANCHOLIC
- DEPRESSION, ORGANIC
- DEPRESSION, POSTNATAL
- DEPRESSION, PSYCHOTIC
- DEPRESSION, REACTIVE
- DEPRESSION/ANXIETY
- DEPRESSIVE ANXIETY DISORDER
- DEPRESSIVE EPISODE, MAJOR
- ENDOGENOUS DEPRESSION
- INSOMNIA - DEPRESSION-RELATED
- INVOLUTIONAL MELANCHOLIA
- MAJOR DEPRESSION
- MAJOR DEPRESSIVE EPISODE
- MELANCHOLIA
- MELANCHOLIA - INVOLUTIONAL
- MELANCHOLIC DEPRESSION
- MIXED ANXIETY DEPRESSION
- MIXED ANXIETY/DEPRESSIVE DISORDER
- MIXED DEPRESSION ANXIETY
- NEUROTIC DEPRESSION
- NON MELANCHOLIC DEPRESSION
- ORGANIC DEPRESSION
- POST NATAL DEPRESSION
- POSTNATAL DEPRESSION
- PSYCHOTIC DEPRESSION
- REACTIVE DEPRESSION

The following text strings have been used to search for terms which may indicate records for inclusion:

- ADJUSTMENT DISORDER (CHRONIC) WITH DEPRESSED AND ANXIOUS MOOD
- ADJUSTMENT DISORDER (CHRONIC) WITH DEPRESSED MOOD
- ADJUSTMENT DISORDER WITH DEPRESSED AND ANXIOUS MOOD
- CHRONIC ADJUSTMENT DISORDER WITH DEPRESSED MOOD
- DEPRES
- DEPRESSION
- DEPRESSIVE EPISODE, MAJOR

Records identified by a free text string alone are not automatically flagged, but are individually reviewed by a clinical coder to determine whether the text string actually refers to the condition indicated or is present in another context (eg, a search for 'cancer' may identify 'partner died from cancer'). Each record is flagged accordingly.

Note: The conditions in this list were approved by NPS MedicineWise Medical Advisors in January 2019.

This condition flag was last updated in January 2019.

2.24 Diabetes Mellitus Gestational (f_DM_GEST)

This flag indicates those records where Diabetes Mellitus, Gestational (or a relevant synonym) is reported in MedicineInsight either as a coded condition (using a drop down list in the CIS) or as a non-coded condition (free text) in one or more of the 'Diagnosis', 'Reason for visit' or 'Reason for prescription' fields.

The following coded terms (from Docle or Pyefinch) have been used to identify records for inclusion:
- DIABETES - GESTATIONAL
- DIABETES MELLITUS - GESTATIONAL
- DIABETES MELLITUS, GESTATIONAL
- GESTATIONAL DIABETES
- GESTATIONAL DIABETES MELLITUS

The following free text strings have been used to search for terms which may indicate records for inclusion:
- DIABETES - GESTATIONAL
- DIABETES MELLITUS - GESTATIONAL
- DIABETES MELLITUS, GESTATIONAL
- GESTATIONAL DIABETES

Records identified by a free text string alone are not automatically flagged, but are individually reviewed by a clinical coder to determine whether the text string actually refers to the condition indicated or is present in another context (eg, a search for 'cancer' may identify 'partner died from cancer'). Each record is flagged accordingly.

This condition flag was last updated in August 2018.

2.25 Diabetes Mellitus Type 1 (f_DM_T1)

This flag indicates those records where Diabetes Mellitus, Type 1 (or a relevant synonym) is reported in MedicineInsight either as a coded condition (using a drop down list in the CIS) or as a non-coded condition (free text) in one or more of the 'Diagnosis', 'Reason for visit' or 'Reason for prescription' fields.

The following coded terms (from Docle or Pyefinch) have been used to identify records for inclusion:

- DIABETES MELLITUS - IDDM
- DIABETES MELLITUS - TYPE I
- DIABETES MELLITUS, IDDM
- DIABETES MELLITUS, TYPE 1
- IDDM
- IDDM (INSULIN DEPENDENT DIABETES MELLITUS)
- INSULIN DEPENDENT DIABETES MELLITUS
- JUVENILE ONSET DIABETES
- JUVENILE ONSET DIABETES MELLITUS

The following text strings have been used to search for terms which may indicate records for inclusion:

- DIABETES MELLITUS - TYPE I
- DIABETES MELLITUS, TYPE 1
- IDDM
- INSULIN DEPENDENT DIABETES MELLITUS
- JUVENILE ONSET DIABETES
- TYPE 1 DIABETES MELLITUS

Records identified by a free text string alone are not automatically flagged, but are individually reviewed by a clinical coder to determine whether the text string actually refers to the condition indicated or is present in another context (eg, a search for 'cancer' may identify 'partner died from cancer'). Each record is flagged accordingly.

This condition flag was last updated on 01 August 2018.

2.26 Diabetes Mellitus Type 2 (f_DM_T2)

This flag indicates those records where Diabetes Mellitus, Type 2 (or a relevant synonym) is reported in MedicineInsight either as a coded condition (using a drop down list in the CIS) or as a non-coded condition (free text) in one or more of the 'Diagnosis', 'Reason for visit' or 'Reason for prescription' fields.

The following coded terms (from Docle or Pyefinch) have been used to identify records for inclusion:
- DIABETES MELLITUS - NIDDM
- DIABETES MELLITUS - TYPE II
- DIABETES MELLITUS, NIDDM
- DIABETES MELLITUS, TYPE 2
- DIABETES TYPE II REQUIRING INSULIN
- NIDDM
- NIDDM - REQUIRING INSULIN
- NIDDM (NON INSULIN DEPENDENT DIABETES MELLITUS)
- NON INSULIN DEPENDENT DIABETES MELLITUS
- T2DM
- TYPE 2 DIABETES MELLITUS

The following text strings have been used to search for terms which may indicate records for inclusion:

- DIABETES
- DIABETIC COMA - KETOACIDOTIC
- DIABETIC KETOACIDOTIC COMA
- T2DM

Records identified by a free text string alone are not automatically flagged, but are individually reviewed by a clinical coder to determine whether the text string actually refers to the condition indicated or is present in another context (eg, a search for 'cancer' may identify 'partner died from cancer'). Each record is flagged accordingly.

Note: The conditions in this list were approved by NPS MedicineWise Medical Advisors in January 2019.

This condition flag was last updated in January 2019.

2.27 Diabetes Mellitus Type 3 (f_DM_T3)

This flag indicates those records where Diabetes Mellitus - Type 3 (or a relevant synonym) is reported in MedicineInsight either as a coded condition (using a drop down list in the CIS) or as a non-coded condition (free text) in one or more of the 'Diagnosis', 'Reason for visit' or 'Reason for prescription' fields.

The following coded terms (from Docle or Pyefinch) have been used to identify records for inclusion:
- DIABETES MELLITUS, TYPE 3C
- PANCREATOGENIC DIABETES
- TYPE 3C DIABETES MELLITUS

The following free text strings have been used to search for terms which may indicate records for inclusion:
- DIABETES MELLITUS, TYPE 3C
- PANCREATOGENIC DIABETES
- TYPE 3C DIABETES MELLITUS

Records identified by a free text string alone are not automatically flagged, but are individually reviewed by a clinical coder to determine whether the text string actually refers to the condition indicated or is present in another context (eg, a search for 'cancer' may identify 'partner died from cancer'). Each record is flagged accordingly.

This condition flag was last updated in August 2018.

2.28 Diabetes Mellitus Unspecified (f_DM_US)

This flag indicates those records where Diabetes Mellitus, Unspecified (or a relevant synonym) is reported in MedicineInsight either as a coded condition (using a drop down list in the CIS) or as a non-coded condition (free text) in one or more of the 'Diagnosis', 'Reason for visit' or 'Reason for prescription' fields.

The following coded terms (from Docle or Pyefinch) have been used to identify records for inclusion:
- DIABETES
- DIABETES - CONTROLLED
- DIABETES - UNSTABLE
- DIABETES MELLITUS
- UNSTABLE DIABETES

The following text strings have been used to search for terms which may indicate records for inclusion:
- DIABETES
- DIABETES - CONTROLLED
- DIABETES - UNSTABLE
- DIABETES MELLITUS
- UNSTABLE DIABETES

Records identified by a free text string alone are not automatically flagged, but are individually reviewed by a clinical coder to determine whether the text string actually refers to the condition indicated or is present in another context (eg, a search for 'cancer' may identify 'partner died from cancer'). Each record is flagged accordingly.

DM_US is generally merged in with DM_T2 for a complete picture of patients with DM_T2

Note: The conditions in this list were approved by NPS MedicineWise Medical Advisors in January 2019.

This condition flag was last updated in January 2019.

2.29 Dyslipidaemia (f_DYS)

This flag indicates those records where Dyslipidaemia (or a relevant synonym) is reported in MedicineInsight either as a coded condition (using a drop down list in the CIS) or as a non-coded condition (free text) in one or more of the 'Diagnosis', 'Reason for visit' or 'Reason for prescription' fields.

The following coded terms (from Docle or Pyefinch) have been used to identify records for inclusion:
- DYSLIPIDAEMIA
- FAMILIAL HYPERCHOLESTEROLAEMIA
- FAMILIAL HYPERCHOLESTEROLEMIA
- HETEROZYGOUS FAMILIAL HYPERCHOLESTEROLEMIA
- HIGH CHOLESTEROL
- HOMOZYGOUS FAMILIAL HYPERCHOLESTEROLEMIA
- HYPERCHOLESTEROLAEMIA
- HYPERCHOLESTEROLAEMIA, FAMILIAL
- HYPERLIPIDAEMIA
- HYPERLIPIDAEMIA - CONTROLLED
- HYPERLIPIDAEMIA TYPE 2
- HYPERLIPOPROTEINAEMIA - TYPE2
- HYPERLIPOPROTEINAEMIA, TYPE 2
- HYPERLIPOPROTEINEMIA TYPE IV
- HYPERLIPOPROTEINEMIA, TYPE IIA
- HYPERTRIGLYCERIDAEMIA
- TYPE 2 HYPERLIPOPROTEINAEMIA

The following free text strings have been used to search for terms which may indicate records for inclusion:
- DYSLIP
- HDL
- HIGH CHOLEST
- HIGH LIPIDS
- HYPERCHO
- HYPERLIP
- HYPERTR

Records identified by a free text string alone are not automatically flagged, but are individually reviewed by a clinical coder to determine whether the text string actually refers to the condition indicated or is present in another context (eg, a search for 'cancer' may identify 'partner died from cancer'). Each record is flagged accordingly.

Note: The conditions in this list were approved by NPS MedicineWise Medical Advisors in January 2019.

This condition flag was last updated in January 2019.

2.30 Epilepsy (f_EPIL)

This flag indicates those records where Epilepsy (or a relevant synonym) is reported in MedicineInsight either as a coded condition (using a drop down list in the CIS) or as a non-coded condition (free text) in one or more of the 'Diagnosis', 'Reason for visit' or 'Reason for prescription' fields.

The following coded terms (from Docle or Pyefinch) have been used to identify records for inclusion:
- ABSENCE ATTACKS
- ABSENCES
- ACQUIRED EPILEPTIC APHASIA
- BENIGN ROLANDIC EPILEPSY
- COMPLEX PARTIAL SEIZURES
- CONVULSION
- DANCING EYES SYNDROME
- DRAVET SYNDROME
- EEG ABNORMAL
- EPILEPSY
- EPILEPSY - COMPLEX PARTIAL SEIZURES
- EPILEPSY - FEBRILE
- EPILEPSY - FOCAL
- EPILEPSY - GENERALISED
- EPILEPSY - GRAND MAL
- EPILEPSY - JACKSONIAN
- EPILEPSY - JUVENILE MYOCLONIC
- EPILEPSY - PETIT MAL
- EPILEPSY - POST-TRAUMATIC
- EPILEPSY - TEMPORAL LOBE
- EPILEPSY WITH POLYMORPHIC SEIZURES
- EPILEPSY, BENIGN ROLANDIC
- EPILEPSY, FOCAL
- EPILEPSY, GENERALISED
- EPILEPSY, GRAND MAL
- EPILEPSY, JACKSONIAN
- EPILEPSY, JUVENILE MYOCLONIC
- EPILEPSY, PETIT MAL
- EPILEPSY, POST TRAUMATIC
- EPILEPSY, PROGRESSIVE MYOCLONIC
- EPILEPSY, TEMPORAL LOBE
- EPILEPSY, TREATMENT RESISTANT
- EPILEPTIC FIT
- FEBRILE CONVULSIONS
- FEBRILE FITS
- FEBRILE SEIZURES
- FITS
- FOCAL EPILEPSY
- FOCAL FITS
- FOCAL SEIZURES
- FRONTAL LOBE EPILEPSY
- GENERALISED EPILEPSY
- GENERALISED FITS
- GENERALISED SEIZURES
- GENERALIZED FLEXION EPILEPSY

- GRAND MAL EPILEPSY
- GRAND MAL FIT
- GRAND MAL FITS
- GRAND MAL SEIZURES
- INFANTILE ACQUIRED APHASIA
- INFANTILE EPILEPTIC ENCEPHALOPATHY
- INFANTILE MYOCLONIC ENCEPHALOPATHY
- INFANTILE SPASMS
- JACKKNIFE CONVULSIONS
- JACKSONIAN EPILEPSY
- JACKSONIAN FITS
- JACKSONIAN MARCH
- JACKSONIAN SEIZURES
- JANZ SYNDROME
- JUVENILE MYOCLONIC EPILEPSY
- KINSBOURNE SYNDROME
- LAFORA DISEASE
- LAFORA PROGRESSIVE MYOCLONIC EPILEPSY
- LANDAU KLEFFNER SYNDROME
- MASSIVE MYOCLONIA
- MERFF SYNDROME
- MYOCLONIC ENCEPHALOPATHY OF INFANTS
- MYOCLONIC EPILEPSY WITH RAGGED RED FIBERS
- MYOCLONUS, POLYMYOCLONIA FAMILIAL ARRHYTHMIC
- OPSOCLONUS MYOCLONUS SYNDROME
- PARTIAL COMPLEX SEIZURES
- PETIT MAL
- PETIT MAL FITS
- PETIT MAL SEIZURES
- POLYMORPHIC EPILEPSY IN INFANCY
- POLYMYOCLONIA FAMILIAL ARRHYTHMIC MYOCLONUS
- POST TRAUMATIC EPILEPSY
- PRIMARY GENERALISED EPILEPSY
- PROGRESSIVE MYOCLONIC EPILEPSY
- PROGRESSIVE MYOCLONIC EPILEPSY, LAFORA TYPE
- PSYCHOMOTOR EPILEPSY
- PSYCHOMOTOR FITS
- PSYCHOMOTOR SEIZURES
- PYRIDOXINE DEPENDENCY SYNDROME
- PYRIDOXINE DEPENDENT EPILEPSY
- PYRIDOXINE DEPENDENT SEIZURES
- SALAAM SPASMS
- SEIZURES
- SEVERE MYOCLONIC EPILEPSY IN INFANCY
- SPIKE AND WAVE EPILEPSY
- STATUS EPILEPSY
- STATUS EPILEPTICUS
- TEMPORAL LOBE EPILEPSY
- TEMPORAL LOBE FITS
- TEMPORAL LOBE SEIZURES
- TONIC CLONIC EPILEPSY
- TONIC CLONIC FITS

- TONIC CLONIC SEIZURES
- TREATMENT RESISTANT EPILEPSY
- VITAMIN B6 RESPONSIVE EPILEPSY
- WEST SYNDROME

The following free text strings have been used to search for terms which may indicate records for inclusion:
- ABSENCE ATTACKS
- ABSENCES
- ACQUIRED EPILEPTIC APHASIA
- CONVULSION
- DANCING EYES SYNDROME
- DRAVET SYNDROME
- EEG ABNORMAL
- EPILEPSY
- EPILEPTIC FIT
- FITS
- GRAND MAL FIT
- INFANTILE ACQUIRED APHASIA
- INFANTILE EPILEPTIC ENCEPHALOPATHY
- INFANTILE MYOCLONIC ENCEPHALOPATHY
- INFANTILE SPASMS
- JACKSONIAN MARCH
- JANZ SYNDROME
- KINSBOURNE SYNDROME
- LAFORA DISEASE
- LANDAU KLEFFNER SYNDROME
- MASSIVE MYOCLONIA
- MERFF SYNDROME
- MYOCLONIC ENCEPHALOPATHY OF INFANTS
- MYOCLONUS, POLYMYOCLONIA FAMILIAL ARRHYTHMIC
- OPSOCLONUS MYOCLONUS SYNDROME
- PETIT MAL
- POLYMYOCLONIA FAMILIAL ARRHYTHMIC MYOCLONUS
- PYRIDOXINE DEPENDENCY SYNDROME
- SALAAM SPASMS
- SEIZURES
- STATUS EPILEPTICUS
- WEST SYNDROME

Records identified by a free text string alone are not automatically flagged, but are individually reviewed by a clinical coder to determine whether the text string actually refers to the condition indicated or is present in another context (eg, a search for 'cancer' may identify 'partner died from cancer'). Each record is flagged accordingly.

This condition flag was last updated in August 2018.

2.31 Gastro-oesophageal Reflux Disease (f_GORD)

This flag indicates those records where Gastro-oesophageal Reflux Disease (or a relevant synonym) is reported in MedicineInsight either as a coded condition (using a drop down list in the CIS) or as a non-coded condition (free text) in one or more of the 'Diagnosis', 'Reason for visit' or 'Reason for prescription' fields.

The following coded terms (from Docle or Pyefinch) have been used to identify records for inclusion:
- ACID REFLUX
- ACID REGURGITATION
- GASTRO-OESOPHAGEAL REFLUX
- GOR
- GOR (GASTRO-OESOPHAGEAL REFLUX)
- GORD
- GORD (GASTRO-OESOPHAGEAL REFLUX DISEASE)
- HEARTBURN
- LARYNGITIS, REFLUX
- LARYNGO PHARYNGEAL REFLUX
- LARYNGOPHARYNGEAL REFLUX
- NON-EROSIVE REFLUX DISEASE
- OESOPHAGEAL REFLUX
- OESOPHAGITIS, REFLUX
- REFLUX - GASTRO-OESOPHAGEAL
- REFLUX - LARYNGO PHARYNGEAL
- REFLUX LARYNGITIS
- REFLUX OESOPHAGITIS
- REFLUX, GASTRO-OESOPHAGEAL
- REFLUX, LARYNGOPHARYNGEAL

The following free text strings have been used to search for terms which may indicate records for inclusion:
- ACID REGURGITATION
- GER
- GOR
- HEARTBURN
- REFLUX

Records identified by a free text string alone are not automatically flagged, but are individually reviewed by a clinical coder to determine whether the text string actually refers to the condition indicated or is present in another context (eg, a search for 'cancer' may identify 'partner died from cancer'). Each record is flagged accordingly.

Note: Please be aware that the report mentioned in the internal purpose also relied on additional conditions (where PPI's were being prescribed) being flagged. These conditions are not included in this GORD definition.

This condition flag was last updated in August 2018.

2.32 Heart Failure (f_HF)

This flag indicates those records where Heart Failure (or a relevant synonym) is reported in MedicineInsight either as a coded condition (using a drop down list in the CIS) or as a non-coded condition (free text) in one or more of the 'Diagnosis', 'Reason for visit' or 'Reason for prescription' fields.

The following coded terms (from Docle or Pyefinch) have been used to identify records for inclusion:

- ACUTE CARDIAC FAILURE
- ACUTE HEART FAILURE
- BIVENTRICULAR HEART FAILURE
- CARDIAC FAILURE
- CARDIAC FAILURE, ACUTE
- CCF
- CHRONIC HEART FAILURE
- CONGESTIVE CARDIAC FAILURE
- CONGESTIVE HEART FAILURE
- COR PULMONALE
- DIASTOLIC CARDIAC DYSFUNCTION
- DIASTOLIC HEART FAILURE
- HEART FAILURE
- HEART FAILURE - ACUTE
- HEART FAILURE - BIVENTRICULAR
- HEART FAILURE - CHRONIC
- HEART FAILURE - HIGH OUTPUT
- HEART FAILURE - LEFT
- HEART FAILURE - MID RANGE EJECTION FRACTION
- HEART FAILURE - PRESERVED EJECTION FRACTION
- HEART FAILURE - REDUCED EJECTION FRACTION
- HEART FAILURE - RIGHT
- HEART FAILURE, ACUTE
- HEART FAILURE, HIGH OUTPUT
- HEART FAILURE, LEFT
- HFMREF
- HFPEF
- HFREF
- HIGH OUTPUT CARDIAC FAILURE
- HIGH OUTPUT HEART FAILURE
- HYPERTENSIVE HEART FAILURE
- LEFT HEART FAILURE
- LEFT VENTRICULAR FAILURE
- LHF
- LHF (LEFT HEART FAILURE)
- LVF
- LVF (LEFT VENTRICULAR FAILURE)
- PULMONARY OEDEMA
- RHF
- RHF (RIGHT HEART FAILURE)
- RIGHT HEART FAILURE
- RIGHT VENTRICULAR FAILURE
- RVF
- RVF (RIGHT VENTRICULAR FAILURE)

- SYSTOLIC CARDIAC DYSFUNCTION
- SYSTOLIC HEART FAILURE
- VENTRICULAR DIASTOLIC DYSFUNCTION

The following free text strings have been used to search for terms which may indicate records for inclusion:

- CARDIAC FAILURE
- CCF
- COR PULMONALE
- DIASTOLIC CARDIAC DYSFUNCTION
- HEART FAILURE
- HF
- HFMREF
- HFPEF
- HFREF
- LEFT VENTRICULAR FAILURE
- LHF
- LVF
- PULMONARY OEDEMA
- RHF
- RIGHT VENTRICULAR FAILURE
- RVF
- SYSTOLIC CARDIAC DYSFUNCTION
- VENTRICULAR DIASTOLIC DYSFUNCTION

Records identified by a free text string alone are not automatically flagged, but are individually reviewed by a clinical coder to determine whether the text string actually refers to the condition indicated or is present in another context (eg, a search for 'cancer' may identify 'partner died from cancer'). Each record is flagged accordingly.

Note: The conditions in this list were approved by NPS MedicineWise Medical Advisors in January 2019.

This condition flag was last updated in January 2019.

2.33 Hypercholesterolaemia (f_HYPERC)

This flag indicates those records where Hypercholesterolaemia (or a relevant synonym) is reported in MedicineInsight either as a coded condition (using a drop down list in the CIS) or as a non-coded condition (free text) in one or more of the 'Diagnosis', 'Reason for visit' or 'Reason for prescription' fields.

The following coded terms (from Docle or Pyefinch) have been used to identify records for inclusion:
- HIGH CHOLESTEROL
- HYPERCHOLESTEROLAEMIA

The following free text strings have been used to search for terms which may indicate records for inclusion:
- HIGH CHOLESTEROL
- HYPERCHOLESTEROLAEMIA

Records identified by a free text string alone are not automatically flagged, but are individually reviewed by a clinical coder to determine whether the text string actually refers to the condition indicated or is present in another context (eg, a search for 'cancer' may identify 'partner died from cancer'). Each record is flagged accordingly.

2.34 Hyperlipidaemia (f_HYPERLI)

This flag indicates those records where Hyperlipidaemia (or a relevant synonym) is reported in MedicineInsight either as a coded condition (using a drop down list in the CIS) or as a non-coded condition (free text) in one or more of the 'Diagnosis', 'Reason for visit' or 'Reason for prescription' fields.

The following coded terms (from Docle or Pyefinch) have been used to identify records for inclusion:
- DYSLIPIDAEMIA
- HYPERLIPIDAEMIA
- HYPERLIPIDAEMIA - CONTROLLED
- HYPERLIPIDAEMIA REVIEW
- HYPERLIPIDAEMIA TYPE 2
- HYPERLIPOPROTEINAEMIA TYPE 2
- REVIEW - HYPERLIPIDAEMIA
- TYPE 2 HYPERLIPOPROTEINAEMIA

The following free text strings have been used to search for terms which may indicate records for inclusion:
- DYSLIPIDAEMIA
- HYPERLIPIDAEMIA
- HYPERLIPOPROTEINAEMIA - TYPE2
- TYPE 2 HYPERLIPOPROTEINAEMIA

Records identified by a free text string alone are not automatically flagged, but are individually reviewed by a clinical coder to determine whether the text string actually refers to the condition indicated or is present in another context (eg, a search for 'cancer' may identify 'partner died from cancer'). Each record is flagged accordingly.

2.35 Hypertension (f_HYPT)

This flag indicates those records where Hypertension (or a relevant synonym) is reported in MedicineInsight either as a coded condition (using a drop down list in the CIS) or as a non-coded condition (free text) in one or more of the 'Diagnosis', 'Reason for visit' or 'Reason for prescription' fields.

The following coded terms (from Docle or Pyefinch) have been used to identify records for inclusion:
- HYPERTENSION - CONTROLLED
- ESSENTIAL HYPERTENSION
- HBP
- HIGH BLOOD PRESSURE
- HT (HYPERTENSION)
- HYPERTENSION
- PRIMARY HYPERTENSION
- HYPERTENSION - MALIGNANT
- MALIGNANT HYPERTENSION
- SEVERE REFRACTORY HYPERTENSION
- HYPERTENSION - PREGNANCY
- PIH
- PREGNANCY INDUCED HYPERTENSION
- HYPERTENSION - RENOVASCULAR
- RENAL HYPERTENSION
- RENOVASCULAR HYPERTENSION
- HYPERTENSION - ISOLATED SYSTOLIC
- BLOOD PRESSURE LABILE
- BP LABILE
- BP UNSTABLE
- HYPERTENSION - LABILE
- HYPERTENSION - UNSTABLE
- LABILE BLOOD PRESSURE
- LABILE BP
- LABILE HYPERTENSION
- HYPERTENSION - LIFE STYLE MANAGEMENT
- ANTIHYPERTENSIVE AGENT PRESCRIPTION
- BLOOD PRESSURE REVIEW
- HYPERTENSION REVIEW
- REVIEW - BP
- HYPERTENSION, ESSENTIAL
- HYPERTENSION, MALIGNANT
- HYPERTENSION IN PREGNANCY
- HYPERTENSION, RENOVASCULAR
- HYPERTENSION, ISOLATED SYSTOLIC
- ISOLATED SYSTOLIC HYPERTENSION
- DIASTOLIC HYPERTENSION
- HYPERTENSION, DIASTOLIC

The following free text strings have been used to search for terms which may indicate records for inclusion:
- BLOOD PRESSURE LABILE
- BLOOD PRESSURE REVIEW
- BP LABILE

- BP UNSTABLE
- H/T
- HBP
- HIGH BLOOD PRESSURE
- HIGH BP
- HT
- HYPER TENSION
- HYPERTENSION
- LABILE BLOOD PRESSURE
- LABILE BP
- PIH
- REVIEW - BP

Records identified by a free text string alone are not automatically flagged, but are individually reviewed by a clinical coder to determine whether the text string actually refers to the condition indicated or is present in another context (eg, a search for 'cancer' may identify 'partner died from cancer'). Each record is flagged accordingly.

Note: The conditions in this list were approved by NPS MedicineWise Medical Advisors in January 2019.

This condition flag was last updated in January 2019.

2.36 Hypertriglyceridemia (f_HYPERTRIG)

This flag indicates those records where Hypertriglyceridaemia (or a relevant synonym) is reported in MedicineInsight either as a coded condition (using a drop down list in the CIS) or as a non-coded condition (free text) in one or more of the 'Diagnosis', 'Reason for visit' or 'Reason for prescription' fields.

The following coded terms (from Docle or Pyefinch) have been used to identify records for inclusion:
- HYPERLIPOPROTEINAEMIA TYPE IV
- HYPERTRIGLYCERIDAEMIA

The following free text strings have been used to search for terms which may indicate records for inclusion:
- HYPERLIPOPROTEINAEMIA TYPE IV
- HYPERTRIGLYCERIDAEMIA

Records identified by a free text string alone are not automatically flagged, but are individually reviewed by a clinical coder to determine whether the text string actually refers to the condition indicated or is present in another context (eg, a search for 'cancer' may identify 'partner died from cancer'). Each record is flagged accordingly.

2.37 Influenza (f_FLU)

This flag indicates those records where Influenza (or a relevant synonym) is reported in MedicineInsight either as a coded condition (using a drop down list in the CIS) or as a non-coded condition (free text) in one or more of the 'Diagnosis', 'Reason for visit' or 'Reason for prescription' fields.

The following coded terms (from Docle or Pyefinch) have been used to identify records for inclusion:
- AVIAN INFLUENZA
- BIRD FLU
- FLU
- H1N1 INFLUENZA
- H5N1 INFLUENZA
- HUMAN SWINE INFLUENZA INFECTION
- INFECTION - INFLUENZA VIRUS
- INFECTION - PARAINFLUENZA
- INFECTION - PARAINFLUENZA 1 VIRUS
- INFECTION - PARAINFLUENZA 2 VIRUS
- INFECTION - PARAINFLUENZA 3 VIRUS
- INFLUENZA
- INFLUENZA A
- INFLUENZA A INFECTION
- INFLUENZA B
- INFLUENZA B INFECTION
- INFLUENZA H1N1
- INFLUENZA INFECTION
- PARA INFLUENZA 1 INFECTION
- PARA INFLUENZA 2 INFECTION
- PARA INFLUENZA 3 INFECTION
- PARAINFLUENZA INFECTION
- PARAINFLUENZA TYPE 1 INFECTION
- PARAINFLUENZA TYPE 2 INFECTION
- PARAINFLUENZA TYPE 3 INFECTION
- PIG FLU - H1N1 INFECTION
- SWINE FLU

The following free text strings have been used to search for terms which may indicate records for inclusion:
- FLU
- H1N1
- H5N5
- INFLUENZA
- PARAINFLUENZA
- SWINE

Records identified by a free text string alone are not automatically flagged, but are individually reviewed by a clinical coder to determine whether the text string actually refers to the condition indicated or is present in another context (eg, a search for 'cancer' may identify 'partner died from cancer'). Each record is flagged accordingly.

This condition flag was last updated in December 2018.

2.38 Influenza-like illness (f_ILI)

This flag indicates those records where Influenza-like illness (or a relevant synonym) is reported in MedicineInsight either as a coded condition (selected from a list in the CIS) or as a free text entry in one or more of the 'Diagnosis', 'Reason for visit' or 'Reason for prescription' fields.

The following coded terms (from Docle or Pyefinch) have been used to identify records for inclusion:
- INFLUENZA LIKE ILLNESS

The following free text strings have been used to search for terms which may indicate records for inclusion:
- FLU ILLNESS
- FLU LIKE
- FLU SYMPTOMS
- FLU TYPE
- FLULIKE
- FLU-LIKE
- INFLUENZA LIKE
- INFLUENZA-LIKE ILLNESS

Records identified by a free text string alone are not automatically flagged, but are individually reviewed by a clinical coder to determine whether the text string actually refers to the condition indicated or is present in another context (eg, a search for 'cancer' may identify 'partner died from cancer'). Each record is flagged accordingly.

This condition flag was last updated in December 2018.

2.39 Juvenile Rheumatoid Arthritis (f_ARTH_JRA)

This flag indicates those records where Juvenile Rheumatoid Arthritis (or a relevant synonym) is reported in MedicineInsight either as a coded condition (selected from a list in the CIS) or as a free text entry in one or more of the 'Diagnosis', 'Reason for visit' or 'Reason for prescription' fields.

The following coded terms (from Docle or Pyefinch) have been used to identify records for inclusion:
- ARTHRITIS - JUVENILE RHEUMATOID
- ARTHRITIS, JUVENILE RHEUMATOID
- JRA
- JRA (JUVENILE RHEUMATOID ARTHRITIS)
- JUVENILE RHEUMATOID ARTHRITIS
- RHEUMATOID ARTHRITIS - JUVENILE
- RHEUMATOID ARTHRITIS, JUVENILE
- STILLS DISEASE

The following free text strings have been used to search for terms which may indicate records for inclusion:
- JRA
- JUVENILE
- STILLS

Records identified by a free text string alone are not automatically flagged, but are individually reviewed by a clinical coder to determine whether the text string actually refers to the condition indicated or is present in another context (eg, a search for 'cancer' may identify 'partner died from cancer'). Each record is flagged accordingly.

This condition flag was last updated in June 2020.

2.40 Lower Back pain (f_PAIN_BACK_L)

This flag indicates those records where LOW BACK PAIN (or a relevant synonym) is reported in MedicineInsight either as a coded condition (selected from a list in the CIS) or as a free text entry in one or more of the 'Diagnosis', 'Reason for visit' or 'Reason for prescription' fields.

Note: Includes back pain with unspecified location. Excludes neck pain, cervical pain, thoracic pain etc.

The following coded terms (from Docle or Pyefinch) have been used to identify records for inclusion:
- BACK ACHE
- BACK AND BUTTOCK PAIN
- BACK AND LEG PAIN
- BACK INJURY
- BACK MUSCLE STRAIN
- BACK PAIN
- BACK PAIN - ACUTE
- BACK PAIN - ACUTE ON CHRONIC
- BACK PAIN - BUTTOCK
- BACK PAIN - DEGENERATIVE SPINE
- BACK PAIN - LEG
- BACK PAIN - LUMBAR
- BACK PAIN - LUMBO-SACRAL
- BACK PAIN - SACRAL
- BACK PAIN RADIATING TO BUTTOCK
- BACK PAIN RADIATING TO LEG
- BACK PAIN SYNDROME
- BACK PAIN WITH RADICULOPATHY
- BACK PAIN WITH REFERRED LEG PAIN
- BACK PAIN WITHOUT LEG PAIN
- BACK PAIN, LUMBO-SACRAL
- BACK PAIN, SACRAL
- BACK SPASM
- BACK STRAIN
- DEGENERATIVE DISC DISEASE, LUMBAR SPINE
- DEGENERATIVE LUMBAR DISC DISEASE
- FORAMINAL STENOSIS, LUMBAR
- INTERVERTEBRAL DISC PROLAPSE - L1
- INTERVERTEBRAL DISC PROLAPSE - L2
- INTERVERTEBRAL DISC PROLAPSE - L3
- INTERVERTEBRAL DISC PROLAPSE - L4
- INTERVERTEBRAL DISC PROLAPSE - L5
- L1 DISC PROLAPSE
- L2 DISC PROLAPSE
- L3 DISC PROLAPSE
- L4 DISC PROLAPSE
- L4 NERVE ROOT COMPRESSION
- L5 DISC PROLAPSE

- L5 NERVE ROOT COMPRESSION
- LOIN PAIN
- LOW BACK INJURY
- LOW BACK PAIN
- LOW BACK PAIN - MECHANICAL
- LOW BACK PAIN RADIATING TO BUTTOCK
- LOW BACK PAIN RADIATING TO LEG
- LOW BACK STRAIN
- LUMBAGO
- LUMBAR - PAIN
- LUMBAR BACK INJURY
- LUMBAR BACK MUSCLE STRAIN
- LUMBAR BACK PAIN
- LUMBAR DISC PROLAPSE
- LUMBAR NERVE ROOT COMPRESSION
- LUMBAR RADICULOPATHY
- LUMBAR SPONDYLOSIS
- LUMBAR SPRAIN
- LUMBO-SACRAL BACK PAIN
- LUMBO-SACRAL SPONDYLOSIS
- LUMBOSACRAL STENOSIS
- MECHANICAL BACK PAIN
- MECHANICAL LOW BACK PAIN
- MONONEUROPATHY - SCIATIC NERVE
- NERVE ROOT COMPRESSION, L4
- NERVE ROOT COMPRESSION, L5
- NERVE ROOT COMPRESSION, LUMBAR SPINE
- PAIN - BACK
- PAIN - BACK - LUMBAR
- PAIN - BACK - LUMBO-SACRAL
- PAIN - BACK - RADIATING TO BUTTOCK
- PAIN - BACK - RADIATING TO LEG
- PAIN - BACK - SACRAL
- PAIN IN LOW BACK
- PROLAPSED L1 DISC
- PROLAPSED L2 DISC
- PROLAPSED L3 DISC
- PROLAPSED L4 DISC
- PROLAPSED L5 DISC
- RADICULOPATHY - LUMBAR
- RADICULOPATHY, LUMBAR
- SACRAL SPINAL PAIN
- SACRO-ILIAC JOINT PAIN
- SCIATIC MONONEUROPATHY
- SCIATIC PAIN
- SCIATICA
- SCIATICA - L3/L4 DISC PROLAPSE
- SCIATICA - L4/L5 DISC PROLAPSE
- SCIATICA - L5/S1 DISC PROLAPSE

- SPINAL DISC PROTRUSION
- SPINAL PAIN
- SPINAL STENOSIS, LUMBAR
- SPONDYLOSIS, LUMBOSACRAL
- STRAIN - BACK MUSCLES
- STRAINED BACK

The following free text strings have been used to search for terms which may indicate records for inclusion:

- BACK ACHE
- BACK AND BUTTOCK PAIN
- BACK AND LEG PAIN
- BACK INJURY
- BACK MUSCLE STRAIN
- BACK PAIN
- BACK SPASM
- BACK STRAIN
- DEGENERATIVE DISC DISEASE, LUMBAR SPINE
- DEGENERATIVE LUMBAR DISC DISEASE
- FORAMINAL STENOSIS, LUMBAR
- INTERVERTEBRAL DISC PROLAPSE - L1
- INTERVERTEBRAL DISC PROLAPSE - L2
- INTERVERTEBRAL DISC PROLAPSE - L3
- INTERVERTEBRAL DISC PROLAPSE - L4
- INTERVERTEBRAL DISC PROLAPSE - L5
- L1 DISC PROLAPSE
- L2 DISC PROLAPSE
- L3 DISC PROLAPSE
- L4 DISC PROLAPSE
- L4 NERVE ROOT COMPRESSION
- L5 DISC PROLAPSE
- L5 NERVE ROOT COMPRESSION
- LOIN PAIN
- LUMBAGO
- LUMBAR - PAIN
- LUMBAR DISC PROLAPSE
- LUMBAR NERVE ROOT COMPRESSION
- LUMBAR RADICULOPATHY
- LUMBAR SPONDYLOSIS
- LUMBAR SPRAIN
- LUMBO-SACRAL SPONDYLOSIS
- LUMBOSACRAL STENOSIS
- NERVE ROOT COMPRESSION, L4
- NERVE ROOT COMPRESSION, L5
- NERVE ROOT COMPRESSION, LUMBAR SPINE
- PAIN - BACK - LUMBAR
- PAIN - BACK - LUMBO-SACRAL
- PAIN - BACK - RADIATING TO BUTTOCK
- PAIN - BACK - RADIATING TO LEG

- PAIN - BACK - SACRAL
- PAIN IN LOW BACK
- PROLAPSED L1 DISC
- PROLAPSED L2 DISC
- PROLAPSED L3 DISC
- PROLAPSED L4 DISC
- PROLAPSED L5 DISC
- RADICULOPATHY - LUMBAR
- RADICULOPATHY, LUMBAR
- SACRAL SPINAL PAIN
- SACRO-ILIAC JOINT PAIN
- SCIAT
- SPINAL DISC PROTRUSION
- SPINAL PAIN
- SPINAL STENOSIS, LUMBAR
- SPONDYLOSIS, LUMBOSACRAL
- STRAIN - BACK MUSCLES
- STRAINED BACK

Records identified by a free text string alone are not automatically flagged, but are individually reviewed by a clinical coder to determine whether the text string actually refers to the condition indicated or is present in another context (eg, a search for 'cancer' may identify 'partner died from cancer'). Each record is flagged accordingly.

This condition flag was last updated in August 2018.

2.41 Lower Respiratory Tract Infection (f_LRTI)

This flag indicates those records where LOWER RESPIRATORY TRACT INFECTION (or a relevant synonym) is reported in MedicineInsight either as a coded condition (selected from a list in the CIS) or as a free text entry in one or more of the 'Diagnosis', 'Reason for visit' or 'Reason for prescription' fields.

The following coded terms (from Docle or Pyefinch) have been used to identify records for inclusion:
- ACUTE BRONCHITIS
- ASPERGILLUS PNEUMONIA
- ASPIRATION PNEUMONIA
- ATYPICAL PNEUMONIA
- BRONCHIAL TUBERCULOSIS
- BRONCHIOLITIS
- BRONCHIOLITIS OBLITERANS ORGANISING PNEUMONIA
- BRONCHIOLITIS OBLITERANS ORGANIZING PNEUMONIA
- BRONCHITIS
- BRONCHITIS - ACUTE
- BRONCHITIS - BACTERIAL
- BRONCHITIS - CHRONIC
- BRONCHITIS - EOSINOPHILIC
- BRONCHITIS - RECURRENT
- BRONCHITIS - VIRAL
- BRONCHITIS, ACUTE
- BRONCHITIS, VIRAL
- BRONCHOPNEUMONIA
- CAVITATING TUBERCULOSIS IN LUNGS
- CAVITATING TUBERCULOSIS OF LUNG
- CHEST AIRWAY INFECTION
- CHEST INFECTION
- CHEST INFECTION - OF AIRWAY
- CHICKENPOX PNEUMONIA
- CHLAMYDIA PNEUMONIAE INFECTION
- CHLAMYDIAL PNEUMONIA
- CHLAMYDOPHILA PNEUMONIA
- CHLAMYDOPHILA PNEUMONIAE INFECTION
- CHRONIC BRONCHITIS
- CHRONIC BRONCHITIS - INFECTIVE EXACERBATION
- CHRONIC BRONCHITIS, INFECTIVE EXACERBATION
- CHRONIC FIBROUS PNEUMONIA
- COMMUNITY ACQUIRED PNEUMONIA
- COMPLICATED BRONCHITIS
- COUGH - CROUPY
- CROUP
- CROUP, SPASMODIC
- CROUP, VIRAL
- CROUPY COUGH
- CRYPTOGENIC ORGANIZING PNEUMONIA

- DOUBLE PNEUMONIA (DX FROM PATIENT)
- EOSINOPHILIC BRONCHITIS
- EOSINOPHILIC PNEUMONIA
- FIBROUS INTERSTITIAL PNEUMONIA
- GIANT CELL INTERSTITIAL PNEUMONIA
- HAEMOPHILUS PNEUMONIA
- IDIOPATHIC INTERSTITIAL PNEUMONIA
- INFECTION - CHLAMYDIA PNEUMONIAE
- INFECTION - STREPTOCOCCUS PNEUMONIAE
- INFECTIVE EXACERBATION OF CHRONIC BRONCHITIS
- INTERSTITIAL PNEUMONIA
- KLEBSIELLA PNEUMONIA
- LARYNGOTRACHEOBRONCHITIS
- LEFT LOWER LOBE PNEUMONIA
- LEFT UPPER LOBE PNEUMONIA
- LEGIONELLA PNEUMONIA
- LLL PNEUMONIA
- LOEFFLER PNEUMONIA
- LOWER RESPIRATORY TRACT INFECTION
- LOWER RESPIRATORY TRACT INFECTION - VIRAL
- LOWER RESPIRATORY TRACT INFECTION, VIRAL
- LRTI
- LRTI (LOWER RESPIRATORY TRACT INFECTION)
- LRTI, VIRAL
- LTB
- LUL PNEUMONIA
- METAPNEUMOVIRUS (MPV) PNEUMONIA
- MORAXELLA PNEUMONIA
- MYCOBACTERIUM TUBERCULOSIS INFECTION
- MYCOPLASMA PNEUMONIA
- MYCOPLASMA PNEUMONIA INFECTION
- MYCOPLASMA PNEUMONIAE SEROLOGY
- PLEURAL TUBERCULOSIS
- PNEUMOCOCCAL PNEUMONIA
- PNEUMOCYSTIS PNEUMONIA
- PNEUMONIA
- PNEUMONIA - ASPERGILLUS FUMIGATUS
- PNEUMONIA - ASPIRATION
- PNEUMONIA - CHLAMYDIA PNEUMONIAE
- PNEUMONIA - CHLAMYDIA PSITTACI
- PNEUMONIA - COMMUNITY ACQUIRED
- PNEUMONIA - CRYPTOGENIC ORGANIZING
- PNEUMONIA - EOSINOPHIL
- PNEUMONIA - HAEMOPHILUS INFLUENZAE
- PNEUMONIA - INTERSTITIAL
- PNEUMONIA - KLEBSIELLA PNEUMONIAE
- PNEUMONIA - LEGIONELLA PNEUMOPHILA
- PNEUMONIA - METAPNEUMOVIRUS (MPV)
- PNEUMONIA - MORAXELLA CATARRHALIS

- PNEUMONIA - MYCOPLASMA PNEUMONIAE
- PNEUMONIA - PNEUMOCYSTIS CARINII
- PNEUMONIA - RADIATION
- PNEUMONIA - RICKETTSIA BURNETTI
- PNEUMONIA - STAPHYLOCOCCUS AUREUS
- PNEUMONIA - STREPTOCOCCUS PNEUMONIAE
- PNEUMONIA - VIRAL
- PNEUMONIA, ASPERGILLUS
- PNEUMONIA, ASPIRATION
- PNEUMONIA, ATYPICAL
- PNEUMONIA, CHLAMYDIA PNEUMONIAE
- PNEUMONIA, CHLAMYDIA PSITTACI
- PNEUMONIA, COMMUNITY ACQUIRED
- PNEUMONIA, CRYPTOGENIC ORGANIZING
- PNEUMONIA, EOSINOPHILIC
- PNEUMONIA, HAEMOPHILUS INFLUENZAE
- PNEUMONIA, INTERSTITIAL
- PNEUMONIA, KLEBSIELLA PNEUMONIAE
- PNEUMONIA, LEFT LOWER LOBE
- PNEUMONIA, LEFT UPPER LOBE
- PNEUMONIA, LEGIONELLA PNEUMOPHILA
- PNEUMONIA, MORAXELLA CATARRHALIS
- PNEUMONIA, MYCOPLASMA
- PNEUMONIA, PNEUMOCYSTIS CARINII
- PNEUMONIA, RADIATION
- PNEUMONIA, RICKETTSIA BURNETTI
- PNEUMONIA, RIGHT LOWER LOBE
- PNEUMONIA, RIGHT MIDDLE LOBE
- PNEUMONIA, RIGHT UPPER LOBE
- PNEUMONIA, STAPHYLOCOCCAL
- PNEUMONIA, STREPTOCOCCUS PNEUMONIAE
- PNEUMONIA, USUAL INTERSTITIAL
- PNEUMONIA, VARICELLA
- PNEUMONIA, VIRAL
- PSITTACOSIS PNEUMONIA
- PULMONARY TUBERCULOSIS
- Q FEVER PNEUMONIA
- RADIATION PNEUMONIA
- RECURRENT BRONCHITIS
- RESPIRATORY TRACT INFECTION
- RIGHT LOWER LOBE PNEUMONIA
- RIGHT MIDDLE LOBE PNEUMONIA
- RIGHT UPPER LOBE PNEUMONIA
- RLL PNEUMONIA
- RML PNEUMONIA
- RTI (RESPIRATORY TRACT INFECTION)
- RUL PNEUMONIA
- SEROLOGY - MYCOPLASMA PNEUMONIAE
- SINO-BRONCHITIS

- SINO-BRONCHITIS - ACUTE
- SINO-BRONCHITIS - CHRONIC
- SPASMODIC CROUP
- STAPHYLOCOCCAL PNEUMONIA
- STREPTOCOCCUS PNEUMONIAE INFECTION
- TB (TUBERCULOSIS)
- TUBERCULOSIS
- TUBERCULOSIS - LUNG
- TUBERCULOSIS OF THE LUNG
- TUBERCULOSIS, PLEURAL
- UIP (USUAL INTERSTITIAL PNEUMONIA)
- USUAL INTERSTITIAL PNEUMONIA
- USUAL INTERSTITIAL PNEUMONIA (UIP)
- VARICELLA PNEUMONIA
- VIRAL BRONCHITIS
- VIRAL CROUP
- VIRAL LOWER RESPIRATORY TRACT INFECTION
- VIRAL LRTI
- VIRAL PNEUMONIA

The following free text strings have been used to search for terms which may indicate records for inclusion:

- BRONCHIAL SYNDROME
- BRONCHIOLITIS
- BRONCHITIS
- CHEST AIRWAY INFECTION
- CHEST INF
- CROUP
- LEGIONELLOSIS
- LEGIONNAIRE
- LOWER RESP TRACT
- LTB
- LUNG INFEC TION
- MYCOPLASMOSIS
- PNEUMONIA
- PNEUMONITIS
- RESPIRATORY TRACT INFECTION
- RTI
- TB
- TUBERCULOSIS

Records identified by a free text string alone are not automatically flagged, but are individually reviewed by a clinical coder to determine whether the text string actually refers to the condition indicated or is present in another context (eg, a search for 'cancer' may identify 'partner died from cancer'). Each record is flagged accordingly.

This condition flag was last updated in February 2020.

2.42 Osteoporosis (f_OP)

This flag indicates those records where Osteoporosis (or a relevant synonym) is reported in MedicineInsight either as a coded condition (using a drop down list in the CIS) or as a non-coded condition (free text) in one or more of the 'Diagnosis', 'Reason for visit' or 'Reason for prescription' fields.

The following coded terms (from Docle or Pyefinch) have been used to identify records for inclusion:

- OSTEOPOROSIS
- OSTEOPOROSIS - CORTICOSTEROID INDUCED
- OSTEOPOROSIS - NO FRACTURE
- OSTEOPOROSIS - PREVENTIVE CARE
- OSTEOPOROSIS WITH FRACTURE
- OSTEOPOROSIS, DISUSE
- OSTEOPOROSIS, STEROID INDUCED
- PATHOLOGICAL FRACTURE DUE TO OSTEOPOROSIS
- POST MENOPAUSAL OSTEOPOROSIS
- PREVENTIVE CARE - OSTEOPOROSIS
- STEROID INDUCED OSTEOPOROSIS

The following text strings have been used to search for terms which may indicate records for inclusion:

- OP
- OSTEOPOR
- OSTEOPOROSIS

Records identified by a free text string alone are not automatically flagged, but are individually reviewed by a clinical coder to determine whether the text string actually refers to the condition indicated or is present in another context (eg, a search for 'cancer' may identify 'partner died from cancer'). Each record is flagged accordingly.

This condition flag was last updated in August 2018.

2.43 Otitis media (f_OTITIS_M)

This flag indicates those records where OTITIS MEDIA (or a relevant synonym) is reported in MedicineInsight either as a coded condition (selected from a list in the CIS) or as a free text entry in one or more of the 'Diagnosis', 'Reason for visit' or 'Reason for prescription' fields.

The following coded terms (from Docle or Pyefinch) have been used to identify records for inclusion:

- BULLOUS MYRINGITIS
- MIDDLE EAR INFECTION
- MYRINGITIS - VIRAL
- MYRINGITIS BULLOSA
- OTITIS MEDIA
- OTITIS MEDIA, RECURRENT
- RECURRENT OTITIS MEDIA
- VIRAL MYRINGITIS

The following free text strings have been used to search for terms which may indicate records for inclusion:

- BULLOUS MYRINGITIS
- EAR EFFUSION
- EFFUSION - EAR
- GLUE EAR
- MIDDLE EAR INFECTION
- MYRINGITIS - VIRAL
- MYRINGITIS BULLOSA
- OTITIS MEDIA
- VIRAL MYRINGITIS

Records identified by a free text string alone are not automatically flagged, but are individually reviewed by a clinical coder to determine whether the text string actually refers to the condition indicated or is present in another context (eg, a search for 'cancer' may identify 'partner died from cancer'). Each record is flagged accordingly.

This condition flag was last updated in November 2018.

2.44 Peripheral vascular disease (f_PVD)

This flag indicates those records where Peripheral Vascular Disease (PVD) (or a relevant synonym) is reported in MedicineInsight either as a coded condition (using a drop down list in the CIS) or as a non-coded condition (free text) in one or more of the 'Diagnosis', 'Reason for visit' or 'Reason for prescription' fields.

The following coded terms (from Docle or Pyefinch) have been used to identify records for inclusion in PVD:
- ARTERIOSCLEROSIS OBLITERANS
- ARTERITIS - DIABETES MELLITUS
- BUERGER'S DISEASE
- DIABETES WITH VASCULAR CHANGES
- DIABETIC ENDARTERITIS
- DIABETIC PERIPHERAL VASCULAR DISEASE
- DIABETIC VASCULAR DISEASE - PERIPHERAL
- OBLITERATIVE VASCULAR DISEASE
- OCCLUSIVE VASCULAR DISEASE
- OCCLUSIVE VASCULAR DISEASE (BUERGER'S DISEASE)
- PERIPHERAL ARTERIAL DISEASE
- PERIPHERAL ARTERIAL OCCLUSIVE DISEASE (BUERGER'S DISEASE)
- PERIPHERAL VASCULAR DISEASE
- PERIPHERAL VASCULAR DISEASE, DIABETIC
- PVD
- THROMBANGIITIS OBLITERANS
- THROMBOANGIITIS OBLITERANS

The following free text strings have been used to search for terms which may indicate records for inclusion:
- ARTERIOSCLEROSIS OBLITERANS
- ARTERITIS - DIABETES MELLITUS
- BUERGER
- DIABETES WITH VASCULAR CHANGES
- DIABETIC ENDARTERITIS
- DIABETIC VASCULAR DISEASE - PERIPHERAL
- OBLITERATIVE VASCULAR DISEASE
- OCCLUSIVE VASCULAR DISEASE
- P.V.D
- PERIPHERAL ANGIOPATHY
- PERIPHERAL ARTERIAL DISEASE
- PERIPHERAL VASCULAR DISEASE
- PVD
- THROMBANGIITIS OBLITERANS
- THROMBOANGIITIS OBLITERANS

Records identified by a free text string alone are not automatically flagged, but are individually reviewed by a clinical coder to determine whether the text string actually refers to the condition indicated or is present in another context (eg, a search for 'cancer' may identify ‘partner died from cancer’). Each record is flagged accordingly.

This condition flag was last updated in January 2019.

2.45 Pertussis (f_PERTUSS)

This flag indicates those records where Pertussis (or a relevant synonym) is reported in MedicineInsight either as a coded condition (using a drop down list in the CIS) or as a non-coded condition (free text) in one or more of the 'Diagnosis', 'Reason for visit' or 'Reason for prescription' fields.

The following coded terms (from Docle or Pyefinch) have been used to identify records for inclusion:
- BORDETELLA PERTUSSIS - M&C
- BORDETELLA PERTUSSIS INFECTION
- INFECTION - BORDETELLA PERTUSSIS
- M&C - BORDETELLA PERTUSSIS
- PARAPERTUSSIS
- PERTUSSIS
- PERTUSSIS INFECTION
- WHOOPING COUGH

The following free text strings have been used to search for terms which may indicate records for inclusion:
- PERTUS
- PERTUSSIS
- WHOOPING
- WHOP
- WOOP

Records identified by a free text string alone are not automatically flagged, but are individually reviewed by a clinical coder to determine whether the text string actually refers to the condition indicated or is present in another context (eg, a search for 'cancer' may identify 'partner died from cancer'). Each record is flagged accordingly.

Note: The conditions in this list were approved by NPS MedicineWise Medical Advisors in January 2019.

This condition flag was last updated in December 2018.

2.46 Polycystic ovarian syndrome (f_PCOS)

This flag indicates those records where Polycystic Ovarian Syndrome (or a relevant synonym) is reported in MedicineInsight either as a coded condition (using a drop down list in the CIS) or as a non-coded condition (free text) in one or more of the 'Diagnosis', 'Reason for visit' or 'Reason for prescription' fields.

The following coded terms (from Docle or Pyefinch) have been used to identify records for inclusion:
- PCOS
- PCOS (POLYCYSTIC OVARIAN SYNDROME)
- POLYCYSTIC OVARIAN SYNDROME
- STEIN-LEVENTHAL SYNDROME

The following free text strings have been used to search for terms which may indicate records for inclusion:
- PCOS
- POLYCYSTIC OVARIAN SYNDROME
- POLYCYSTIC OVARY SYNDROME
- STEIN-LEVENTHAL SYNDROME

Records identified by a free text string alone are not automatically flagged, but are individually reviewed by a clinical coder to determine whether the text string actually refers to the condition indicated or is present in another context (eg, a search for 'cancer' may identify 'partner died from cancer'). Each record is flagged accordingly.

This condition flag was last updated in October 2020.
This flag was copied into the RWR Condition Flag Dictionary on 22/10/2020.

2.47 Prostatism (f_PROSTATISM)

This flag indicates those records where PROSTATISM (or a relevant synonym) is reported in MedicineInsight either as a coded condition (selected from a list in the CIS) or as a free text entry in one or more of the 'Diagnosis', 'Reason for visit' or 'Reason for prescription' fields.

The following coded terms (from Docle or Pyefinch) have been used to identify records for inclusion:

- ACUTE PROSTATITIS
- BENIGN PROSTATIC ENLARGEMENT
- BENIGN PROSTATIC HYPERPLASIA
- BENIGN PROSTATIC HYPERTROPHY
- BENIGN PROSTATOMEGALY
- BPH
- BPH (BENIGN PROSTATIC HYPERPLASIA)
- CHRONIC PROSTATITIS
- PROSTATIC HYPERTROPHY
- PROSTATISM
- PROSTATITIS
- PROSTATOMEGALY

The following free text strings have been used to search for terms which may indicate records for inclusion:

- BPH
- ENLARGED PROSTATE
- ENLARGED PROTRATE
- PROSTATE ENLARGEMENT
- PROSTATE HYPERTROPHY
- PROSTATIC ENLARGEMENT
- PROSTATIC HYPERPLASIA
- PROSTATIC HYPERTROPHY
- PROSTATIS
- PROSTATITI
- PROSTATITSM
- PROSTATOMEGALY
- PROSTITIS

Records identified by a free text string alone are not automatically flagged, but are individually reviewed by a clinical coder to determine whether the text string actually refers to the condition indicated or is present in another context (eg, a search for 'cancer' may identify 'partner died from cancer'). Each record is flagged accordingly.

This condition flag was last updated in January 2020.

2.48 Pulmonary Embolism (f_PE))

This flag indicates those records where Pulmonary embolism (or a relevant synonym) is reported in MedicineInsight either as a coded condition (selected from a list in the CIS) or as a free text entry in one or more of the 'Diagnosis', 'Reason for visit' or 'Reason for prescription' fields.

The following coded terms (from Docle or Pyefinch) have been used to identify records for inclusion:
- EMBOLISM - PULMONARY
- EMBOLISM, PULMONARY
- PULMONARY EMBOLISM
- PULMONARY EMBOLISM - SADDLE TYPE
- SADDLE PULMONARY EMBOLISM

The following free text strings have been used to search for terms which may indicate records for inclusion:
- EMBOLISM
- EMBOLUS
- PE
- PULM EMB
- PULMONARY EMB
- VTE

Note:
The abbreviation ‘PE’ can refer to many terms such as ‘Physical Examination’ , ‘Pre-eclampsia’, ‘Premature ejaculation’ etc. ‘PE’ was only assumed to refer to ‘Pulmonary embolism’ when reported with a related condition or other qualifier, or in conjunction with an anticoagulant eg

- Flagged as ‘Pulmonary embolism’ – WARFARIN FOR PE’, ‘CLEXANE FOR PE’, ‘DVT AND PE’, ‘SADDLE PE’, ‘LEFT LUNG PE’, 'UNPROVOKED PE', 'MALIGNANT PE'
- Not flagged as ‘Pulmonary embolism’ – ‘PE’, ‘POST OP PE’, ‘PE UNREMARKABLE’, ‘HYERTENSION POSSIBLE PE’, ‘PE ED LUTS’

Records identified by a free text string alone are not automatically flagged, but are individually reviewed by a clinical coder to determine whether the text string actually refers to the condition indicated or is present in another context (eg, a search for 'cancer' may identify 'partner died from cancer'). Each record is flagged accordingly.

This condition flag was last updated in October 2020.
This flag was copied into the RWR Condition Flag Dictionary on 22/10/2020.

2.49 Renal Artery Stenosis (f_RASTEN)

This flag indicates those records where Renal Artery Stenosis (or a relevant synonym) is reported in MedicineInsight either as a coded condition (using a drop down list in the CIS) or as a non-coded condition (free text) in one or more of the 'Diagnosis', 'Reason for visit' or 'Reason for prescription' fields.

The following coded terms (from Docle or Pyefinch) have been used to identify records for inclusion:

- ANEURYSM OF RENAL ARTERY
- BLOCKED RENAL ARTERY STENT
- OBSTRUCTED RENAL ARTERY STENT
- OCLUDED RENAL ARTERY STENT
- RENAL ARTERY ANEURYSM
- RENAL ARTERY OCCLUSION
- RENAL ARTERY STENOSIS
- RENAL ARTERY STENT BLOCKAGE
- RENAL ARTERY STENT OCCLUSION
- STENOSIS - RENAL ARTERY
- STENOSIS OF RENAL ARTERY

The following text strings have been used to search for terms which <u>may</u> indicate records for inclusion:

- ANEURYSM OF RENAL ARTERY
- BLOCKED RENAL ARTERY STENT
- OBSTRUCTED RENAL ARTERY STENT
- OCLUDED RENAL ARTERY STENT
- RENAL ARTERY ANEURYSM
- RENAL ARTERY OCCLUSION
- RENAL ARTERY STENOSIS
- RENAL ARTERY STENT BLOCKAGE
- RENAL ARTERY STENT OCCLUSION
- STENOSIS - RENAL ARTERY
- STENOSIS OF RENAL ARTERY

Records identified by a free text string alone are not automatically flagged, but are individually reviewed by a clinical coder to determine whether the text string actually refers to the condition indicated or is present in another context (eg, a search for 'cancer' may identify 'partner died from cancer'). Each record is flagged accordingly.

Note: The conditions in this list were approved by NPS MedicineWise Medical Advisors in January 2019.

This condition flag was last updated in January 2019.

2.50 Rheumatic Heart (f_RHEUHEAR)

This flag indicates those records where Rheumatic Heart Disease (or a relevant synonym) is reported in MedicineInsight either as a coded condition (selected from a list in the CIS) or as a free text entry in one or more of the 'Diagnosis', 'Reason for visit' or 'Reason for prescription' fields.

The following coded terms (from Docle or Pyefinch) have been used to identify records for inclusion:
- HEART DISEASE, POST-STREPTOCOCCAL
- HEART DISEASE, RHEUMATIC
- POST STREPTOCOCCAL HEART DISEASE
- POST STREPTOCOCCAL VALVE DISEASE
- RHEUMATIC HEART DISEASE
- RHEUMATIC MYOCARDITIS
- RHEUMATIC PANCARDITIS
- RHEUMATIC VALVE DISEASE

The following free text strings have been used to search for terms which may indicate records for inclusion:
- HEART DISEASE, POST-STREPTOCOCCAL
- POST STREPTOCOCCAL HEART DISEASE
- POST STREPTOCOCCAL VALVE DISEASE
- RHEUMATIC

Records identified by a free text string alone are not automatically flagged, but are individually reviewed by a clinical coder to determine whether the text string actually refers to the condition indicated or is present in another context (eg, a search for 'cancer' may identify 'partner died from cancer'). Each record is flagged accordingly.

This condition flag was last updated in March 2019.

2.51 Rheumatoid Arthritis (f_ARTH_RH)

This flag indicates those records where Rheumatoid Arthritis (or a relevant synonym) is reported in MedicineInsight either as a coded condition (using a drop down list in the CIS) or as a non-coded condition (free text) in one or more of the 'Diagnosis', 'Reason for visit' or 'Reason for prescription' fields.

The following coded terms (from Docle or Pyefinch) have been used to identify records for inclusion:
- ARTHRITIS - JUVENILE RHEUMATOID
- ARTHRITIS - RHEUMATOID
- ARTHRITIS, JUVENILE RHEUMATOID
- ARTHRITIS, RHEUMATOID
- ARTHRITIS, SERONEGATIVE
- AUTOANTIBODIES - RHEUMATOID FACTOR
- CAPLAN SYNDROME
- JRA
- JRA (JUVENILE RHEUMATOID ARTHRITIS)
- JUVENILE RHEUMATOID ARTHRITIS
- NODULES - RHEUMATOID
- RA
- RA (RHEUMATOID ARTHRITIS)
- RHEUMATOID ARTHRITIS
- RHEUMATOID ARTHRITIS - JUVENILE
- RHEUMATOID ARTHRITIS - PNEUMOCONIOSIS
- RHEUMATOID ARTHRITIS, JUVENILE
- RHEUMATOID FACTOR
- RHEUMATOID NODULES
- SERONEGATIVE ARTHRITIS
- SERONEGATIVE RHEUMATOID ARTHRITIS
- STILLS DISEASE

The following text strings have been used to search for terms which <u>may</u> indicate records for inclusion:
- ARTHRITIS - JUVENILE RHEUMATOID
- ARTHRITIS - RHEUMATOID
- ARTHRITIS, JUVENILE RHEUMATOID
- ARTHRITIS, RHEUMATOID
- ARTHRITIS, SERONEGATIVE
- CAPLAN SYNDROME
- NODULES - RHEUMATOID
- RA
- RHEUMATOID ARTHRITIS
- RHEUMATOID FACTOR
- RHEUMATOID NODULES
- SERONEGATIVE ARTHRITIS
- STILLS DISEASE

Records identified by a free text string alone are not automatically flagged, but are individually reviewed by a clinical coder to determine whether the text string actually refers to the condition indicated or is present in another context (eg, a search for 'cancer' may identify 'partner died from cancer'). Each record is flagged accordingly.

This condition flag was last updated on 01 August 2018.

2.52 Schizophrenia (f_SCHIZ)

This flag indicates those records where SCHIZOPHRENIA (or a relevant synonym) is reported in MedicineInsight either as a coded condition (selected from a list in the CIS) or as a free text entry in one or more of the 'Diagnosis', 'Reason for visit' or 'Reason for prescription' fields.

The following coded terms (from Docle or Pyefinch) have been used to identify records for inclusion:
- BORDERLINE SCHIZOPHRENIA
- BRIEF REACTIVE SCHIZOPHRENIA
- CATATONIC SCHIZOPHRENIA
- CHRONIC SCHIZOPHRENIA
- DISORGANISED SCHIZOPHRENIA
- HEBEPHRENIC SCHIZOPHRENIA
- PARA SCHIZOPHRENIA
- PARANOID SCHIZOPHRENIA
- PERSONALITY DISORDER - SCHIZOID
- PERSONALITY DISORDER - SCHIZOTYPAL
- PERSONALITY DISORDER, SCHIZOID
- PERSONALITY DISORDER, SCHIZOTYPAL
- RESIDUAL SCHIZOPHRENIA
- SCHIZOAFFECTIVE DISORDER
- SCHIZOID PERSONALITY DISORDER
- SCHIZOPHRENIA
- SCHIZOPHRENIA - BORDERLINE
- SCHIZOPHRENIA - BRIEF
- SCHIZOPHRENIA - CATATONIC
- SCHIZOPHRENIA - CHRONIC
- SCHIZOPHRENIA - PARANOID
- SCHIZOPHRENIA, BORDERLINE
- SCHIZOPHRENIA, CATATONIC
- SCHIZOPHRENIA, CHRONIC
- SCHIZOPHRENIA, DISORGANISED
- SCHIZOPHRENIA, HEBEPHRENIC
- SCHIZOPHRENIA, PARANOID
- SCHIZOPHRENIA, RESIDUAL
- SCHIZOPHRENIA, UNDIFFERENTIATED
- SCHIZOPHRENIFORM DISORDER
- SCHIZOTYPAL PERSONALITY DISORDER
- UNDIFFERENTIATED SCHIZOPHRENIA

The following free text strings have been used to search for terms which may indicate records for inclusion:
- PERSONALITY DISORDER - SCHIZOID
- PERSONALITY DISORDER - SCHIZOTYPAL
- PERSONALITY DISORDER, SCHIZOID
- PERSONALITY DISORDER, SCHIZOTYPAL
- SCHIZO
- SCHIZOID PERSONALITY DISORDER
- SCHIZOPHRENIA
- SCHIZOPHRENIFORM DISORDER
- SCHIZOTYPAL PERSONALITY DISORDER

Records identified by a free text string alone are not automatically flagged, but are individually reviewed by a clinical coder to determine whether the text string actually refers to the condition indicated or is present in another context (eg, a search for 'cancer' may identify 'partner died from cancer'). Each record is flagged accordingly.

This condition flag was last updated in November 2018.

2.53 Stroke (f_STROKE_ALL)

This flag indicates those records where Stroke (or a relevant synonym) is reported in MedicineInsight either as a coded condition (using a drop down list in the CIS) or as a non-coded condition (free text) in one or more of the 'Diagnosis', 'Reason for visit' or 'Reason for prescription' fields.

The following coded terms (from Docle or Pyefinch) have been used to identify records for inclusion in stroke:
- CEREBRAL HAEMORRHAGE
- CEREBRAL INFARCTION
- CEREBROVASCULAR ACCIDENT
- CVA
- CVA (CEREBROVASCULAR ACCIDENT)
- HAEMORRHAGE - INTRACEREBRAL
- HAEMORRHAGE, INTRACEREBRAL
- HAEMORRHAGIC CVA
- HAEMORRHAGIC STROKE
- INTRACEREBRAL BLEED
- INTRACEREBRAL HAEMORRHAGE
- INTRACRANIAL HAEMORRHAGE
- ISCHAEMIC STROKE
- LACUNAR INFARCT
- LACUNAR STROKE
- MIGRAINOUS STROKE
- MIGRANOUS STROKE
- STROKE
- STROKE - HAEMORRHAGIC
- STROKE - ISCHAEMIC
- STROKE - LACUNAR
- STROKE - MIGRANOUS
- STROKE - THROMBOTIC
- STROKE, HAEMORRHAGIC
- STROKE, ISCHAEMIC
- STROKE, LACUNAR
- STROKE, MIGRAINOUS
- STROKE, THROMBOTIC
- THROMBOTIC – STROKE
- THROMBOTIC STROKE
- VISUAL CORTEX STROKE

The following free text strings have been used to search for terms which may indicate records for inclusion:
- C.V.A
- CEREBRAL HAEMORRHAGE
- CEREBRAL INFARCTION
- CEREBROVASCULAR ACCIDENT
- CVA
- HAEMORRHAGE - INTRACEREBRAL
- HAEMORRHAGE, INTRACEREBRAL
- INTRACEREBRAL BLEED
- INTRACRANIAL HAEMORRHAGE

- LACUNAR INFARCT
- STROKE

Records identified by a free text string alone are not automatically flagged, but are individually reviewed by a clinical coder to determine whether the text string actually refers to the condition indicated or is present in another context (eg, a search for 'cancer' may identify ‘partner died from cancer’). Each record is flagged accordingly.

Note: The conditions in this list were approved by NPS MedicineWise Medical Advisors in January 2019.

Free text entries referring to a traumatic stroke caused by a fall or MVA for example are NOT included.

This condition flag was last updated in January 2019.

2.54 Stroke Haemorrhagic (f_STR_H)

This flag indicates those records where Stroke (or a relevant synonym) is reported in MedicineInsight either as a coded condition (using a drop down list in the CIS) or as a non-coded condition (free text) in one or more of the 'Diagnosis', 'Reason for visit' or 'Reason for prescription' fields.

The following coded terms (from Docle or Pyefinch) have been used to identify records for inclusion:
- CEREBRAL HAEMORRHAGE
- HAEMORRHAGE - INTRACEREBRAL
- HAEMORRHAGE, INTRACEREBRAL
- HAEMORRHAGIC CVA
- HAEMORRHAGIC STROKE
- INTRACEREBRAL BLEED
- INTRACEREBRAL HAEMORRHAGE
- INTRACRANIAL HAEMORRHAGE
- STROKE - HAEMORRHAGIC
- STROKE, HAEMORRHAGIC

The following text strings have been used to search for terms which <u>may</u> indicate records for inclusion:

- CEREBRAL HAEMORRHAGE
- HAEMORRHAGE - INTRACEREBRAL
- HAEMORRHAGE, INTRACEREBRAL
- HAEMORRHAGIC CVA
- HAEMORRHAGIC STROKE
- INTRACEREBRAL BLEED
- INTRACRANIAL HAEMORRHAGE
- STROKE - HAEMORRHAGIC
- STROKE, HAEMORRHAGIC

Records identified by a free text string alone are not automatically flagged, but are individually reviewed by a clinical coder to determine whether the text string actually refers to the condition indicated or is present in another context (eg, a search for 'cancer' may identify 'partner died from cancer'). Each record is flagged accordingly.

This condition flag was last updated on 01 August 2018.

2.55 Stroke Ischaemic (f_STR_I)

This flag indicates those records where Stroke (or a relevant synonym) is reported in MedicineInsight either as a coded condition (using a drop down list in the CIS) or as a non-coded condition (free text) in one or more of the 'Diagnosis', 'Reason for visit' or 'Reason for prescription' fields.

The following coded terms (from Docle or Pyefinch) have been used to identify records for inclusion:
- CEREBRAL INFARCTION
- ISCHAEMIC STROKE
- STROKE - ISCHAEMIC
- STROKE, ISCHAEMIC

The following text strings have been used to search for terms which may indicate records for inclusion:

- CEREBRAL INFARCTION
- ISCHAEMIC STROKE
- STROKE - ISCHAEMIC
- STROKE, ISCHAEMIC

Records identified by a free text string alone are not automatically flagged, but are individually reviewed by a clinical coder to determine whether the text string actually refers to the condition indicated or is present in another context (eg, a search for 'cancer' may identify 'partner died from cancer'). Each record is flagged accordingly.

This condition flag was last updated on 01 August 2018.

2.56 Stroke Lacunar (f_STR_L)

This flag indicates those records where Stroke (or a relevant synonym) is reported in MedicineInsight either as a coded condition (using a drop down list in the CIS) or as a non-coded condition (free text) in one or more of the 'Diagnosis', 'Reason for visit' or 'Reason for prescription' fields.

The following coded terms (from Docle or Pyefinch) have been used to identify records for inclusion:
- LACUNAR INFARCT
- LACUNAR STROKE
- STROKE - LACUNAR
- STROKE, LACUNAR

The following text strings have been used to search for terms which may indicate records for inclusion:

- LACUNAR INFARCT
- LACUNAR STROKE
- STROKE - LACUNAR
- STROKE, LACUNAR

Records identified by a free text string alone are not automatically flagged, but are individually reviewed by a clinical coder to determine whether the text string actually refers to the condition indicated or is present in another context (eg, a search for 'cancer' may identify 'partner died from cancer'). Each record is flagged accordingly.

This condition flag was last updated on 01 August 2018.

2.57 Stroke Migrainous (f_STR_M)

This flag indicates those records where Stroke (or a relevant synonym) is reported in MedicineInsight either as a coded condition (using a drop down list in the CIS) or as a non-coded condition (free text) in one or more of the 'Diagnosis', 'Reason for visit' or 'Reason for prescription' fields.

The following coded terms (from Docle or Pyefinch) have been used to identify records for inclusion:
- MIGRAINOUS STROKE
- MIGRANOUS STROKE
- STROKE - MIGRANOUS
- STROKE, MIGRAINOUS

The following text strings have been used to search for terms which may indicate records for inclusion:

- MIGRAINOUS STROKE
- MIGRANOUS STROKE
- STROKE - MIGRANOUS
- STROKE, MIGRAINOUS

Records identified by a free text string alone are not automatically flagged, but are individually reviewed by a clinical coder to determine whether the text string actually refers to the condition indicated or is present in another context (eg, a search for 'cancer' may identify 'partner died from cancer'). Each record is flagged accordingly.

This condition flag was last updated on 01 August 2018.

2.58 Stroke Thrombotic (f_STR_T)

This flag indicates those records where Stroke (or a relevant synonym) is reported in MedicineInsight either as a coded condition (using a drop down list in the CIS) or as a non-coded condition (free text) in one or more of the 'Diagnosis', 'Reason for visit' or 'Reason for prescription' fields.

The following coded terms (from Docle or Pyefinch) have been used to identify records for inclusion:

- STROKE - THROMBOTIC
- STROKE, THROMBOTIC
- THROMBOTIC - STROKE
- THROMBOTIC STROKE

The following text strings have been used to search for terms which <u>may</u> indicate records for inclusion:

- STROKE - THROMBOTIC
- STROKE, THROMBOTIC
- THROMBOTIC - STROKE
- THROMBOTIC STROKE

Records identified by a free text string alone are not automatically flagged, but are individually reviewed by a clinical coder to determine whether the text string actually refers to the condition indicated or is present in another context (eg, a search for 'cancer' may identify 'partner died from cancer'). Each record is flagged accordingly.

This condition flag was last updated on 01 August 2018.

2.59 Stroke Unspecified (f_STR_US)

This flag indicates those records where Stroke (or a relevant synonym) is reported in MedicineInsight either as a coded condition (using a drop down list in the CIS) or as a non-coded condition (free text) in one or more of the 'Diagnosis', 'Reason for visit' or 'Reason for prescription' fields.

The following coded terms (from Docle or Pyefinch) have been used to identify records for inclusion:

- CEREBRAL INFARCTION
- CEREBROVASCULAR ACCIDENT
- CVA
- CVA (CEREBROVASCULAR ACCIDENT)
- STROKE
- VISUAL CORTEX STROKE

The following text strings have been used to search for terms which may indicate records for inclusion:

- CEREBRAL INFARCTION
- CEREBROVASCULAR ACCIDENT
- CVA
- STROKE

Records identified by a free text string alone are not automatically flagged, but are individually reviewed by a clinical coder to determine whether the text string actually refers to the condition indicated or is present in another context (eg, a search for 'cancer' may identify 'partner died from cancer'). Each record is flagged accordingly.

This condition flag was last updated on 01 August 2018.

2.60 Substance Abuse (f_ABU_SUB)

This flag indicates those records where Substance Abuse (or a relevant synonym) is reported in MedicineInsight either as a coded condition (using a drop down list in the CIS) or as a non-coded condition (free text) in one or more of the 'Diagnosis', 'Reason for visit' or 'Reason for prescription' fields.

Note: this flag reflects a broad definition of substance abuse and includes smoking, alcohol social use, laxative abuse etc.

The following coded terms (from Docle or Pyefinch) have been used to identify records for inclusion:

- ABUSE - ALCOHOL
- ABUSE - AMPHETAMINE
- ABUSE - BENZODIAZEPINE
- ABUSE - CANNABIS
- ABUSE - COCAINE
- ABUSE - DRUG
- ABUSE - HALLUCINOGEN
- ABUSE - ICE (METHAMPHETAMINE)
- ABUSE - LAXATIVE
- ABUSE - LSD
- ABUSE - METHAMPHETAMINE
- ABUSE - NARCOTIC
- ABUSE - OPIATE
- ABUSE - POLYSUBSTANCE
- ABUSE - SUBSTANCE
- ABUSE - TOBACCO
- ABUSE - VOLATILE SOLVENT(S)
- ACUTE ALCOHOL WITHDRAWAL
- ACUTE INTOXICATION
- ALCOHOL ABUSE
- ALCOHOL ADDICTION
- ALCOHOL ASSESSMENT
- ALCOHOL CESSATION - TREATMENT
- ALCOHOL COUNSELLING
- ALCOHOL DEPENDENCE
- ALCOHOL EXCESS
- ALCOHOL INTOXICATION
- ALCOHOL MISUSE
- ALCOHOL OVERUSE
- ALCOHOL PREVENTIVE CARE
- ALCOHOL REDUCTION - TREATMENT
- ALCOHOL REFERRAL
- ALCOHOL RELATED BRAIN INJURY
- ALCOHOL SOCIAL USE
- ALCOHOL SUPPORT PLAN
- ALCOHOL USE DISORDER
- ALCOHOL WITHDRAWAL
- ALCOHOLIC
- ALCOHOLIC BINGE
- ALCOHOLISM

- AMPHETAMINE ABUSE
- AMPHETAMINE ADDICTION
- ANTABUSE REACTION
- ANTABUSE TYPE REACTION
- APC POWDER ABUSE
- BENZODIAZEPINE ABUSE
- BENZODIAZEPINE ADDICTION
- BENZODIAZEPINE DEPENDENCE
- CANNABIS ABUSE
- CANNABIS DEPENDENCE
- CANNABIS USE
- CIGARETTE ABUSE
- CIGARETTE DEPENDENCE
- COCAINE ABUSE
- COCAINE ADDICTION
- COCAINE DEPENDENCE
- DELIRIUM TREMENS
- DISULFIRAM REACTION
- DISULFIRAM TYPE REACTION
- DRUG ABUSE
- DRUG ABUSE - AMPHETAMINES
- DRUG ABUSE - CANNABIS
- DRUG ABUSE - ECSTASY
- DRUG ABUSE - ICE (METHAMPHETAMINE)
- DRUG ABUSE - OPIATES
- DRUG ADDICT
- DRUG ADDICTION
- DRUG DEPENDENCE
- DRUG OVERDOSE
- DRUG REACTION - DISULFIRAM
- DRUG SCREEN - URINARY
- DRUG SCREENING
- DRUG SEEKER
- DRUG SEEKING
- DRUG SEEKING BEHAVIOUR
- DRUG SOCIAL USE
- DRUG SUPPORT PLAN
- DRUG USE - PREVENTATIVE CARE
- DRUG USE INJURY
- DRUNK
- ETHANOL USE INJURY
- EXCESSIVE ALCOHOL INTAKE
- HALLUCINOGEN ABUSE
- HAZARDOUS DRINKING
- HEROIN ADDICTION
- HEROIN DEPENDENCE
- ICE (METHAMPHETAMINE) ABUSE
- IDU (INJECTING DRUG USER)
- IDU (INJECTING DRUG USER) IN PAST 6 MONTHS
- ILLICIT DRUG USE ASSESSMENT
- ILLICIT DRUG USE COUNSELLING
- ILLICIT DRUG USE REFERRAL

- INEBRIATED
- INJECTED DRUGS IN PAST SIX MONTHS
- INJECTING DRUG USER
- INTOXICATED
- INTRAVENOUS DRUG ABUSE
- INTRAVENOUS DRUG USE
- INTRAVENOUS DRUG(S) USE
- IV DRUG USE
- LAXATIVE ABUSE
- LONG TERM OPIATE USE
- LSD ABUSE
- MARIJUANA ADDICTION
- MARIJUANA USE
- METHAMPHETAMINE ABUSE
- MORPHINE ADDICTION
- MORPHINE DEPENDENCE
- NARCOTIC ABUSE
- NARCOTIC ADDICTION
- NARCOTIC DEPENDANCE
- NARCOTIC DEPENDENCE
- NEGATIVE URINE DRUG SCREEN TEST
- NICOTINE DEPENDANCE
- NICOTINE USE DISORDER
- OPIATE ABUSE
- OPIATE ADDICTION
- OPIATE DEPENDENCE
- OPIOID DEPENDANCE
- OPIOID USE DISORDER
- OVERDOSE, DRUG
- PETROL SNIFFING
- POLYSUBSTANCE ABUSE
- POLY-SUBSTANCE USE
- POSITIVE URINE DRUG SCREEN TEST
- PRESCRIPTION DRUG ABUSE
- PREVENTIVE CARE - ALCOHOL
- PROBLEM - ALCOHOL
- PROBLEM - DRUG ABUSE
- PROBLEM DRINKER
- PSEUDOEPHEDRINE ABUSE
- RECREATIONAL DRUG USAGE
- REFERRAL, ALCOHOL CONDITION
- SEEKING DRUGS
- SMOKING
- SOLVENT ABUSE
- SOLVENT DEPENDENCE
- SPEED ADDICTION
- SPEED USER
- SUBSTANCE ABUSE
- SUBSTANCE USE - POLY
- SUPPORT PLAN, ALCOHOL
- SUPPORT PLAN, DRUG
- TOBACCO ABUSE

- TOBACCO DEPENDENCE
- URINARY DRUG SCREEN
- URINE DRUG SCREEN TEST - NEGATIVE
- URINE DRUG SCREEN TEST - POSITIVE
- USER - ALCOHOL
- USER - DRUG
- VOLATILE SOLVENT DEPENDENCE
- WITHDRAWAL - ALCOHOL

The following free text strings have been used to search for terms which may indicate records for inclusion:

- ABUSE - DRUG
- ABUSE - LAXATIVE
- ABUSE - LSD
- ABUSE - NARCOTIC
- ABUSE - OPIATE
- ABUSE - POLYSUBSTANCE
- ABUSE - SUBSTANCE
- ABUSE - TOBACCO
- ABUSE - VOLATILE SOLVENT(S)
- ACUTE INTOXICATION
- ALCOHOL
- AMPHETAMINE
- ANTABUSE REACTION
- ANTABUSE TYPE REACTION
- APC POWDER ABUSE
- BENZODIAZEPINE
- CANNABIS
- CIGARETTE ABUSE
- CIGARETTE DEPENDENCE
- COCAINE
- DELIRIUM TREMENS
- DISULFIRAM REACTION
- DISULFIRAM TYPE REACTION
- DRUG ABUSE
- DRUG ADDICT
- DRUG DEPENDENCE
- DRUG OVERDOSE
- DRUG REACTION - DISULFIRAM
- DRUG SCREEN - URINARY
- DRUG SCREENING
- DRUG SEEKER
- DRUG SEEKING
- DRUG SOCIAL USE
- DRUG SUPPORT PLAN
- DRUG USE - PREVENTATIVE CARE
- DRUG USE INJURY
- DRUNK
- ETHANOL USE INJURY
- HALLUCINOGEN
- HAZARDOUS DRINKING
- HEROIN

- ILLICIT DRUG USE ASSESSMENT
- ILLICIT DRUG USE COUNSELLING
- ILLICIT DRUG USE REFERRAL
- INEBRIATED
- INJECTED DRUGS IN PAST SIX MONTHS
- INJECTING DRUG USER
- INTOXICATED
- INTRAVENOUS DRUG USE
- INTRAVENOUS DRUG(S) USE
- IV DRUG USE
- LAXATIVE ABUSE
- LONG TERM OPIATE USE
- LSD ABUSE
- MARIJUANA ADDICTION
- MARIJUANA USE
- MORPHINE ADDICTION
- MORPHINE DEPENDENCE
- NARCOTIC ABUSE
- NARCOTIC ADDICTION
- NARCOTIC DEPENDANCE
- NARCOTIC DEPENDENCE
- NEGATIVE URINE DRUG SCREEN TEST
- NICOTINE DEPENDANCE
- NICOTINE USE DISORDER
- OPIATE ABUSE
- OPIATE ADDICTION
- OPIATE DEPENDENCE
- OPIOID DEPENDANCE
- OPIOID USE DISORDER
- OVERDOSE, DRUG
- PETROL SNIFFING
- POLY-SUBSTANCE USE
- POSITIVE URINE DRUG SCREEN TEST
- PROBLEM DRINKER
- PSEUDOEPHEDRINE ABUSE
- RECREATIONAL DRUG USAGE
- SEEKING DRUGS
- SMOKING
- SOLVENT ABUSE
- SOLVENT DEPENDENCE
- SPEED ADDICTION
- SPEED USER
- SUBSTANCE ABUSE
- SUBSTANCE USE - POLY
- SUPPORT PLAN, DRUG
- TOBACCO ABUSE
- TOBACCO DEPENDENCE
- URINARY DRUG SCREEN
- URINE DRUG SCREEN TEST - NEGATIVE
- URINE DRUG SCREEN TEST - POSITIVE
- USER - DRUG

Records identified by a free text string alone are not automatically flagged, but are individually reviewed by a clinical coder to determine whether the text string actually refers to the condition indicated or is present in another context (eg, a search for 'cancer' may identify 'partner died from cancer'). Each record is flagged accordingly.

2.61 Transient Ischaemic Attack (f_TIA)

This flag indicates those records where TIA (or a relevant synonym) is reported in MedicineInsight either as a coded condition (using a drop down list in the CIS) or as a non-coded condition (free text) in one or more of the 'Diagnosis', 'Reason for visit' or 'Reason for prescription' fields.

The following coded terms (from Docle or Pyefinch) have been used to identify records for inclusion in TIA:

- ARTERIAL EMBOLISM - MINOR
- CEREBRAL TIA
- CEREBRAL TRANSIENT ISCHAEMIA
- CEREBRAL TRANSIENT ISCHAEMIC ATTACKS
- SYNCOPE, TIA
- TIA
- TIA (TRANSIENT ISCHAEMIC ATTACK)
- TRANSIENT ISCHAEMIC ATTACK

The following text strings have been used to search for terms which may indicate records for inclusion:

- ARTERIAL EMBOLISM - MINOR
- CEREBRAL TRANSIENT ISCHAEMIA
- T.I.A
- TIA
- TRANSIENT ISCHAEMIC ATTACK

Records identified by a free text string alone are not automatically flagged, but are individually reviewed by a clinical coder to determine whether the text string actually refers to the condition indicated or is present in another context (eg, a search for 'cancer' may identify ‘partner died from cancer’). Each record is flagged accordingly.

Note: The conditions in this list were approved by NPS MedicineWise Medical Advisors in January 2019.

This condition flag was last updated in January 2019.

2.62 Ulcerative Colitis (f_COLI_ULC)

This flag indicates those records where Ulcerative Colitis (or a relevant synonym) is reported in MedicineInsight either as a coded condition (using a drop down list in the CIS) or as a non-coded condition (free text) in one or more of the 'Diagnosis', 'Reason for visit' or 'Reason for prescription' fields.

The following coded terms (from Docle or Pyefinch) have been used to identify records for inclusion:
- COLITIS - ULCERATIVE
- COLITIS, ULCERATIVE
- ULCERATIVE COLITIS

The following free text strings have been used to search for terms which may indicate records for inclusion:
- COLITIS, ULCERATIVE
- COLLITIS
- ULCERATIVE
- ULCERATIVE COLITIS

Records identified by a free text string alone are not automatically flagged, but are individually reviewed by a clinical coder to determine whether the text string actually refers to the condition indicated or is present in another context (eg, a search for 'cancer' may identify 'partner died from cancer'). Each record is flagged accordingly.

This condition flag was last updated in August 2018.

nps.org.au
Level 7/418A Elizabeth Street Surry Hills NSW 2010
PO Box 1147 Strawberry Hills NSW 2012
02 8217 8700 02 9211 7578 info@nps.org.au

Independent, not-for-profit and evidence-based, NPS MedicineWise enables better decisions about medicines and medical tests. We receive funding from the Australian Government Department of Health. National Prescribing Service Limited. ABN 61 082 034 393. © NPS MedicineWise 2020

NPS MEDICINEWISE
