# Supplementary material for: Risk factor monitoring, management and use of prevention medicines in those with a history of premature coronary heart disease
Source: Open Heart. 2025 Sep 11;12(2):e003092. doi: 10.1136/openhrt-2024-003092 (PMC12519390; doi:10.1136/openhrt-2024-003092)
Supplement: online supplemental file 2 [file openhrt-12-2-s002.pdf]

Supplementary:

Supplementary Table 1: Medications

Drug class	Medication
ACE inhibitors	Captopril; Enalapril; Fosinopril; Lisinopril; Perindopril; Quinapril; Ramipril; Trandolapril
Angiotensin Receptor Blockers	Candesartan; Eprosartan; Irbesartan; Losartan; Olmesartan; Telmisartan; Valsartan
Beta-blockers	Atenolol; Bisoprolol; Carvedilol; Labetalol; Metoprolol; Nebivolol
Calcium channel blockers	Amlodipine; Clevidipine; Diltiazem; Felodipine; Lercanidipine; Nifedipine; Nimodipine; Verapamil
Thiazide and related diuretics	Chlortalidone; Hydrochlorothiazide; Indapamide
Other diuretics	Amiloride; Spironolactone
Other antihypertensives	Diazoxide; Hydralazine; Methyldopa; Minoxidil; Moxonidine; Prazosin
Statins	Atorvastatin, Fluvastatin, Pravastatin, Rosuvastatin, Simvastatin
Ezetimibe	Ezetimibe
Fibrates	Fenofibrate, Gemfibrozil
PCSK9 inhibitors	Alirocumab, Evolocumab
Other lipid lowering therapy	Cholestyramine, Nicotinic acid

Supplementary TABLE 2: RISK FACTORS ACROSS SEX

	Male					Female					Interaction model between sexes and premature of CHD
	CHD, N = 30,536[1]	95% CI[2]	PCHD, N = 10,696[1]	95% CI[2]	p-value[3]	CHD, N = 13,133[1]	95% CI[2]	PCHD, N = 10,339[1]	95% CI[2]	p-value[3]	p value[4]
Aboriginal and/or Torres Strait Islander	493 (1.6%)	1.5, 1.8%	526 (4.9%)	4.5, 5.3%	<0.001	186 (1.4%)	1.2, 1.6%	624 (6.0%)	5.6, 6.5%	<0.001	
Age	76 (70, 82)		60 (54, 68)		<0.001	82 (76, 87)		66 (59, 72)		<0.001	
Number of clinical encounters	106 (55, 183)		73 (36, 132)		<0.001	148 (78, 242)		105 (53, 188)		<0.001	0.624
TRADITIONAL MODIFIABLE RISK FACTORS:											
Smoking history					<0.001					<0.001	<0.001
Never	11,862 (40.2%)	39.6, 40.7%	3,455 (33.3%)	32.4, 34.3%		8,112 (64.3%)	63.5, 65.1%	4,821 (48.1%)	47.1, 49.1%		
Current or past	17,676 (59.8%)	59.3, 60.4%	6,909 (66.7%)	65.7, 67.6%		4,504 (35.7%)	34.9, 36.5%	5,203 (51.9%)	50.9, 52.9%		
Unknown	998		332			517		315			

Diabetes	9,640 (31.6%)	31.0, 32.1%	3,386 (31.7%)	30.8, 32.5%	0.398	3,470 (26.4%)	25.7, 27.2%	3,101 (30.0%)	29.1, 30.9%	<0.001	<0.001
Hypertension	21,505 (70.4%)	69.9, 70.9%	6,361 (59.5%)	58.5, 60.4%	<0.001	10,481 (79.8%)	79.1, 80.5%	6,650 (64.3%)	63.4, 65.2%	<0.001	<0.001
Dyslipidaemia	17,283 (56.6%)	56.0, 57.2%	6,207 (58.0%)	57.1, 59.0%	0.013	7,812 (59.5%)	58.6, 60.3%	5,956 (57.6%)	56.6, 58.6%	0.001	<0.001
RISK ENHANCING FACTORS AND COMORBIDITIES											
BMI category (highest)					<0.001					<0.001	0.407
Healthy weight range	3,195 (12.8%)	12.4, 13.2%	797 (9.2%)	8.6, 9.8%		2,084 (19.5%)	18.8, 20.3%	1,148 (13.5%)	12.8, 14.3%		
Obese	11,906 (47.7%)	47.1, 48.3%	5,134 (59.3%)	58.2, 60.3%		4,918 (46.0%)	45.1, 47.0%	5,105 (60.1%)	59.0, 61.1%		
Overweight	9,798 (39.3%)	38.7, 39.9%	2,707 (31.3%)	30.3, 32.2%		3,587 (33.6%)	32.7, 34.5%	2,184 (25.7%)	24.8, 26.7%		
Underweight	48 (0.2%)	0.1, 0.3%	23 (0.3%)	0.2, 0.4%		94 (0.9%)	0.7, 1.1%	58 (0.7%)	0.5, 0.9%		
Unknown	5,589		2,035			2,450		1,844			
Any substance abuse	1,325 (4.3%)	4.1, 4.6%	918 (8.6%)	8.1, 9.1%	<0.001	326 (2.5%)	2.2, 2.8%	617 (6.0%)	5.5, 6.4%	<0.001	0.024

Cancer	13,868 (45.4%)	44.9, 46.0%	2,757 (25.8%)	25.0, 26.6%	<0.001	5,915 (45.0%)	44.2, 45.9%	3,439 (33.3%)	32.4, 34.2%	<0.001	<0.001
Chronic Inflammatory Disease	3,502 (11.5%)	11.1, 11.8%	805 (7.5%)	7.0, 8.0%	<0.001	2,150 (16.4%)	15.7, 17.0%	1,465 (14.2%)	13.5, 14.9%	<0.001	<0.001
Chronic kidney disease (Stage 3-5)	1,915 (6.3%)	6.0, 6.6%	301 (2.8%)	2.5, 3.1%	<0.001	1,115 (8.5%)	8.0, 9.0%	444 (4.3%)	3.9, 4.7%	<0.001	0.296
Heart failure	4,488 (14.7%)	14.3, 15.1%	970 (9.1%)	8.5, 9.6%	<0.001	2,632 (20.0%)	19.4, 20.7%	1,056 (10.2%)	9.6, 10.8%	<0.001	
Peripheral vascular disease	2,303 (7.5%)	7.2, 7.8%	456 (4.3%)	3.9, 4.7%	<0.001	829 (6.3%)	5.9, 6.7%	426 (4.1%)	3.7, 4.5%	<0.001	0.048

[1] n (%); Median (IQR)

[2] CI = Confidence Interval

[3] random intercept logistic regression

[4]mixed logistic regression model (sex and pCHD) with clinic as a random effect

Supplementary Table 3: Multivariate model of significant predictors of beta blockers by GPs

Characteristic	OR[1]	95% CI[1]	p-value
Premature CHD	0.941	0.902, 0.982	0.005338140
Female	0.791	0.759, 0.825	0.000000000
First Nations Peoples	0.930	0.825, 1.048	0.233253306
Remoteness Area (2016)			
Outer Regional Australia	—	—	
Inner Regional Australia	0.932	0.840, 1.035	0.189358172
Major Cities of Australia	0.832	0.751, 0.923	0.000491230
(Very) Remote Australia	0.921	0.731, 1.161	0.486922031
BMI category (highest)			
Healthy weight range	—	—	
Obese	1.217	1.149, 1.290	0.000000000
Overweight	1.111	1.047, 1.178	0.000488966
Underweight	1.010	0.760, 1.343	0.944761768
Current/Past Smoker	1.019	0.979, 1.060	0.358997507

Hx Substance Abuse	0.989	0.907, 1.079	0.807566898
Hx Chronic Inflamm. Disease	0.978	0.924, 1.035	0.438040325
Hx Diabetes	1.328	1.272, 1.386	0.000000000
Hx Chronic Kidney Disease (3-5)	1.378	1.263, 1.503	0.000000000
Hx Dyslipidaemia	0.965	0.927, 1.004	0.081426952
Hx Hypertension	1.389	1.331, 1.450	0.000000000
Hx Heart Failure	3.436	3.210, 3.678	0.000000000

[1] OR = Odds Ratio, CI = Confidence Interval

Supplementary Table 4: Multivariate model of significant predictors of ACEI/ARB by GPs

Characteristic	OR[1]	95% CI[1]	p-value
Premature CHD	0.958	0.903, 1.016	0.152790200
Female	0.610	0.575, 0.647	0.000000000

First Nations Peoples	0.980	0.826, 1.163	0.813930895
Remoteness Area (2016)			
Outer Regional Australia	—	—	
Inner Regional Australia	1.034	0.904, 1.182	0.625831904
Major Cities of Australia	1.014	0.891, 1.154	0.836029531
(Very) Remote Australia	1.050	0.779, 1.415	0.749221979
BMI category (highest)			
Healthy weight range	—	—	
Obese	1.564	1.448, 1.690	0.000000000
Overweight	1.260	1.166, 1.362	0.000000004
Underweight	0.999	0.710, 1.407	0.996504366
Current/Past Smoker	1.090	1.031, 1.153	0.002574708
Hx Substance Abuse	0.957	0.847, 1.081	0.480653230
Hx Chronic Inflamm. Disease	0.979	0.900, 1.063	0.608825929
Hx Diabetes	1.964	1.838, 2.098	0.000000000
Hx Chronic Kidney Disease (3-5)	1.524	1.319, 1.762	0.000000012
Hx Dyslipidaemia	0.836	0.791, 0.885	0.000000000
Hx Hypertension	14.400	13.582, 15.268	0.000000000
Hx Heart Failure	2.968	2.682, 3.285	0.000000000

[1] OR = Odds Ratio, CI = Confidence Interval

Supplementary Table 5: Prescriptions to any non statin lipid lowering medications

Output of logistic regression predicting prescription to Any Non statin lipid lowering, with GP clinic as a random effect.

Characteristic	OR[1]	95% CI[1]	p-value
Premature CHD	0.656	0.514, 0.836	0.000654647
Female	0.915	0.730, 1.146	0.438064403
First Nations Peoples	0.705	0.346, 1.438	0.336209395
Remoteness Area (2016)			
Outer Regional Australia	—	—	
Major Cities of Australia	1.105	0.769, 1.587	0.590021342
Inner Regional Australia	1.057	0.717, 1.556	0.780454446
(Very) Remote Australia	1.134	0.427, 3.014	0.800572545
BMI category (highest)			
Healthy weight range	—	—	

Output of logistic regression predicting prescription to Any Non statin lipid lowering, with GP clinic as a random effect.

Characteristic	OR[1]	95% CI[1]	p-value
Obese	1.534	1.120, 2.101	0.007668055
Overweight	1.191	0.856, 1.657	0.298453200
Underweight	0.429	0.056, 3.276	0.414534106
Current/Past Smoker	0.999	0.799, 1.249	0.990204981
Hx Substance Abuse	0.552	0.314, 0.971	0.039293279
Hx Chronic Inflamm. Disease	0.792	0.576, 1.089	0.151642096
Hx Diabetes	2.635	2.058, 3.374	0.000000000
Hx Chronic Kidney Disease (3-5)	1.685	1.085, 2.618	0.020243565
Hx Dyslipidaemia	10.404	8.329, 12.996	0.000000000
Hx Hypertension	1.558	1.216, 1.996	0.000460295
Hx Heart Failure	0.770	0.557, 1.064	0.113582784

[1] OR = Odds Ratio, CI = Confidence Interval

Supplementary Table 6. **Prescriptions to secondary preventive medications.**

	Male					Female					
	CHD, N = 30,536[1]	95% CI[2]	PCHD, N = 10,696[1]	95% CI[2]	p-value[3]	CHD, N = 13,133[1]	95% CI[2]	PCHD, N = 10,339[1]	95% CI[2]	p-value[3]	Interaction p-value between sexes[4]
**Prescribed ACEI/ARB**	25,297 (82.8%)	82.4, 83.3%	8,401 (78.5%)	77.8, 79.3%	<0.001	10,751 (81.9%)	81.2, 82.5%	7,462 (72.2%)	71.3, 73.0%	<0.001	<0.001
**Prescribed Aspirin**	25,108 (82.2%)	81.8, 82.7%	8,563 (80.1%)	79.3, 80.8%	<0.001	10,074 (76.7%)	76.0, 77.4%	7,594 (73.5%)	72.6, 74.3%	<0.001	0.347
**Prescribed Beta blockers**	20,144 (66.0%)	65.4, 66.5%	6,954 (65.0%)	64.1, 65.9%	0.032	8,497 (64.7%)	63.9, 65.5%	5,851 (56.6%)	55.6, 57.5%	<0.001	<0.001
**Prescribed Statins**	28,185 (92.3%)	92.0, 92.6%	9,682 (90.5%)	89.9, 91.1%	<0.001	11,205 (85.3%)	84.7, 85.9%	8,577 (83.0%)	82.2, 83.7%	<0.001	0.432
**Prescribed to any non statin lipid-lowering therapy**	246 (10.5%)	9.3, 11.8%	76 (7.5%)	6.0, 9.3%	0.008	201 (10.4%)	9.1, 11.9%	111 (6.3%)	5.2, 7.6%	<0.001	0.312
**Prescribed to Ezetimibe**	173 (7.4%)	6.4, 8.5%	41 (4.0%)	3.0, 5.5%	<0.001	139 (7.2%)	6.1, 8.5%	74 (4.2%)	3.3, 5.3%	<0.001	0.856
**Prescribed to PCSK9 inhibitor**	8 (0.3%)	0.2, 0.7%	5 (0.5%)	0.2, 1.2%	0.523	5 (0.3%)	0.1, 0.6%	5 (0.3%)	0.1, 0.7%	0.892	0.535
**Prescribed to Fibrates**	109 (4.6%)	3.8, 5.6%	41 (4.0%)	3.0, 5.5%	0.382	78 (4.0%)	3.2, 5.0%	38 (2.2%)	1.6, 3.0%	0.001	0.076

Prescribed Cholestyramine or Nicotinic Acid	15 (0.6%)	0.4, 1.1%	0 (0.0%)	0.0, 0.5%	0.001	15 (0.8%)	0.5, 1.3%	9 (0.5%)	0.2, 1.0%	0.350	-
Prescribed Antiplatelets	14,575 (47.7%)	47.2, 48.3%	5,097 (47.7%)	46.7, 48.6%	0.838	5,244 (39.9%)	39.1, 40.8%	3,727 (36.0%)	35.1, 37.0%	<0.001	<0.001
Prescribed SA nitrates	14,249 (46.7%)	46.1, 47.2%	4,866 (45.5%)	44.5, 46.4%	0.026	6,962 (53.0%)	52.2, 53.9%	5,166 (50.0%)	49.0, 50.9%	<0.001	0.006
Prescribed Aspirin and/or Antiplatelets	26,624 (87.2%)	86.8, 87.6%	9,014 (84.3%)	83.6, 85.0%	<0.001	10,864 (82.7%)	82.1, 83.4%	8,112 (78.5%)	77.7, 79.2%	<0.001	0.369
Prescribed to any Anti-Hypertensive	28,409 (93.0%)	92.7, 93.3%	9,469 (88.5%)	87.9, 89.1%	<0.001	12,264 (93.4%)	92.9, 93.8%	8,852 (85.6%)	84.9, 86.3%	<0.001	<0.001

1 n (%)
2 CI = Confidence Interval
3 random intercept logistic regression
4 Mixed logistic regression (sex and pCHD) with clinic as a random effect
Some models failed to converge (due to having small sample sizes, for example) and are unreliable, so they are censored in the table.

Supplementary Figure 1: Patient’s included in dataset:

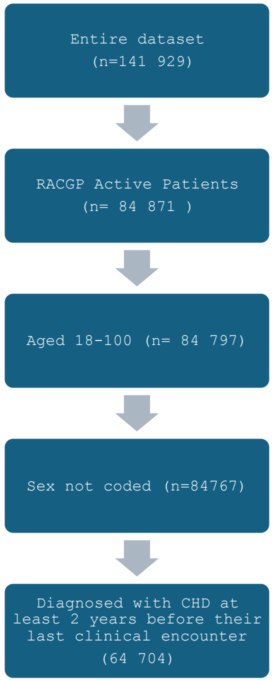

26

27
